# Smoking induces coordinated DNA methylation and gene expression changes in adipose tissue with consequences for metabolic health

**DOI:** 10.1186/s13148-018-0558-0

**Published:** 2018-10-20

**Authors:** Pei-Chien Tsai, Craig A. Glastonbury, Melissa N. Eliot, Sailalitha Bollepalli, Idil Yet, Juan E. Castillo-Fernandez, Elena Carnero-Montoro, Thomas Hardiman, Tiphaine C. Martin, Alice Vickers, Massimo Mangino, Kirsten Ward, Kirsi H. Pietiläinen, Panos Deloukas, Tim D. Spector, Ana Viñuela, Eric B. Loucks, Miina Ollikainen, Karl T. Kelsey, Kerrin S. Small, Jordana T. Bell

**Affiliations:** 1 Department of Twin Research and Genetic Epidemiology, King’s College London, London, SE1 7EH UK; 2 Department of Biomedical Sciences, Chang Gung University, Taoyuan, Taiwan; 3 Division of Allergy, Asthma, and Rheumatology, Department of Pediatrics, Chang Gung Memorial Hospital, Linkou, Taiwan; 4 Big Data Institute at the Li Ka Shing Centre for Health Information and Discovery, University of Oxford, Oxford, OX3 7LF UK; 5 Department of Epidemiology, Brown University School of Public Health, Providence, RI 02912 USA; 6 Institute for Molecular Medicine Finland (FIMM) and Department of Public Health, University of Helsinki, Helsinki, Finland; 7 Department of Bioinformatics, Institute of Health Sciences, Hacettepe University, 06100 Ankara, Turkey; 8 Pfizer - University of Granada - Andalusian Government Center for Genomics and Oncological Research (GENYO), Granada, Spain; 9 Division of Cancer Studies, King’s College London, London, SE1 9RT UK; 10 Department of Oncological Sciences, Icahn School of Medicine at Mount Sinai, New York City, NY 10029 USA; 11 The Tisch Cancer Institute, Icahn School of Medicine at Mount Sinai, New York City, NY 10029 USA; 12 Centre for Stem Cells and Regenerative Medicine, King’s College London, Floor 28, Tower Wing, Guy’s Hospital, Great Maze Pond, London, SE1 9RT UK; 13 NIHR Biomedical Research Centre at Guy’s and St Thomas’ Foundation Trust, London, SE1 9RT UK; 14 Research Programs Unit, Diabetes and Obesity, Obesity Research Unit, University of Helsinki, Helsinki, Finland; 15 Endocrinology, Abdominal Center, Helsinki University Hospital, Helsinki, Finland; 16 William Harvey Research Institute, Barts and The London School of Medicine and Dentistry, Queen Mary University of London, London, EC1M 6BQ UK; 17 Princess Al-Jawhara Al-Brahim Centre of Excellence in Research of Hereditary Disorders (PACER-HD), King Abdulaziz University, Jeddah, Saudi Arabia; 18 Department of Genetic Medicine and Development, University of Geneva Medical School, 1211 Geneva, Switzerland; 19 Institute for Genetics and Genomics in Geneva (iGE3), University of Geneva, 1211 Geneva, Switzerland; 20 Swiss Institute of Bioinformatics, 1211 Geneva, Switzerland; 21 Department of Laboratory Medicine & Pathology, Brown University, Providence, RI 02912 USA

**Keywords:** Smoking, DNA methylation, Gene expression, RNA-sequencing, Adipose tissue

## Abstract

**Background:**

Tobacco smoking is a risk factor for multiple diseases, including cardiovascular disease and diabetes. Many smoking-associated signals have been detected in the blood methylome, but the extent to which these changes are widespread to metabolically relevant tissues, and impact gene expression or metabolic health, remains unclear.

**Methods:**

We investigated smoking-associated DNA methylation and gene expression variation in adipose tissue biopsies from 542 healthy female twins. Replication, tissue specificity, and longitudinal stability of the smoking-associated effects were explored in additional adipose, blood, skin, and lung samples. We characterized the impact of adipose tissue smoking methylation and expression signals on metabolic disease risk phenotypes, including visceral fat.

**Results:**

We identified 42 smoking-methylation and 42 smoking-expression signals, where five genes (*AHRR*, *CYP1A1*, *CYP1B1*, *CYTL1*, *F2RL3*) were both hypo-methylated and upregulated in current smokers. *CYP1A1* gene expression achieved 95% prediction performance of current smoking status. We validated and replicated a proportion of the signals in additional primary tissue samples, identifying tissue-shared effects. Smoking leaves systemic imprints on DNA methylation after smoking cessation, with stronger but shorter-lived effects on gene expression. Metabolic disease risk traits such as visceral fat and android-to-gynoid ratio showed association with methylation at smoking markers with functional impacts on expression, such as *CYP1A1*, and at tissue-shared smoking signals, such as *NOTCH1*. At smoking-signals, *BHLHE40* and *AHRR* DNA methylation and gene expression levels in current smokers were predictive of future gain in visceral fat upon smoking cessation.

**Conclusions:**

Our results provide the first comprehensive characterization of coordinated DNA methylation and gene expression markers of smoking in adipose tissue. The findings relate to human metabolic health and give insights into understanding the widespread health consequence of smoking outside of the lung.

**Electronic supplementary material:**

The online version of this article (10.1186/s13148-018-0558-0) contains supplementary material, which is available to authorized users.

## Background

Tobacco smoking is a major environmental risk factor that predisposes an individual to chronic disease, cancer, and premature death [1, 2]. Smoking directly affects exposed regions of the lung [3], causes damage in organs throughout the body, and results in DNA mutations that have been linked to cancer [4]. The risk effects of smoking extend to multiple diseases, including cardiovascular and metabolic disease. Smoking cessation has also been linked to metabolic health complications and is associated with an increase in weight gain and in metabolic disease risk factors such as accumulation of visceral fat [5].

Persistent smoking has lasting effects on DNA methylation, and many epigenome-wide association studies (EWAS) have identified and replicated smoking-related differentially methylated signals across populations with the majority found in whole blood samples [6–20], buccal cells [21], and lung tissue [22, 23]. Most smoking methylation signals show lower levels of DNA methylation in current smokers compared to non-smokers, and variable dynamics upon cessation. Although some alterations persist over decades, smoking cessation can result in methylation levels reverting to those observed in non-smokers [13, 16, 18, 24]. However, most ex-smokers exhibit intermediate methylation levels between non-smokers and current smokers [13, 16, 18, 24]. Methylation levels correlate with the cumulative dose of smoking and are associated with time since smoking cessation [13, 16, 24, 25].

Smoking can also affect gene expression, as reported in the human airway epithelium [26, 27], lung tissue [28], alveolar macrophages [29], and lung cancer tissue [30]. However, few studies have examined DNA methylation and gene expression changes concurrently, and these studies were either conducted with low coverage genome assays (such as pyrosequencing [30] and HELP assay [8]) or targeted single genes of interest in small sample sizes [8, 30].

Here, we performed the first combined genome-wide analysis of smoking-related methylation and gene expression changes across tissues, focusing on adipose tissue. Exploring the molecular changes induced by smoking in a metabolically relevant tissue such as adipose tissue is of value to metabolic health research, because smoking is a risk factor for metabolic complications and smoking cessation has been linked to the accumulation of visceral fat. Here, we identify multiple genes that exhibit both methylation and expression changes within adipose tissue and across tissues, showing that smoking leaves a systemic imprint on DNA methylation and expression variation in the human body. Our data suggest that smoking leaves a stronger impact on gene expression, while DNA methylation smoking changes are more stable over time. By linking our findings to key human phenotypes related to metabolic health, we identify signals that could add understanding to some of the wide-ranging risk effects of smoking on metabolic diseases.

## Results

### Integrated DNA methylation and gene expression analyses in adipose tissue

**Fig. 1:**
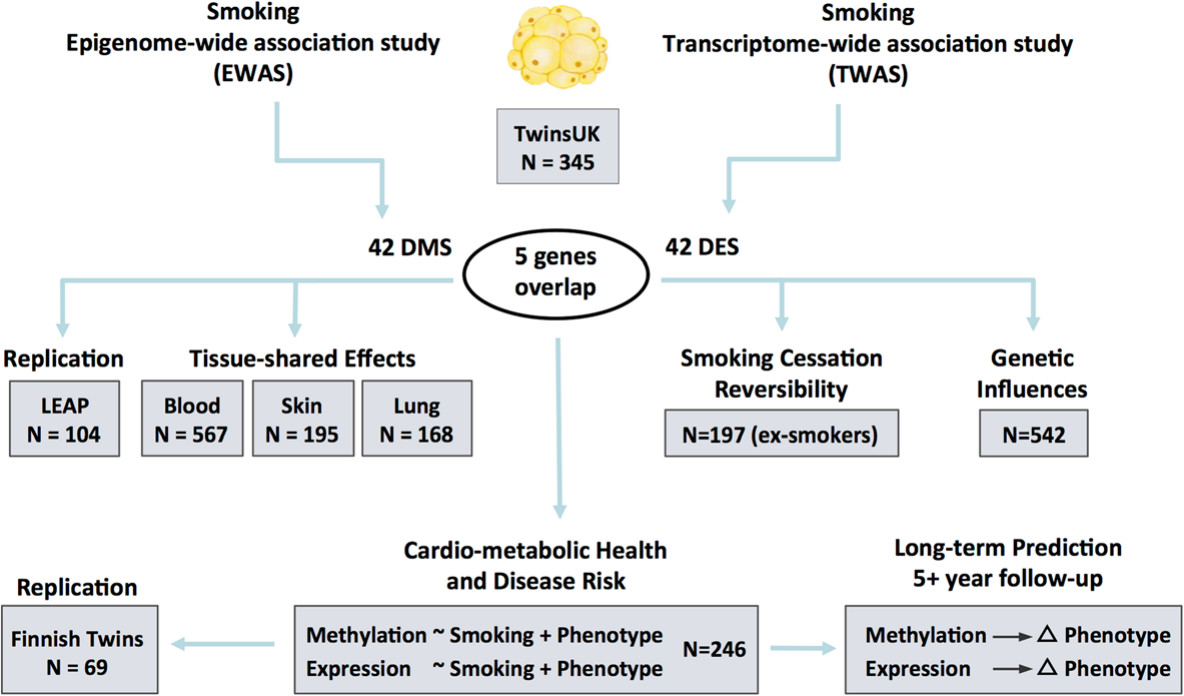
Study design. Epigenome-wide and transcriptome-wide association studies were performed in 345 adipose tissue samples, identifying 42 smoking-DMS and 42 smoking-DES where five genes (14 CpG sites) overlapped. The 42 smoking-DMS were replicated in 104 independent subjects from the LEAP cohort, and the 14 smoking-DMS were further explored in blood, skin, and lung tissue for tissue-shared effects. DNA methylation and gene expression profiles at the 42 smoking-DMS and 42 smoking-DES were tested for smoking cessation reversibility in 197 ex-smokers. Heritability and QTL analyses testing genetic and environmental influences on methylation in the 542 adipose samples were also carried out. The final set of analyses focused on exploring the link between the 42 smoking-DMS and 42 smoking-DES with metabolic phenotypes. Phenotype associations with smoking-DMS were replicated in 69 Finnish twins. The last set of analyses explored the potential of methylation and gene expression levels at smoking-DMS and smoking-DES to predict future long-term changes in adiposity phenotypes in individuals who go on to quit smoking

**Fig. 2:**
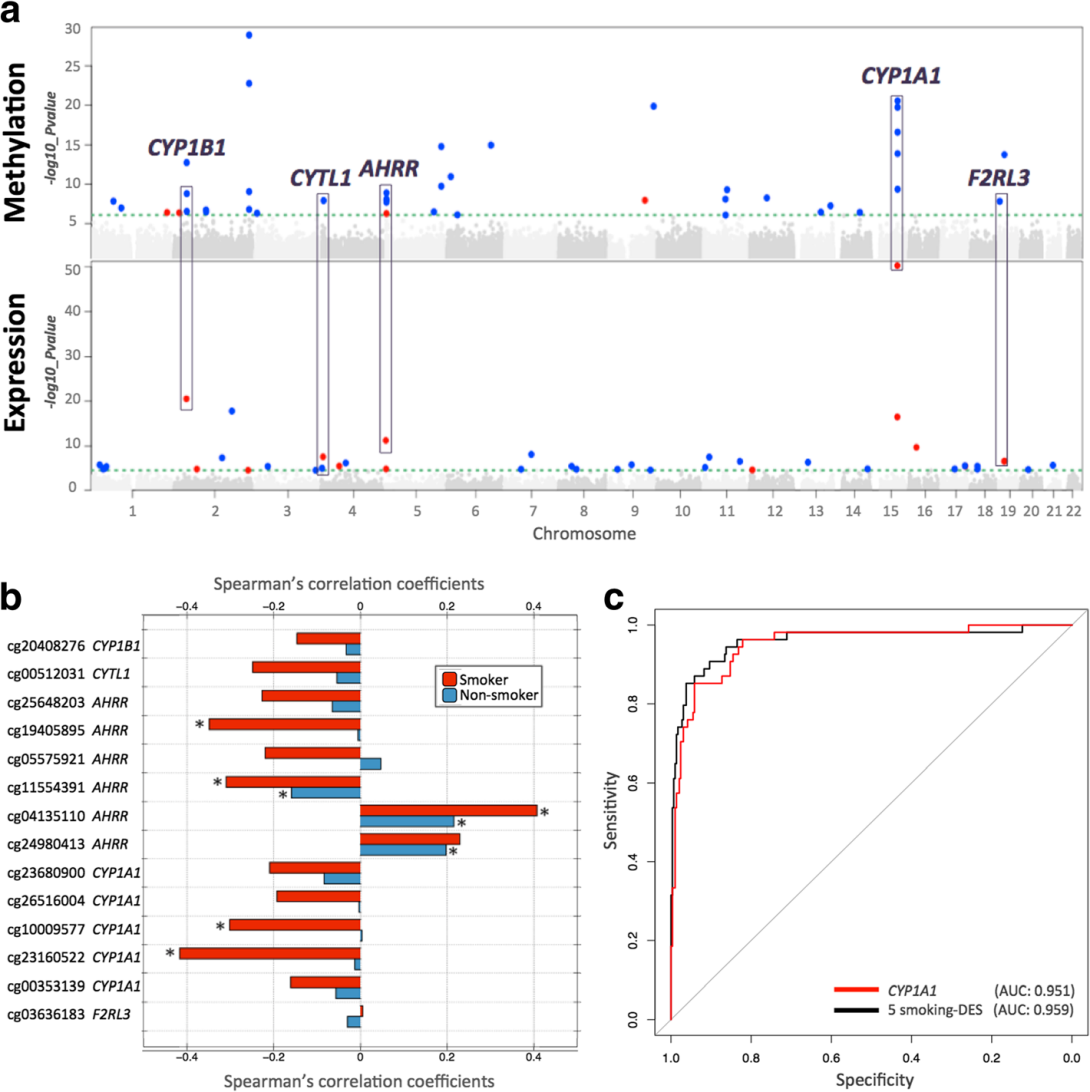
Coordinated smoking-associated DNA methylation and gene expression changes in adipose tissue. **a** Manhattan plots of genome-wide results for methylation (upper panel) and gene expression (lower panel) association with smoking in 345 adipose samples. Smoking-DMS and smoking-DES are indicated above the 1% FDR line (green dashed line) and are classified by direction of effect for current smokers who have higher (red dots) or lower (blue dots) methylation or expression levels compared to non-smokers. Genes highlighted by purple blocks represent five smoking-induced differentially methylated and expressed genes. **b** Methylation–expression correlation at five genes with coordinated smoking-DMS and smoking-DES. Pairwise Spearman’s correlation coefficients between methylation and gene expression levels for 54 current smokers (red bars) and 291 non-smokers (blue bars). Asterisk indicates significance at *P* < 0.05. **c** Discrimination of current and non-smokers using gene expression levels at the five overlapping genes. Receiver operating characteristic (ROC) curves are shown for the following combinations of predictors: *CYP1A1* gene expression level (red) and five smoking-DES (black) in the full dataset as an illustrative example, including AUC values from the full dataset

Our study design is summarized in Fig. 1. Both DNA methylation and gene expression profiles were explored in adipose tissue biopsies from 542 subjects, comprising 54 current smokers, 197 ex-smokers, and 291 non-smokers. The 197 ex-smokers in our sample were excluded from analyses investigating methylation differences between current smokers and non-smokers, but were the focus of subsequent smoking cessation analyses. DNA methylation levels at 467,889 CpG sites from the Illumina Infinium HumanMethylation450 BeadChip were first compared between current smokers (mean BMI = 26.11 ± 4.66, mean age = 54.17 ± 8.31) and non-smokers (mean BMI = 26.95 ± 4.83, mean age = 59.18 ± 9.58). At a false discovery rate of 1% (*P* < 8.37 × 10^−7^), there were 42 smoking differentially methylated signals (smoking-DMS) or CpG sites, and these were located in 29 unique genomic regions comprising of 28 genes and 1 intergenic region (Fig. 2a). Smoking-DMS are located predominantly in the gene body (47.6%), extended promoter region (38.1%), 3′UTR (4.7%), and intergenic region (9.5%), representing an enrichment of signals in the gene body relative to array composition. Using Roadmap annotations (adipose nuclei) [31], we observed that 16 smoking-DMS (38%) were located in enhancers and 9 (21%) were in or near active transcription start sites (TSS). Of these 25 enhancer or TSS signals, 9 were flanking bivalent enhancers (*n* = 3) or TSS (*n* = 6). As expected, methylation levels of current smokers were lower than those in non-smokers in the majority (90.5%) of the 42 signals (Table 1).

**Table 1:** Smoking differentially methylated sites in adipose tissue (42 smoking-DMS)

IlmnID	CHR	Location	Gene name	Non-smoker	Current smoker	Coef.	S.E.	*P* value	*cis*-meQTL	S*
				*β* (mean ± SD)	*β* (mean ± SD)					
cg05951221	2	233284402	*2q37.1*	0.255 ± 0.054	0.172 ± 0.040	− 1.380	0.108	1.28 × 10^−29^	rs2853386; 3.87 × 10^−8^	
cg21566642	2	233284661	*2q37.1*	0.225 ± 0.040	0.167 ± 0.029	− 1.347	0.122	1.87 × 10^−23^		
cg23680900	15	75017924	*CYP1A1*	0.202 ± 0.036	0.155 ± 0.030	− 1.198	0.118	2.96 × 10^−21^		O
cg14120703	9	139416102	*NOTCH1*	0.748 ± 0.045	0.693 ± 0.044	− 1.172	0.118	1.44 × 10^−20^		
cg26516004	15	75019376	*CYP1A1*	0.696 ± 0.047	0.628 ± 0.058	− 1.258	0.126	1.95 × 10^−20^		Y
cg10009577	15	75018150	*CYP1A1*	0.068 ± 0.021	0.050 ± 0.016	− 0.810	0.090	2.48 × 10^−17^		Y
cg01985595	6	136479501	*PDE7B*	0.961 ± 0.025	0.936 ± 0.032	− 1.015	0.119	1.09 × 10^−15^		Y
cg22418620	5	172072885	*NEURL1B*	0.832 ± 0.049	0.765 ± 0.057	− 1.077	0.127	1.63 × 10^−15^	rs57285944; 2.15 × 10^−8^	Y
cg23160522	15	75015787	*CYP1A1*	0.622 ± 0.033	0.583 ± 0.044	− 0.991	0.122	1.33 × 10^−14^		Y
cg03636183	19	17000585	*F2RL3*	0.506 ± 0.040	0.473 ± 0.038	− 0.826	0.103	1.80 × 10^−14^		
cg07992500	2	37896583	*CDC42EP3*	0.771 ± 0.051	0.719 ± 0.052	− 1.087	0.141	1.88 × 10^−13^	rs7595854; 1.32 × 10^−7^	
cg12531611	6	11212619	*NEDD9*	0.909 ± 0.021	0.892 ± 0.024	− 0.855	0.120	1.12 × 10^−11^		O
cg03646542	5	172076155	*NEURL1B*	0.689 ± 0.037	0.654 ± 0.035	− 0.880	0.133	1.87 × 10^−10^	rs7715699; 1.72 × 10^−10^	Y
cg00353139	15	75017914	*CYP1A1*	0.034 ± 0.013	0.022 ± 0.010	− 0.787	0.121	4.47 × 10^−10^	rs11072498; 2.47 × 10^−6^	Y
cg21124714	11	72983097	*P2RY6*	0.736 ± 0.037	0.707 ± 0.033	− 0.874	0.136	5.15 × 10^−10^		Y
cg01940273	2	233284934	*2q37.1*	0.334 ± 0.045	0.302 ± 0.044	− 0.679	0.105	8.93 × 10^−10^		
cg25648203	5	395444	*AHRR*	0.503 ± 0.044	0.459 ± 0.040	− 0.825	0.132	1.30 × 10^−9^		
cg20408276	2	38300586	*CYP1B1*	0.548 ± 0.060	0.499 ± 0.059	− 0.781	0.125	1.61 × 10^−9^		O
cg20131897	12	52305332	*ACVRL1*	0.694 ± 0.034	0.673 ± 0.028	−0.693	0.116	5.61 × 10^−9^	rs1700159; 2.97 × 10^−7^	Y
cg21611682	11	68138269	*LRP5*	0.370 ± 0.041	0.336 ± 0.035	−0.734	0.124	8.10 × 10^−9^		
cg19405895	5	407315	*AHRR*	0.955 ± 0.014	0.942 ± 0.024	−0.768	0.128	8.38 × 10^−9^		Y
cg05575921	5	373378	*AHRR*	0.713 ± 0.044	0.682 ± 0.039	− 0.611	0.104	1.07 × 10^−8^	rs7731963; 3.97 × 10^−8^	
cg13531977	9	112013420	*EPB41L4B*	0.807 ± 0.035	0.833 ± 0.029	0.831	0.140	1.14 × 10^−8^		Y
cg00512031	4	5021976	*CYTL1*	0.880 ± 0.026	0.855 ± 0.028	−0.760	0.129	1.23 × 10^−8^	chr4:5022470;1.42 × 10^−9^	Y
cg25189904	1	68299493	*GNG12*	0.100 ± 0.043	0.064 ± 0.030	− 0.771	0.131	1.48 × 10^−8^		
cg00378510	19	2291020	*LINGO3*	0.217 ± 0.059	0.181 ± 0.053	−0.781	0.134	1.53 × 10^−8^	rs12609156; 6.83 × 10^−18^	
cg11554391	5	321320	*AHRR*	0.065 ± 0.019	0.048 ± 0.014	−0.720	0.125	2.00 × 10^−8^		
cg01802380	13	107865407	*FAM155A*	0.845 ± 0.030	0.825 ± 0.037	−0.737	0.133	5.69 × 10^−8^	rs9520326; 1.52 × 10^−12^	Y
cg14179389	1	92947961	*GFI1*	0.083 ± 0.030	0.063 ± 0.028	−0.665	0.122	1.07 × 10^−7^		
cg06644428	2	233284112	*2q37.1*	0.036 ± 0.018	0.024 ± 0.010	− 0.704	0.130	1.61 × 10^−7^		
cg12081267	2	98486185	*TMEM131*	0.878 ± 0.038	0.858 ± 0.035	− 0.650	0.122	1.97 × 10^−7^		Y
cg02162897	2	38300537	*CYP1B1*	0.567 ± 0.060	0.520 ± 0.061	−0.674	0.127	2.89 × 10^−7^		O
cg11555067	2	99081350	*INPP4A*	0.725 ± 0.047	0.700 ± 0.046	−0.717	0.138	3.18 × 10^−7^	rs3754893; 2.27 × 10^−7^	
cg04134818	5	148998446	*FLJ41603*	0.153 ± 0.026	0.133 ± 0.025	−0.690	0.132	3.26 × 10^−7^	rs11950259; 7.83 × 10^−6^	Y
cg03976650	13	77456505	*KCTD12*	0.667 ± 0.061	0.612 ± 0.067	−0.754	0.143	3.56 × 10^−7^		Y
cg22851561	14	74214183	*C14orf43*	0.422 ± 0.041	0.390 ± 0.040	−0.634	0.121	3.92 × 10^−7^		
cg10376100	1	236017278	*LYST;MIR1537*	0.923 ± 0.036	0.947 ± 0.030	0.615	0.117	4.03 × 10^−7^		Y
cg04063216	2	14772482	*FAM84A*	0.071 ± 0.016	0.075 ± 0.019	0.441	0.085	4.39 × 10^−7^		Y
cg16320419	3	5025570	*BHLHE40*	0.352 ± 0.052	0.315 ± 0.048	− 0.699	0.135	4.88 × 10^−7^		
cg04135110	5	346695	*AHRR*	0.339 ± 0.061	0.384 ± 0.065	0.699	0.137	5.34 × 10^−7^	rs2672748; 3.42 × 10^−17^	
cg20109054	6	31804109	*C6orf48;SNORD52*	0.091 ± 0.026	0.072 ± 0.023	− 0.659	0.130	7.85 × 10^−7^	rs3828922; 2.74 × 10^−5^	
cg16721845	11	68518800	*MTL5*	0.018 ± 0.008	0.014 ± 0.007	− 0.530	0.106	8.37 × 10^−7^		Y

*IlmnID*, Illumina probe ID; *CHR*, chromosome; *Location*, location of the CpG site (bp); *β (mean ± SD)*, mean and standard deviation of the Illumina beta methylation levels in the non-smoker and current smoker group; *Coef.*, regression coefficients from the linear mixed effect model, positive values denote hypermethylation in current smokers and negative values denote hypo-methylation in current smokers; *cis-meQTL*, top significant *cis*-meQTL for the CpG site; S*, adipose tissue-specific effect

Here, we compared our results to one of the biggest smoking-EWAS conducted in blood [20], probes not listed as their significant signals (on their Additional file 2: Table S2, FDR ≤ 0.05) were recorded as “Y” in this table; probes with significant effects in blood in the opposite direction are recorded as “O”

To assess the impact of potential confounders on these results, we performed two follow-up analyses. First, we considered the impact of adipose tissue cell-type composition heterogeneity by also analyzing these data within the reference-free EWAS framework [32]. We observed that the 42 smoking-DMS remained significant at false discovery rate (FDR) of 1%, suggesting that cell composition within adipose tissue did not have a major impact on our findings (Additional file 1: Figure S1). Second, habitual smoking is strongly associated with alcohol consumption [33], and in our data, current smokers and ex-smokers have a higher alcohol intake compared to non-smokers (average alcohol intake = 5.96 (non-smokers), 10.03 (ex-smokers), and 11.67 (current smokers) grams per day, *P* = 1.06 × 10^−5^). Although our smoking analyses take into account alcohol consumption as a covariate, it is possible that the smoking-DMS still in part capture alcohol consumption. To test for the co-occurrence of differentially methylated signals for smoking and alcohol consumption, we performed an alcohol EWAS adjusting for smoking to compare the results with the 42 smoking-DMS. We observed no significant association between alcohol consumption and methylation at genome-wide significance after adjusting for smoking in adipose tissue, and only 7 smoking-DMS in *AHRR* (cg01802380, cg04134818, cg19405895), *CYP1B1* (cg19405895, cg20408276), *FAM84A* (cg04063216), and *C6orf48* (cg20109054) surpassed nominal significance (*P* values between 0.05 and 0.005).

**Fig. 3:**
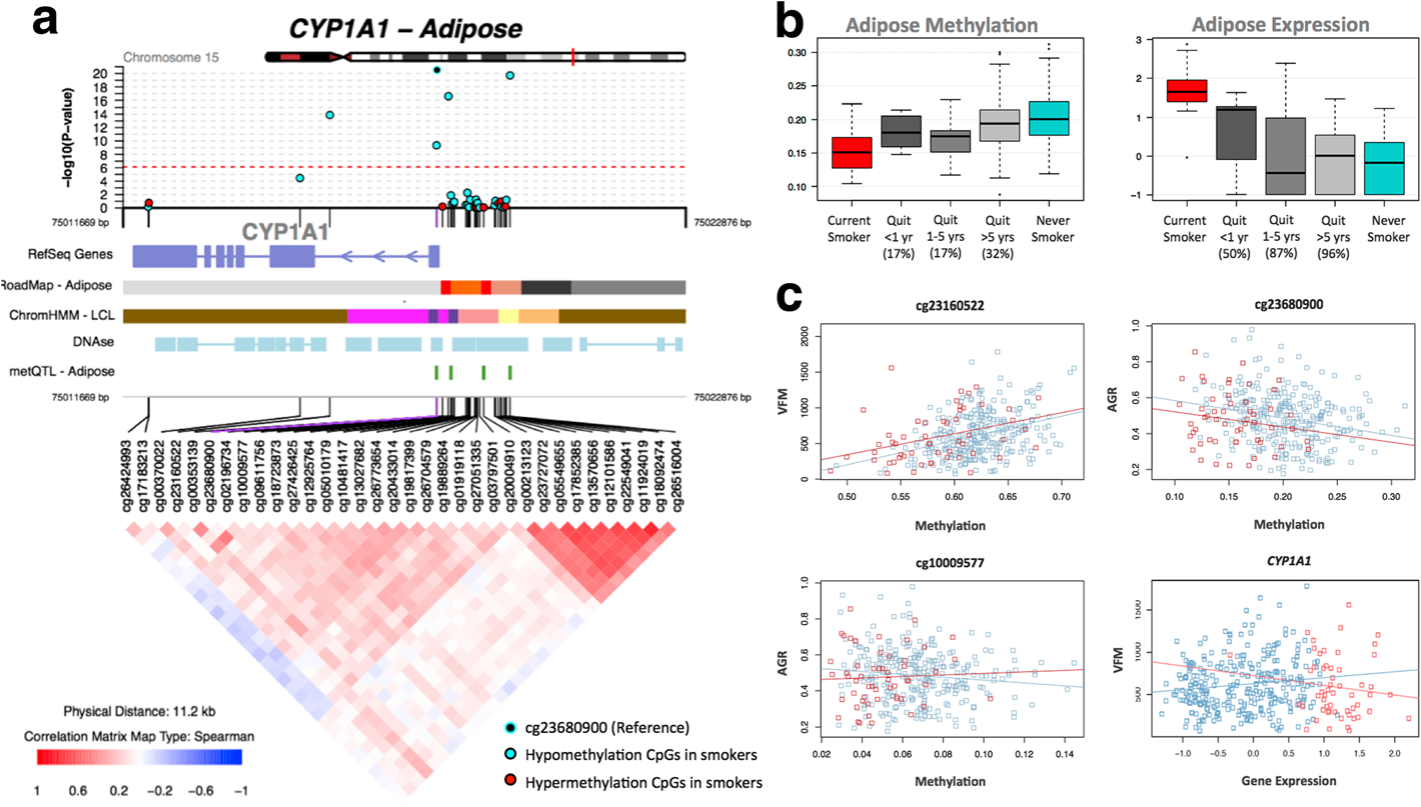
Smoking-associated DNA methylation and gene expression patterns at *CYP1A1.* **a** coMET plot [90] describing the genomic region of epigenome-wide association between smoking and *CYP1A1* methylation (top panel), along with functional annotation of the region (middle panel), and pattern of co-methylation at the 34 CpG sites of *CYP1A1* (bottom panel). **b** DNA methylation and gene expression changes with respect to smoking cessation. Methylation (at cg23680900) and gene expression levels are shown for five smoking status categories: current smokers (red); subjects who quit within 1 year, subjects who quit between 1 and 5 years, and subjects who quit over 5 years at the time of methylation sampling (gray); and non-smokers (blue). *X*-axis labels include the proportion of subjects who reverted in each smoking quit year category. **c** *CYP1A1* methylation associations with adiposity phenotypes, visceral fat mass (VFM), and android-to-gynoid fat ratio (AGR). DNA methylation levels at three CpG sites (cg23160522, cg23680900, and cg10009577 in *CYP1A1*) are shown against adiposity phenotypes in current (red) and non-smokers (blue)

We next compared RNA-sequencing profiles from the same tissue biopsy between current smokers and non-smokers at the gene-based level using RPKM values across 17,399 genes. At an FDR of 1% (*P* < 2.86 × 10^−5^), there were 42 differentially expressed signals (smoking-DES) or genes (Fig. 2a), and 14 of these were upregulated in current smokers (Table 2). The strongest smoking-related expression signal was in the *CYP1A1* gene—a lung cancer susceptibility gene, which was also one of the differentially methylated signals. Gene expression levels in *CYP1A1* were higher in current smokers compared to non-smokers (Figs. 2a and 3).

**Table 2:** Smoking differentially expressed genes in adipose tissue (42 smoking-DES)

ID	CHR	Name	Coef.	S.E.	*P* value	*cis* eQTLs
ENSG00000140465.7	15	*CYP1A1*	1.899	0.103	5.37 × 10^−51^	rs35213055; 1.53 × 10^−6^
ENSG00000138061.7	2	*CYP1B1*	1.373	0.131	2.83 × 10^−21^	
ENSG00000144331.14	2	*ZNF385B*	− 1.257	0.134	1.53 × 10^−18^	rs9288034; 8.33 × 10^−5^
ENSG00000179151.6	15	*EDC3*	1.167	0.129	3.10 × 10^−17^	
ENSG00000063438.12	5	*AHRR*	1.059	0.149	6.03 × 10^−12^	
ENSG00000175267.8	16	*VWA3A*	0.932	0.139	2.18 × 10^−10^	
ENSG00000170381.7	7	*SEMA3E*	− 0.821	0.137	8.35 × 10^−9^	chr7:83264879;1.22 × 10^−10^
ENSG00000170891.6	4	*CYTL1*	0.807	0.142	2.82 × 10^−8^	
ENSG00000187486.5	11	*KCNJ11*	− 0.859	0.148	3.27 × 10^−8^	
ENSG00000168280.11	2	*KIF5C*	− 0.813	0.145	4.74 × 10^−8^	
ENSG00000006016.5	19	*CRLF1*	0.769	0.146	2.53 × 10^−7^	chr19:18717389; 2.63 × 10^−6^
ENSG00000127533.2	19	*F2RL3*	0.782	0.147	2.89 × 10^−7^	
ENSG00000149294.11	11	*NCAM1*	− 0.715	0.135	3.03 × 10^−7^	rs17510563; 2.01 × 10^−7^
ENSG00000120693.9	13	*SMAD9*	− 0.733	0.140	4.76 × 10^−7^	
ENSG00000169116.7	4	*PARM1*	− 0.686	0.133	6.76 × 10^−7^	
ENSG00000154330.6	9	*PGM5*	− 0.716	0.147	1.72 × 10^−6^	
ENSG00000162430.12	1	*SEPN1*	− 0.663	0.137	1.82 × 10^−6^	
ENSG00000154721.9	21	*JAM2*	− 0.667	0.136	2.23 × 10^−6^	
ENSG00000177303.4	17	*CASKIN2*	− 0.669	0.140	2.90 × 10^−6^	
ENSG00000157404.10	4	*KIT*	0.708	0.150	3.31 × 10^−6^	
ENSG00000161544.4	17	*CYGB*	0.621	0.131	3.42 × 10^−6^	
ENSG00000154065.9	18	*ANKRD29*	− 0.684	0.144	3.49 × 10^−6^	
ENSG00000176907.3	8	*C8orf4*	− 0.714	0.151	3.56 × 10^−6^	
ENSG00000168032.4	3	*ENTPD3*	− 0.674	0.140	3.86 × 10^−6^	rs34158576; 7.60 × 10^−6^
ENSG00000162367.6	1	*TAL1*	− 0.665	0.142	4.17 × 10^−6^	
ENSG00000180785.8	11	*OR51E1*	− 0.655	0.142	6.82 × 10^−6^	rs11033126; 3.78 × 10^−10^
ENSG00000164010.9	1	*ERMAP*	− 0.690	0.154	9.50 × 10^−6^	
ENSG00000068078.12	4	*FGFR3*	− 0.643	0.143	9.68 × 10^−6^	rs744658; 9.68 × 10^−8^
ENSG00000246223.4	14	*C14orf64*	− 0.633	0.142	1.44 × 10^−5^	rs75700090; 2.00 × 10^−5^
ENSG00000145506.9	5	*NKD2*	0.616	0.140	1.46 × 10^−5^	
ENSG00000161649.7	17	*CD300LG*	− 0.648	0.147	1.48 × 10^−5^	
ENSG00000163873.5	1	*GRIK3*	− 0.643	0.146	1.50 × 10^−5^	
ENSG00000053747.9	18	*LAMA3*	− 0.652	0.148	1.57 × 10^−5^	
ENSG00000183733.6	2	*FIGLA*	0.406	0.093	1.57 × 10^−5^	
ENSG00000164736.5	8	*SOX17*	− 0.629	0.144	1.64 × 10^−5^	
ENSG00000106078.12	7	*COBL*	− 0.680	0.155	1.65 × 10^−5^	
ENSG00000120156.14	9	*TEK*	− 0.610	0.140	1.67 × 10^−5^	
ENSG00000178726.5	20	*THBD*	− 0.612	0.141	2.00 × 10^−5^	
ENSG00000177675.4	12	*CD163L1*	0.635	0.148	2.40 × 10^−5^	
ENSG00000136828.13	9	*RALPGS1*	− 0.646	0.151	2.60 × 10^−5^	
ENSG00000135914.4	2	*HTR2B*	0.613	0.144	2.82 × 10^−5^	
ENSG00000090530.5	3	*LEPREL1*	− 0.617	0.145	2.86 × 10^−5^	rs6768989; 1.10 × 10^−9^

*ID*, Ensemble ID; *CHR*, chromosome; *Coef.*, regression coefficients from the linear mixed effect model, positive values reflect higher expression in current smokers and negative values represent lower expression in current smokers; *eQTL*, expression quantitative trait locus

Comparison of the FDR 1% genome-wide significant smoking-DMS and smoking-DES showed overlapping signals at five genes comprising 14 CpG sites, and these included *AHRR*, *CYP1A1*, *CYP1B1*, *CYTL1*, and *F2RL3* (Fig. 2a). CpG sites within *AHRR*, *CYP1B1*, and *F2RL3* were located in the gene body, whereas CpG sites in or near *CYP1A1* and *CYTL1* were located 200 kb to 1500 kb away from the transcription start sites. All five genes were upregulated in current smokers, and in the majority of smoking-DMS (93%), current smokers showed lower methylation levels compared to non-smokers. These predominantly negative correlations between methylation and expression at these five genes suggested regulatory effects (Table 3, Fig. 2b). The methylation-expression correlations at some of these CpG sites were only observed in current smokers, and overall correlations were stronger in smokers compared to non-smokers.

**Table 3:** Correlation between DNA methylation and gene expression

Gene name	IlmnID	CHR	Location	ID	*r*	*P* value
*CYP1B1*	cg20408276	2	38300586	ENSG00000138061.7	− 0.171	1.39 × 10^−3^
*CYTL1*	cg00512031	4	5021976	ENSG00000170891.6	− 0.176	1.03 × 10^−3^
*AHRR*	cg25648203	5	395444	ENSG00000063438.12	− 0.167	1.80 × 10^−3^
*AHRR*	cg19405895	5	407315	ENSG00000063438.12	− 0.134	1.29 × 10^−2^
*AHRR*	cg05575921	5	373378	ENSG00000063438.12	− 0.060	0.2633
*AHRR*	cg11554391	5	321320	ENSG00000063438.12	− 0.216	5.37 × 10^−5^
*AHRR*	cg04135110	5	346695	ENSG00000063438.12	0.279	1.31 × 10^−7^
*AHRR*	cg24980413	5	346987	ENSG00000063438.12	0.252	2.10 × 10^−6^
*CYP1A1*	cg23680900	15	75017924	ENSG00000140465.7	− 0.329	3.94 × 10^−10^
*CYP1A1*	cg26516004	15	75019376	ENSG00000140465.7	− 0.298	1.70 × 10^−8^
*CYP1A1*	cg10009577	15	75018150	ENSG00000140465.7	-0.266	5.22×10^-7^
*CYP1A1*	cg23160522	15	75015787	ENSG00000140465.7	− 0.299	1.48 × 10^−8^
*CYP1A1*	cg00353139	15	75017914	ENSG00000140465.7	− 0.222	3.22 × 10^−5^
*F2RL3*	cg03636183	19	17000585	ENSG00000127533.2	− 0.130	0.0159

*IlmnID*, Illumina probe ID; *CHR*, chromosome; *Location*, Illumina probe location (bp); *ID*, Ensemble ID; *r*, Spearman’s correlation coefficients between methylation and gene expression data (*n* = 345)

### Prediction of smoking status based on DNA methylation and gene expression

To assess the impact of smoking on DNA methylation and gene expression within the same analysis framework and at a comparable scale, we used methylation and expression changes at these five overlapping genes (14 CpG sites) to predict a subject’s smoking status using a logistic regression model. We split the overall dataset into training and validation sets of equal size and report here the average area under curve (AUC) values from 1000 validation sets. The combination of 14 smoking-DMS levels and 5 smoking-DES levels resulted in reasonable discrimination of smoking status (AUC: 0.865). Compared to the prediction results based on 14 smoking-DMS levels alone (AUC: 0.888), smoking-DES levels are better predictors (all five genes, AUC: 0.951). This suggests that smoking leaves a greater impact on gene expression levels, compared to DNA methylation levels at these overlapping genes. A similar high predictive value can be achieved by using gene expression levels at just a single gene, *CYP1A1* (AUC: 0.952) (Fig. 2c). *CYP1A1* was the peak smoking differentially expressed gene, with differentially methylated signals in the promoter, and a negative correlation between methylation and expression (Fig. 3b).

### Adipose-specific and tissue-shared smoking signals

**Fig. 4:**
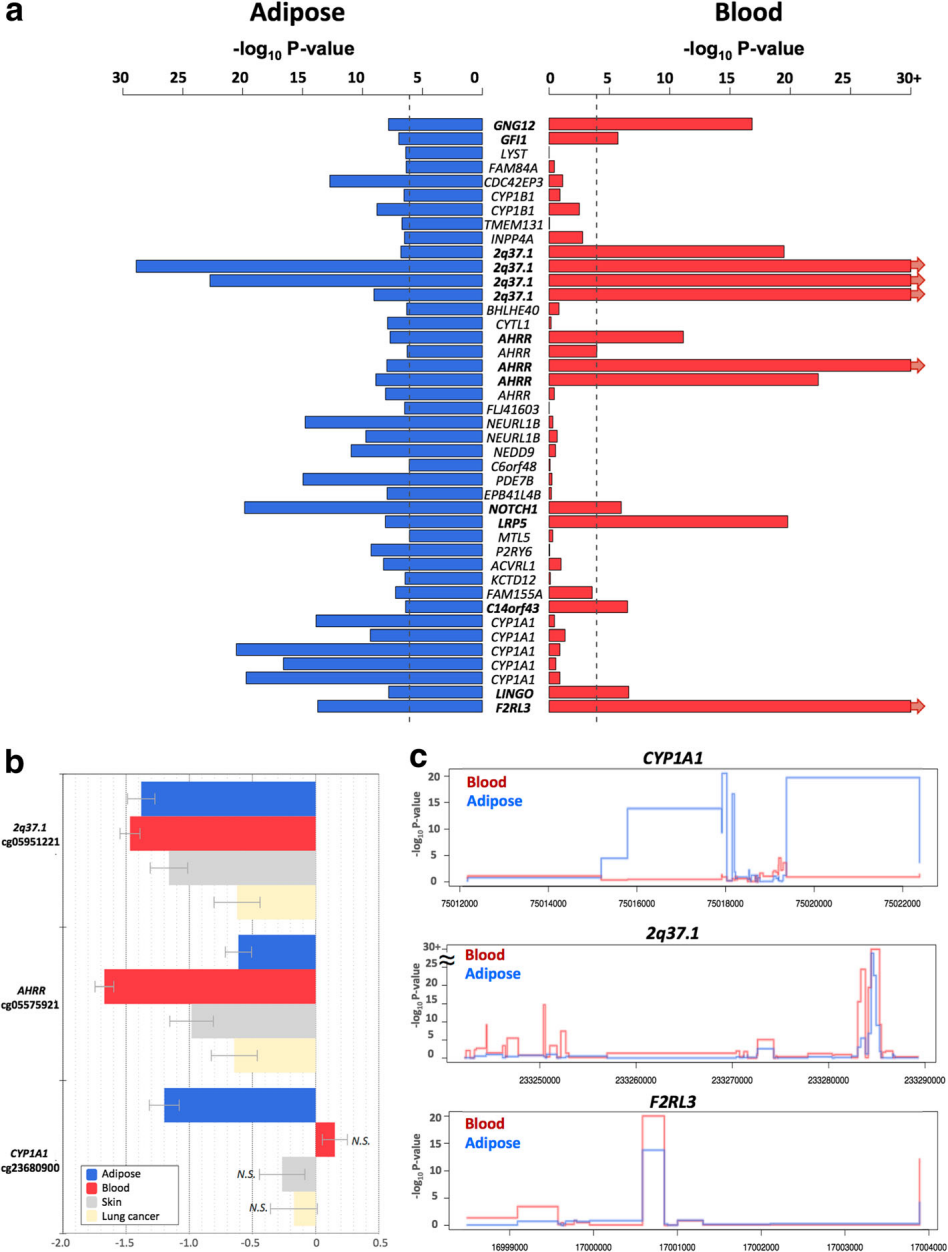
Tissue-shared and adipose-specific smoking signals. **a** Tissue-shared DNA methylation effects across adipose tissue and whole blood. The bar-plot shows the -log_10_ *P* value of the 42 smoking-DMS in adipose samples (blue), and the corresponding *P* value in the blood samples (red bars). Gene names in bold denote significantly associated genes in both tissues. **b** Tissue-shared and tissue-specific DNA methylation effects for adipose tissue, whole blood, skin, and lung cancer tissue at 2q37.1, *AHRR*, and *CYP1A1*. Each bar represents the coefficient estimate from smoking-EWAS with standard error bars. Positive values indicate a hypermethylation in current smokers. Colors reflect tissues, with coefficients in adipose (blue), blood (red), skin (gray), and lung tissue (yellow). N.S. indicates non-significance. **c** Examples of smoking effects that are tissue-shared and tissue-specific across adipose (blue) and blood (red) samples in our datasets, including adipose-specific (*CYP1A1* in our dataset) and tissue-shared (*2q37.1* and *F2RL3*) smoking-DMS

**Fig. 5:**
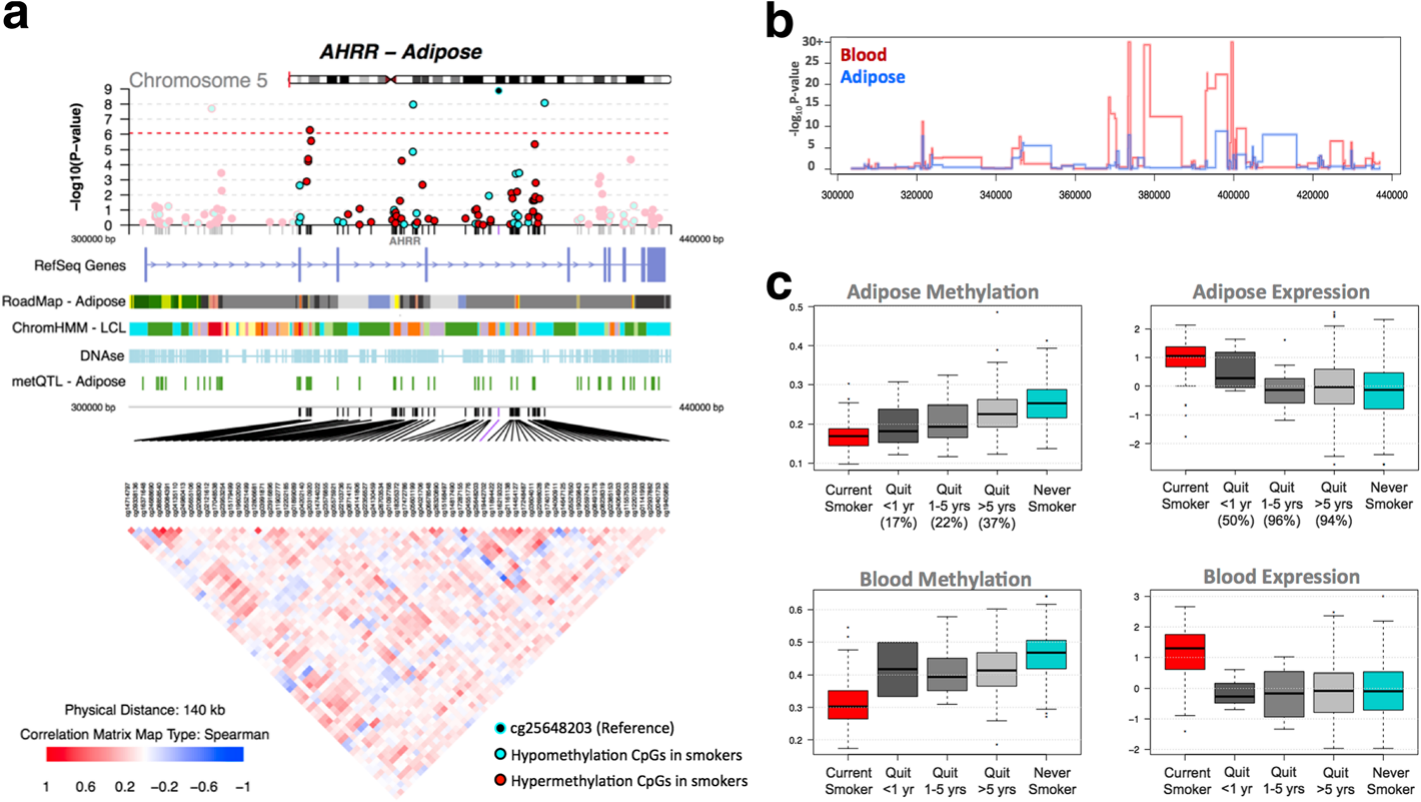
Tissue-shared smoking-associated DNA methylation and gene expression patterns at *AHRR*. **a** coMET plot [49] of the association between 66 *AHRR* CpG sites and smoking. Top panel shows the -log_10_*P* value of the association; the middle panel shows genomic annotation; and the lower panel shows co-methylation patterns based on Spearman correlation coefficients. **b** Tissue-shared and tissue-specific methylation signals across CpG sites in the *AHRR* gene region in adipose (blue) and blood samples (red). **c** DNA methylation and gene expression levels with respect to smoking cessation. Methylation and gene expression levels are shown for five different smoking status categories: current smokers (red); subjects who quit within 1 year, subjects who quit between 1 and 5 years, and subjects who quit over 5 years at the time of methylation sampling (gray); and non-smokers (blue). *X*-axis labels include the proportion of subjects who reverted in each smoking quit year category

To test if the effects of smoking are shared across tissues, we first compared our adipose findings to results from whole blood samples. To this end, we tested for association between smoking and whole blood genome-wide DNA methylation (in 569 individuals) and gene expression profiles (in 237 individuals), comparing current smokers with non-smokers. In blood, genome-wide significant results at FDR 1% for smoking DMS and DES overlapped at four genes (Additional file 2: Table S1). Altogether, comparison of FDR 1% significant smoking-DMS results across the adipose and whole blood datasets identified 14 CpG sites that were genome-wide differentially methylated in both blood and adipose tissue (Fig. 4a). The 14 tissue-shared CpG sites fell in eight genes, including *GNG12*, *GFI1*, *AHRR*, *NOTCH1*, *LRP5*, *C14orf43*, *LINGO3*, *F2RL3*, and in the 2q37.1 intergenic region (Table 4). All of these sites were previously reported as smoking differentially methylated sites in blood in previous studies [6–19] and include *AHRR*—the most robustly replicated smoking-methylation signal (Fig. 5a). DNA methylation changes in two genes (*AHRR* and *F2RL3*) that exhibit both expression and methylation smoking-associated effects in adipose tissue were also present in the blood (Figs. 4c and 5b).

**Table 4:** Tissue-shared smoking-induced differentially methylated sites in cancer-free subjects

IlmnID	CHR	Location	Gene name	Adipose tissue (*n* = 345)		Blood samples (*n* = 567)		Skin tissue (*n* = 195)	
				Coef.	*P* value	Coef.	*P* value	Coef.	*P* value
cg25189904	1	68299493	*GNG12*	− 0.771	1.48 × 10^−8^	− 0.974	6.92 × 10^−18^	− 0.434	1.58 × 10^−2^
cg14179389	1	92947961	*GFI1*	− 0.665	1.07 × 10^−7^	− 0.404	4.74 × 10^−6^	− 0.408	1.89 × 10^−2^
cg06644428	2	233284112	2q37.1	− 0.704	1.61 × 10^−7^	− 0.864	1.76 × 10^−19^	− 0.641	3.39 × 10^−4^
cg05951221	2	233284402	2q37.1	− 1.38	1.28 × 10^−29^	− 1.471	3.65 × 10^−60^	− 1.161	6.13 × 10^−13^
cg21566642	2	233284661	2q37.1	− 1.347	1.87 × 10^−23^	− 1.491	9.67 × 10^−61^	− 1.138	4.83 × 10^−11^
cg01940273	2	233284934	2q37.1	− 0.679	8.93 × 10^−10^	− 1.415	3.17 × 10^−52^	− 0.302	3.09 × 10^−2^
cg11554391	5	321320	*AHRR*	− 0.72	2.00 × 10^−8^	− 0.694	8.10 × 10^−12^	− 0.494	4.91 × 10^−3^
cg05575921	5	373378	*AHRR*	− 0.611	1.07 × 10^−8^	− 1.672	2.45 × 10^− 80^	− 0.982	7.24 × 10^−8^
cg25648203	5	395444	*AHRR*	− 0.825	1.30 × 10^−9^	− 0.937	3.50 × 10^−22^	− 0.398	7.29 × 10^−3^
cg14120703	9	139416102	*NOTCH1*	− 1.172	1.44 × 10^−20^	− 0.352	1.84 × 10^−6^	− 0.423	8.69 × 10^−3^
cg21611682	11	68138269	*LRP5*	− 0.734	8.10 × 10^−9^	− 0.874	4.23 × 10^−20^	0.075	6.38 × 10^−1^
cg22851561	14	74214183	*C14orf43*	− 0.634	3.92 × 10^−7^	− 0.5	5.24 × 10^−7^	− 0.326	7.07 × 10^−2^
cg00378510	19	2291020	*LINGO3*	− 0.781	1.53 × 10^−8^	− 1.478	3.59 × 10^−62^	− 0.133	4.66 × 10^−1^
cg03636183	19	17000585	*F2RL3*	− 0.826	1.80 × 10^−14^	− 0.466	2.37 × 10^−7^	− 0.372	1.45 × 10^−2^

*IlmnID*, Illumina probe ID; *CHR*, chromosome; *Location*, Illumina probe location (bp); *Coef.*, regression coefficients from the linear mixed effect model, positive values denote hypermethylation in non-smokers and negative values denote hypermethylation in current smokers

We sought to explore the observed tissue-shared methylation effects at the 14 putative tissue-shared CpG sites in additional datasets including 195 skin tissue samples from healthy subjects [34] and 168 lung tissue samples from subjects affected with lung cancer. Four of the 14 CpG sites validated in the skin in the intergenic region 2q37.1 (cg05951221, cg06644428, and cg21566642) and in *AHRR* (cg05575921). Furthermore, the majority (*n* = 13) of the 14 tissue-shared CpG sites had lower methylation levels in current smokers compared to non-smokers in both lung and skin methylation datasets, indicating a consistent direction of effect, which was not nominally significant (Table 4, Additional file 2: Table S2). In lung tissue from subjects affected with lung cancer, we validated 3 of the 14 CpG sites in the intergenic region 2q37.1 (cg21566642 and cg05951221) and in the *AHRR* gene (cg05575921) at a Bonferroni-corrected *P* value of 3.57 × 10^−3^ (Additional file 2: Table S2). The smoking-DMS effects observed across tissues were similar for CpG sites in the 2q37.1 region, while the smoking effect was much greater in blood at cg05575921 in *AHRR* (see Table 4, Fig. 4b).

In contrast to the methylation results, gene expression signals showed minimal evidence for tissue-shared impacts. Comparing our FDR 1% genome-wide smoking-DES across adipose and blood datasets showed that only *AHRR* was significantly upregulated in current smokers across both tissues (Fig. 5c). *AHRR* was the only signal that showed both differential methylation and expression changes across all of the datasets that we explored in this study, including blood, adipose, skin, and lung tissue.

A proportion of our smoking-DMS and most of our smoking-DES results appear to be adipose-specific. However, the sample size of the datasets used to explore tissue specificity in gene expression was much lower compared to that used for methylation; therefore, power to detect tissue-shared effects differs across the data types. Furthermore, we are limited by access to available multi-tissue datasets for follow-up, and further investigation of published findings reveals that some of our smoking adipose-specific signals have previously been detected in other tissues [20] For example, one of our peak results at *CYP1A1* showed methylation changes only in adipose tissue and not in the blood (Fig. 4), but has previously been reported as a smoking-methylation signal in blood [20], lung tissue [30, 35], cord blood [36], and placenta [37, 38]. Unlike the persistent tissue-shared effects identified in other smoking-DMS such as signals in *AHRR* and 2q37.1, we found that current smokers have lower *CYP1A1* methylation levels in adipose, skin, and lung tissue, but not in blood [20], placenta, and cord blood samples [36], overall suggesting that smoking may have contrasting effects, resulting in hyper- or hypo-methylation in different tissues (Fig. 4b). A similar contrast in direction of smoking methylation effects is observed at smoking-DMS in *NEDD9* and *CYP1B1* across adipose tissue and in blood (Table 1).

### Replication of adipose smoking methylation signals

We pursued replication of the adipose tissue smoking-DMS in an independent dataset of 104 participants from the LEAP cohort, within the New England Family Study (mean BMI 30.9 ± 7.03, mean age 47 ± 1.7, 48% male), described in detail elsewhere [39]. These individuals were not affected with common diseases and had available adipose biopsy methylation profiles for 46 current smokers and 58 non-smokers. We found that the smoking-methylation direction of association was consistent at all 42 adipose smoking-DMS (Additional file 2: Table S3), and 25 of these also surpassed nominal significance in the replication dataset (*P* = 0.05). At a more stringent threshold, the replication signal was significant at 13 sites, surpassing Bonferroni-adjusted *P* value for the replication analysis (*P* = 1.19 × 10^−3^).

### Signatures of smoking cessation

We next assessed the effect of smoking cessation on the observed adipose DNA methylation and gene expression signals in ex-smokers from the discovery cohort. We considered reversal of smoking methylation or expression signals, that is, the longitudinal change in methylation to reach levels observed in non-smokers. We quantified the number of subjects who reverted to 25% of the change in methylation towards non-smokers, and estimated the proportion of subjects who reverted over time (in smoking-quit years), using the same approach in gene expression (see the “Methods” section).

We explored reversal patterns in adipose tissue at both the 42 smoking-DMS (Additional file 1: Figure S2) and 42 smoking-DES (Additional file 1: Figure S3) and focused on the five differentially methylated and expressed genes (14 CpG sites), where the average number of smoking-quit years was 24.8 (± 13.21) years among 197 ex-smokers. Overall, a rapid rate of reversal was observed in the first 10 years after smoking cessation, after which only subtle changes were detected in both methylation and gene expression. In the expression adipose data, ex-smokers showed a > 50% reversal rate 1 year after smoking cessation and reached > 85% reversal after 10 years (Additional file 1: Figure S3). In comparison, slower reversal was observed in the methylation dataset (Additional file 1: Figure S2). Among the 14 CpG sites, only three (two at *AHRR* and one at *CYP1A1*) showed a 50% reversal rate 1 year after cessation, while the remaining signals showed between 17 and 33% reversal (Figs. 3b and 5c, Additional file 1: Figure S3). Even after > 40 years of smoking cessation, a proportion of smoking-DMS (*n* = 12; 29%) showed less than 40% reversal (Additional file 1: Figure S3). This suggests that smoking leaves a longer lasting influence on DNA methylation levels than on gene expression levels after smoking cessation.

### Controlling for genetic variation

Previous studies have shown heritable impacts on smoking behavior and nicotine addiction [40–43]. We explored the impact of genetic variation on the identified smoking methylation signals. Of the 42 smoking-DMS, 14 CpG sites had genome-wide significant meQTLs in *cis* in adipose tissue (Table 1). Of the 14 tissue-shared smoking-DMS, two signals in 2q37.1 and one in *LINGO3* had meQTLs in *cis* in adipose tissue, and three signals in *AHRR* and one in *F2RL3* had meQTLs in *cis* in blood samples.

Given our observed genetic influences on smoking-DMS, we asked if previously reported genetic variants associated with smoking behavior [42] or nicotine metabolism [43] could impact DNA methylation levels in adipose tissue. We first focused on common genetic variants that were previously associated with smoking phenotypes in the largest smoking genetic association study to date (*n* = 15,907) [42]. We observed that all genetic variants previously strongly linked to smoking behavior (14 SNPs) [42] had an impact on adipose DNA methylation levels in *cis* (Additional file 2: Table S4). We then explored a recently reported association between a cluster of SNPs on chromosome 19 and nicotine metabolism, where the same genetic variants were also associated with blood DNA methylation levels in the same region as meQTLs [43]. We replicate the chromosome 19 meQTL findings in our adipose DNA methylation data at CpGs in genes *CYP2A7*, *ENGL2*, and *LTBP4* (Additional file 2: Table S5), suggesting that these are strong genetic impacts on DNA methylation that are shared across tissues. Taken together, these genetic-methylation association results provide additional support for the hypothesis that some of the observed genetic impacts on smoking behavior and nicotine metabolism may be mediated by DNA methylation.

### Impacts on metabolic health and disease risk

Given the wide-ranging effects of smoking on human disease, we explored the links between the identified adipose methylation and expression smoking signals and phenotypes that are major risk factors for metabolic disease. Three metabolic disease risk phenotypes—total fat mass (TFM), visceral fat mass (VFM), and android-to-gynoid fat ratio (AGR)—were profiled using dual X-ray absorptiometry in 288 subjects with adipose methylation and expression profiles. We assessed the association of the 42 smoking-DMS and 42 smoking-DES with these adiposity phenotypes using a twofold approach.

First, we tested for association between adipose methylation levels at the 42 smoking-DMS and the three phenotypes, adjusting for covariates including age, BMI, and smoking. We observed that smoking-DMS in *CYP1A1* and *NOTCH1* were significantly associated with measures of metabolic disease risk. First, methylation levels at three CpG sites in *CYP1A1* were significantly associated with VFM and AGR, either as main effects (cg23160522 and VFM, beta = 1.35 × 10^−3^, SE = 3.03 × 10^−3^, *P* = 4.35 × 10^−7^; cg23680900 and AGR, beta = − 1.59, SE = 0.44, *P* = 6.58 × 10^−6^) or taking into account interactions (cg10009577 and AGR, *P* = 5.50 × 10^−4^), where current smokers and non-smokers have different patterns of association between DNA methylation at *CYP1A1* cg10009577 and AGR (Fig. 3c). Probe cg10009577 is located in the *CYP1A1* promoter, suggesting gene regulatory impacts on *CYP1A1* expression levels. Correspondingly, we observed a nominally significant association between *CYP1A1* gene expression and VFM (Fig. 3c), where current smokers and non-smokers have different patterns of association (*P* = 0.042). A significant negative association between DNA methylation levels and AGR was also observed with cg14120703 in *NOTCH1* (beta = − 1.80, SE = 0.43, *P* = 1.07 × 10^−7^). We pursued replication of these associations in an independent sample of 69 younger Finnish twins with adipose tissue Illumina 450K methylation profiles. We replicated the overall negative association between *CYP1A1* cg10009577 and AGR (discovery sample beta = − 0.95, SE = 0.31; replication sample beta = − 0.58, SE = 0.25, *P* = 0.02) and observed a similar direction of interaction effects, which did not reach nominal significance in the replication sample (Additional file 2: Table S5).

We performed similar analyses with the 42 smoking-DES and observed main effects at *F2RL3* on the three phenotypes (VFM beta = − 1.5 × 10^−3^, SE = 3.78 × 10^−4^, *P* = 7.8 × 10^−4^; AGR beta = 2.3, SE = 0.56, *P* = 4.5 × 10^−5^; TFM beta = 1.6 × 10^−3^, SE = 3.9 × 10^−4^, *P* = 5.8 × 10^−5^), and *OR51E1* on VFM (beta = − 1.5 × 10^−3^, SE = 3.78 × 10^−4^, *P* = 7.8 × 10^−4^) and AGR (beta = − 2.85, SE = 0.51, *P* = 3.1 × 10^−8^). We did not observe significant evidence for interaction effects in the gene expression results.

**Fig. 6:**
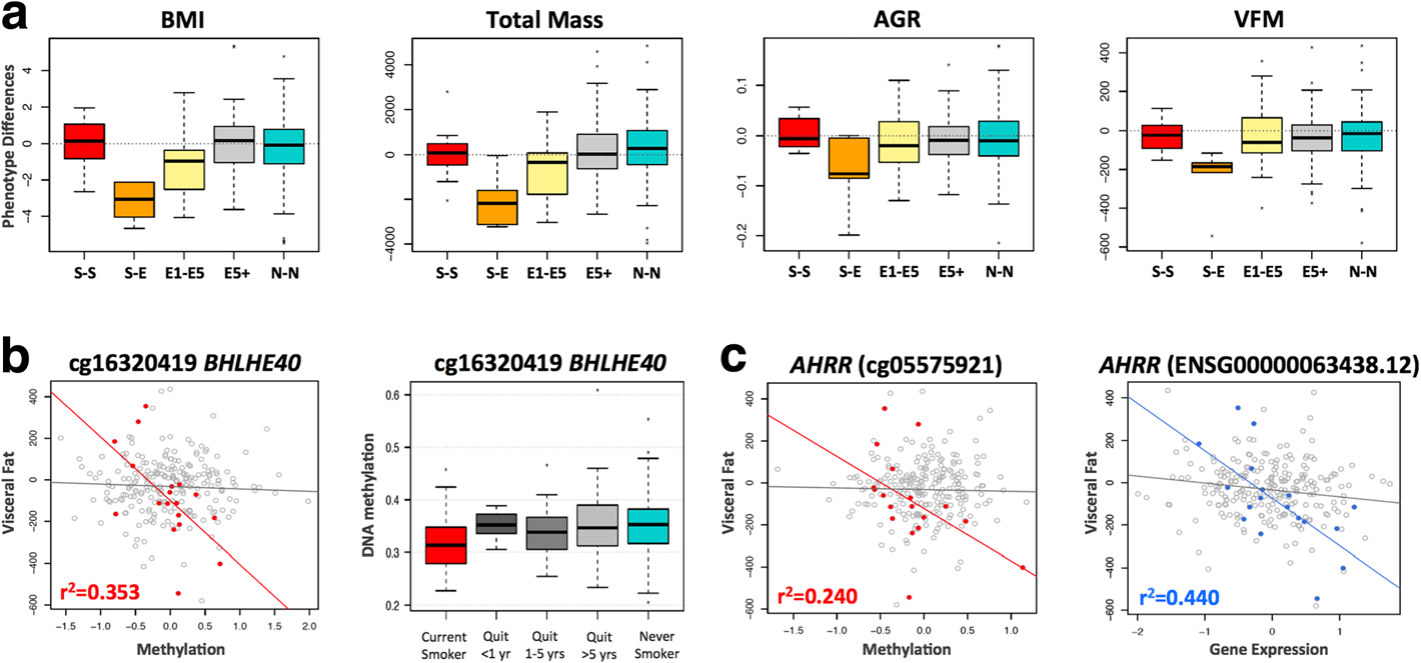
Smoking-DMS and smoking-DES relate to future changes in visceral fat mass on smoking cessation. **a** Adiposity phenotype changes over a 5-year time period between time point 1 (2007–2008) and time point 2 (2012–2013). Adiposity phenotypes include BMI, total fat mass (TFM), android-to-gynoid fat ratio (AGR), and visceral fat mass (VFM). Phenotype changes are shown for five categories of subjects: current smokers at the two time points (S-S, *n* = 12), current smokers at time point 1 who quit smoking by time point 2 (S-E, *n* = 5), ex-smokers (who quit smoking within 1–5 year) at time point 1 who remain ex-smokers at time point 2 (E1-E5, *n* = 13), ex-smokers who quit > 5 years at time point 1 who remain ex-smokers at time point 2 (E5+, *n* = 92), and non-smokers at both time points (N-N, *n* = 124). **b** Left panel shows the association between DNA methylation levels at cg16320419 in *BHLHE40* and future changes in visceral fat mass in 18 subjects in categories S-E and E1-E5 (red points), compared to all remaining subjects (gray points). Right panel shows methylation cessation patterns at cg16320419 in *BHLHE40*. **c** Association between DNA methylation (left panel, red points) and gene expression (right panel, blue points) in *AHRR* with future changes in visceral fat mass in 18 subjects in categories S-E and E1-E5, compared to all remaining subjects (gray points)

In the second set of phenotypic analyses, we explored the role of the 42 smoking-DMS and 42 smoking-DES on weight gain after smoking cessation. Recent studies have reported not only a gain in weight on smoking cessation, but also an increase in visceral fat [5]. We considered adiposity phenotypes in 246 of the individuals in our study at two time points, where time point 1 was the initial adipose DNA methylation profiling and phenotype measurement, and time point 2 was a phenotype measurement on average 5 years later. We found that current smokers who go on to quit smoking over this 5-year interval show a gain in adiposity across all phenotypes (Fig. 6a), and this effect is also observed in individuals who quit within up to 4 years at time point 1. However, our data suggests that this gain in adiposity is not long lasting, because we do not observe this effect in the group of ex-smokers who had quit for > 5 years at time point 1. In comparison, there were no major phenotype changes within constant smokers (current smokers at both time points) or never smokers (non-smokers at both time points) across the two time points.

We tested if the 42 smoking-DMS and 42 DES in adipose tissue could predict future changes in adiposity upon smoking cessation, focusing on visceral fat accumulation as the major risk factor for the development of adiposity-related metabolic diseases. Based on the phenotype results (Fig. 6a), we compared two groups of individuals: first, the combined group (*n* = 18) of current smokers at the time of methylation profiling (time point 1) who subsequently quit smoking (*n* = 5), and individuals who had quit within 1–4 years at time point 1 (*n* = 13); and second, the combined group (*n* = 228) of ex-smokers who had quit for > 5 years at time point 1 (*n* = 92), as well as constant smokers (*n* = 12) and never smokers (*n* = 124) across the two time points. We assessed the impact of methylation or expression at the 42 smoking-DMS (Additional file 1: Figure S4) and 42 smoking-DES (Additional file 1: Figure S5) on future changes in visceral fat, selecting results that showed significantly different patterns of association in the two groups of 18 and 228 subjects.

After Bonferroni correction for multiple testing, we found one DMS and one DES significantly associated with future changes in visceral fat, where a strong association effect was only observed in the group 18 subjects. This group consists of current smokers who go on to quit smoking (*n* = 5) and recent ex-smokers who remain ex-smokers (*n* = 13), and where all subjects exhibit a gain in adiposity over time. The first signal was observed in cg16320419 in *BHLHE40* (methylation by group interaction term *P* = 9.3 × 10^−4^), where methylation levels in current smokers or recent ex-smokers explain 35.5% of the variation in future gain in visceral fat (Fig. 6b). The second signal was observed in *AHRR* (gene expression by group interaction term *P* = 4.7 × 10^−5^), where gene expression levels in current smokers or recent ex-smokers explain 44% of the variation in future gain in visceral fat (Fig. 6c). The results were similar after correcting for smoking years and years since smoking cessation.

## Discussion

Tobacco smoking is a major disease risk factor. Our study is the first to identify smoking-associated DNA methylation and gene expression changes in adipose tissue in humans. Approximately 30% of the identified smoking-methylation signals showed significant coordinated changes in gene expression levels in five genes, giving insights into the cascade of molecular events that are triggered in response to smoking, toxin exposure, and nicotine metabolism. At least a third of smoking-methylation signals (in nine genomic regions) were shared across tissues, showing that smoking leaves tissue-shared signatures. Given that our target tissue was adipose, we considered the impact of the identified smoking methylation and expression signals on metabolic disease risk. Significant associations were observed between visceral fat and android-to-gynoid fat ratio and several smoking-methylation and expression markers. Furthermore, methylation and expression levels at *BHLHE40* and *AHRR* in current smokers or recent ex-smokers were predictive of future gain in visceral fat observed after smoking cessation. Our findings provide a first comprehensive assessment of methylation and expression changes related to smoking in adipose tissue, with insights for metabolic health and disease risk.

Coordinated smoking methylation and expression changes overlapped at five genes (*AHRR*, *CYP1A1*, *CYP1B1*, *CYTL1*, and *F2RL3*), which include well-known and strongly replicated smoking-methylation signals, such as *AHRR* and *F2RL3*. Some of these genes have previously been linked to human phenotypes. For example, GWAS associations have been reported with multiple diseases and traits, such as drinking behavior (*CYTL1*) [44], cystic fibrosis severity (*AHRR*) [45], caffeine consumption (*CYP1A1*) [46], and diastolic blood pressure (*CYP1A1*) [47], and methylation levels at *AHRR* have been linked to multiple phenotypes including lung function [48] and BMI [49]. At the five overlapping genes, methylation levels were predominantly negatively correlated with expression levels. CpG sites in *AHRR*, *CYP1B1*, and *F2RL3* were located on the gene body, whereas those in *CYTL1* and *CYP1A1* were in the promoter. Our results are consistent with the expectation that promoter-based CpG sites negatively associate with gene expression [50–52]. Studies have reported both positive and negative correlations between methylation and expression for CpG sites in the gene body [53–56]. DNA methylation sites in the gene body that are negatively associated with expression levels may be located in alternative promoters that regulate the expression of particular isoforms.

*CYP1A1*, or cytochrome P4501A1, is a lung cancer susceptibility gene. Although in our data, *CYP1A1* smoking signals appear adipose-specific, independent studies have reported links to smoking in multiple tissues. *CYP1A1* smoking-associated methylation signals are present in the lung in the fetus [57] and in adults [30, 35]. In adults, effects are observed in normal lung tissue from lung cancer patients at both the *CYP1A1* promoter [35] and enhancer [30], which is also differentially methylated between normal tissue and lung tumor tissue [30]. A recent large-scale meta-analysis of smoking methylation signals in blood also reported a moderate effect at *CYP1A1* [20]. Maternal tobacco use was also associated with alterations in promoter methylation of placental *CYP1A1*, and these changes were correlated with *CYP1A1* gene expression and fetal growth restriction [58]. Furthermore, *CYP1A1* gene expression is downregulated by *AHRR*. *CYP1A1* is inducible by agonists of the aryl hydrocarbon receptor (AhR), which include environmental pollutants and components of cigarette smoke. Following activation of AhR by an agonist in the cytoplasm, the AhR-ligand complex translocates to the nucleus, where it dimerizes with the aryl hydrocarbon receptor nuclear translocator (ARNT) [59]. This heterodimer binds to the xenobiotic response element (XRE) site of *CYP1A1* in the upstream enhancer region, which activates transcription. *CYP1A1* metabolizes drug molecules and environmental pollutants, including polycyclic aromatic hydrocarbons, dioxin, and benzo(α)pyrene, into highly reactive intermediates. These derivatives can bind to DNA and form adducts, which may contribute to carcinogenesis [60]. AhR, in a complex with xenobiotic compounds and ARNT, induces *CYP1A1* expression, which subsequently detoxifies toxic components of cigarette smoke. *AHRR* suppresses the effects of *AhR* through binding to ARNT. Hypo-methylation of *AHRR* and increased *AHRR* expression may therefore reduce cellular responses to smoking, potentially through *CYP1A1* [61]. However, our findings of increased gene expression levels at both *AHRR* and *CYP1A1* in current smokers suggest that smoking-induced *AHRR* changes do not impact the *CYP1A1* response to smoking in adipose tissue. The smoking effects at *CYP1A1* in our study appear to be adipose-specific; therefore, these observations do not extend to blood, skin, or lung samples.

In addition to *CYP1A1*, other smoking signals that we identify in this study have also been previously linked to lung cancer. *CYP1B1* differentially methylated effects have been reported for smoking, for lung cancer, and for age at cancer diagnosis in non-small cell lung carcinoma (NSCLC) samples [62]. Several of our smoking signals were previously reported to be differentially methylated in lung adenocarcinoma tumor and matched non-tumor tissue [63]. These included two of our top smoking-DMS, *CYTL1* and *ACVRL1,* and seven of our top smoking-DES, *CYTL1*, *JAM2*, *CYGB*, *TAL1*, *GRIK3*, *SOX17*, and *TEK*.

In line with previous studies, we observe that genetic variation can impact the smoking-DMS, with potential implications for genotype influences on the rates of toxin elimination and nicotine metabolism in the human body. Importantly, we observe that all of the major smoking genetic variants detected in the largest smoking GWAS to date appear to influence DNA methylation levels in *cis*. These findings strongly suggest that DNA methylation may mediate some of the effects of genetic influences on smoking behavior, toxin elimination, or nicotine metabolism. We also replicate results from a genome-wide association study of nicotine metabolite ratio, identifying a 4.2-Mb region on chromosome 19q13 where GWAS SNPs were also associated with DNA methylation levels [43]. Taken together, these findings suggests some of the observed genetic impacts on smoking behavior and nicotine metabolism may be mediated by DNA methylation and that such effects are robust and shared across tissues.

Our analyses specifically in ex-smokers show variability in the extent of signal reversal over time, which is consistent with previous findings. We observe an overall trend towards at least partial reversal at most of the identified smoking-associated signals. Importantly, our study is the first to show that this trend is also observed in gene expression levels. Our findings suggest that smoking has a longer lasting influence on the methylome compared to the transcriptome, where the majority of reversal effects occur within the first year after smoking cessation.

The smoking-methylation signals were assessed for association with adiposity phenotypes that constitute major metabolic disease risk. Significant associations were observed between visceral fat mass and android-to-gynoid fat ratio with methylation levels at smoking markers with functional impacts on gene expression, such as *CYP1A1* with replication, and in signals that were shared across tissues, such as *NOTCH1*. Associations were also detected with smoking-DES. These results may help improve our understanding of how smoking impacts metabolic health, and to explore this further, we considered smoking effects on future changes in metabolic phenotypes on smoking cessation. Visceral fat has a strong association with obesity-related metabolic diseases, such as type 2 diabetes and cardiovascular disease [64, 65] and is a major metabolic disease risk factor. At smoking markers *BHLHE40* and *AHRR*, DNA methylation and gene expression levels in current smokers were predictive of future gain in visceral fat observed after smoking cessation. Although the sample size of current smokers who go on to quit smoking in our data is modest, these findings provide an interesting insight into potential molecular mechanisms mediating environmental effects on metabolic disease risk and require replication in larger samples.

A limitation to our study is partial correction for the influence of expected covariates. These include, first, alcohol consumption, which co-occurs with smoking. In our co-occurrence analyses, none of the alcohol-associated CpG sites reached genome-wide significance after adjusting for smoking. In a previous alcohol EWAS in blood, Liu et al. [66] also found that the effect size of the majority alcohol-DMS was not affected by smoking status suggesting that despite their co-occurrence, smoking and alcohol impact DNA methylation in different aspects. A related question is optimal correction for cell composition in adipose tissue. Since we only had access to subcutaneous adipose tissue biopsies, rather than isolated cell subtypes, we corrected for cell composition by using the analytical approach within the reference-free EWAS [32] framework and found that the majority of results remained largely unchanged. However, it is possible that this does not fully capture the effect of a heterogeneous population of cells as a confounder. Some of the smoking-DMS such as *BHLHE40*, which was also found to be predictive of future gain in visceral fat, may reflect cell-specific methylation profiles. *BHLHE40* was previously reported to be hypo-methylated in activated NK cells (but not in naive NKs, T, and B cells) [67] and a similar trend was observed for *AHRR* [67]*.* One interpretation of these findings is that some smoking signals are cell subtype specific [68, 69], potentially reflecting a selective enhancement of activated cells, because smoking can also induce changes in blood count [70]. In adipose tissue, this particular effect may be represented as an infiltration of activated NK cells, and this infiltration may increase with obesity, diabetes, and smoking. On the other hand, the relative abundance of NK DNA compared with adipose DNA in adipose tissue is minimal; therefore, these effects should be minimal. Future studies are needed to assess the impact of these potential confounding effects using for example histological and immunological staining of adipose tissue.

## Conclusion

Our results show that smoking can impact DNA methylation and gene expression levels in adipose tissue. To our knowledge, this is the first study that performed genome-wide analyses of smoking in adipose tissue DNA methylation and gene expression profiles. The key results are that, first, smoking leaves a signature on both the methylome and transcriptome with overlapping signals; second, smoking methylation signals tend to be tissue-shared effects; third, smoking has a longer lasting influence on DNA methylation levels than on gene expression after smoking cessation; and forth, specific smoking methylation and expression signals are associated with metabolic disease risk phenotypes, as well as future weight gain after smoking cessation.

## Methods

### Study population and sample collection

The primary sample of subjects is twins from the TwinsUK cohort who were recruited as part of the MuTHER (Multiple Tissue Human Expression Resource) study [71]. All subjects are Caucasian females and ascertained to be free from severe disease when the samples were collected. The sample consisted of 542 female twins, comprising 54 current smokers, 197 ex-smokers, and 291 non-smokers. The 542 twins included 84 MZ twin pairs, 112 DZ twin pairs, and 150 unrelated individuals (Additional file 2: Table S6). Adipose tissue biopsies were obtained from all subjects between August 2007 and May 2009. Details of biopsy procedures and sample descriptions are described previously [72]. Briefly, subcutaneous adipose tissue biopsies were dissected from near the umbilicus of the abdominal region; the fat layer was separated from skin layers and stored immediately in liquid nitrogen. Both DNA and RNA were extracted from the same adipose tissue biopsy, as previously described [72, 73]. Ethical approval was granted by the National Research Ethics Service London-Westminster, the St Thomas’ Hospital Research Ethics Committee (EC04/015 and 07/H0802/84). All research participants have signed an informed consent prior to taking part in any research activities.

To explore tissue-shared effects, peripheral blood samples from 789 and 362 subjects from TwinsUK were also explored for genome-wide methylation and expression profiling, respectively. The blood samples for methylation measurement were taken from 1992 to 2012, and the samples for gene expression measurement were taken from 2007 to 2009. From the 542 subjects with available adipose tissue samples, 200 and 222 subjects donated blood samples for methylation and expression profiling, respectively. Blood samples and adipose tissues were collected during the subject’s visit to the clinic.

The majority of data analysis focused on methylation and expression level differences between current smokers and non-smokers. The sample subsets of current smokers and non-smokers comprised 345 subjects in adipose methylation and expression samples, 567 subjects in blood methylation samples, and 237 in blood expression samples.

### Replication and validation analyses

The 42 smoking-DMS that we identified in the TwinsUK cohort were further explored in five independent datasets: (1) 104 subjects from the LEAP cohort were used for adipose smoking-DMS replication (dataset 1); (2) 69 subjects from the Finnish Twins were used for replication of methylation associations with metabolic phenotypes (dataset 2); and (3) 195 subjects (skin, dataset 3), (4) 168 subjects (lung, dataset 4), and (5) 567 subjects (blood, dataset 5) were used to explore tissue-shared effects at the 42 smoking-DMS. Details of dataset 5 were described in the previous section.

#### Dataset 1: LEAP cohort adipose tissue (USA)

To replicate the 42 adipose tissue smoking-DMS, we studied 104 participants from the New England Family Study, the LEAP cohort (mean BMI 30.9 ± 7.03, mean age 47 ± 1.7, 48% male; see Additional file 2: Table S6), described in detail elsewhere [39]. The individuals are of mixed ancestry (63.5% white) and were not affected with disease. There were 46 current smokers and 58 non-smokers. Subcutaneous adipose tissue samples in these participants were collected from the upper outer quadrant of the buttock, followed by DNA extraction, and Infinium HumanMethylation450 BeadChip array profiling as previously described [37]. Replication analyses were performed using a linear regression model adjusting for age, gender, BMI, and batch effect.

#### Dataset 2: Finnish twin adipose tissue

To replicate the methylation associations with metabolic health traits, we studied 69 Finnish twins (mean age 31.1 ± 4.43 years, mean BMI 27.5 ± 4.72, 44.9% male; see Additional file 2: Table S6), who were recruited as a part of the Finnish twin cohort. Adipose tissue sample collection and DNA extraction in this sample have been previously described in detail [74, 75]. The sample included 34 full MZ twin pairs and 21 current smokers. DNA methylation profiling was measured by Infinium HumanMethylation450 BeadChip array and TFM and AGR were determined by dual energy X-ray absorptiometry (DEXA). Replication analyses were performed using a linear mixed effect regression model adjusting for age, gender, BMI, family, batch effect, and alcohol intake. Sample characteristics of the replication cohorts are shown in Additional file 2: Table S6.

To examine the tissue specificity of the 42 smoking-DMS, we included two additional datasets (dataset 3 and dataset 4) for validation of tissue-shared smoking effects.

#### Dataset 3: TwinsUK skin tissue

The first validation dataset for identifying tissue-shared effects included 195 skin tissue samples from twins (mean age 59.1 ± 9.71 years, mean BMI 26.7 ± 4.71; see Additional file 2: Table S6) from the TwinsUK cohort. This analysis included 37 current smokers and 158 non-smokers cancer-free female subjects only, and some subjects also provided adipose samples in the current study. The TwinsUK skin samples and the evaluation of DNA methylation in the samples are described elsewhere [34]. We performed the analysis using a linear mixed effects model adjusting for age, BMI, alcohol consumption, batch effect, family structure, and zygosity. Sample characteristics are shown in Additional file 2: Table S6.

#### Dataset 4: Lung cancer tissue

The second validation dataset for identifying tissue-shared smoking effects included 168 lung cancer female subjects (mean age 65.1 ± 10.66 years; see Additional file 2: Table S6) from a multicenter cohort of 450 subjects with non-small cell lung cancer (GEO dataset: GSE39279) [76]. In the validation analysis, we selected only female subjects who had smoking records (129 current smokers and 39 non-smokers) and used a linear regression model to test for the effect of smoking on methylation, adjusting for age, cancer stage [1 to 4], and cancer type (adenocarcinoma or squamous). DNA methylation levels were obtained using the Infinium HumanMethylation450 BeadChip, and BMIQ normalization was performed prior to analysis.

### Phenotype collection

During a subject’s clinical visit, basic demographic information was collected, with on-site measurements such as height and weight, DEXA measurements, and biopsy collection. Self-reported smoking status is obtained through longitudinal questionnaires. Data included answers to the following questions: “Do you currently smoke (more than 3 days per week)?” (yes/no), “How long has it been since you gave up smoking (in years/months)?”, “How long have you smoked for in total (in years/months)?”, “On average how many cigarettes do you smoke a day (cigarette numbers)?”, “How many cigarette you smoke in the past 100 days (cigarette numbers)?”. Longitudinal data were available for each subject, and we excluded subjects who did not have consistent longitudinal smoking records. Smoking status was defined in three categories: current smokers, ex-smokers, and non-smokers. Current smokers were defined as subjects who consistently smoked cigarettes (and have not stopped at any point) according to their longitudinal records up to the clinical visit when the adipose tissue biopsy was obtained. Ex-smokers were individuals who have successfully (and consistently) reported to have quit smoking cigarettes for at least 3 months prior to the adipose tissue biopsy. Non-smokers were individuals who never smoked according to the longitudinal questionnaire records. Other phenotypes such as age, body mass index (BMI), and alcohol consumption were also collected during the clinical visit. The alcohol consumption data were obtained by questionnaires, and subjects were asked about the quantity (mL) and beverage types (beer, cider, lager, wine, spirits) drank per week. We then summarized the total alcohol intake as units per week and then converted to grams/day (one unit of alcohol in the UK is defined as 7.9 g [77]). Adiposity phenotypes, such as total fat mass (TFM), visceral fat mass (VFM), and android-to-gynoid ratio (AGR) were measured by DEXA scan.

Some participants have regular clinical research visits, approximately every 2 years. To study the weight gain after smoking cessation, we used phenotype information for VFM collected at two time points: the first time point is the date nearest to the adipose tissue collection date, and the second time point is the most recent clinical research visit of the subject where VFM data were collected. The time between longitudinal clinical research visits used in this study ranged between 3 and 7 years with a mean of 5.1 ± 0.70.

### Genome-wide DNA methylation profiles

The Infinium HumanMethylation450 BeadChip (Illumina Inc., San Diego, CA) was used to measure DNA methylation in both adipose and blood samples. Details of experimental approaches have been previously described [72, 78]. At each probe, the methylation levels are characterized as a finite bounded quantitative trait ranging between 0 and 1, and represented as beta values. To overcome biases caused by the two Illumina probe types and two-color channels [79], we performed the beta mixture quantile dilation (BMIQ) method [80] and background correction for each sample. DNA methylation probes that mapped incorrectly or to multiple locations in the reference sequence were removed. Probes with more than 1% of subjects with detection *P* value > 0.05 were also removed. All the probes have non-missing values in blood samples and less than 1% missing subjects in adipose samples. Probes located on chromosomes X and Y were removed from the analysis. To check for sample swaps, we compared 65 single nucleotide polymorphism (SNP) markers that featured as control probes on the array to genotypes for each subject and removed subjects with incomparable genotypes. Because methylation levels on the majority of probes do not follow the normal distribution, which might violate the regression assumption for downstream analysis, we normalized the methylation levels to *N*(0,1) prior to analysis. For all the other methylation datasets (USA adipose, Finnish adipose, lung cancer tissues, TwinsUK skin samples, and TwinsUK blood samples), we performed exactly the same quality control steps for data cleaning and normalization prior to the analysis.

### RNA-sequencing data

The twin adipose RNA-seq data and quality control have been previously described [81, 82]. Briefly, sequenced paired-end reads (49 bp) were mapped to the human genome (GRCh37) by Burrows-Wheeler aligner (BWA) software v0.5.9 [83], then genes were annotated as defined by protein coding in GENCODE v10 [84]. Samples were excluded if they failed during library preparation or sequencing. Samples were only considered to have good quality if more than 10 million reads were sequenced and mapped to exons. Gene expression levels were quantified per gene, estimated as RPKM values (reads per kilobase of transcript per million mapped reads) and rank normal transformed prior to analysis. The genotype of each subject was used for identity checks in case of sample swaps. After removing genes located on chromosomes X and Y, and non-coding transcripts, 17,399 genes were included in the gene expression analysis for adipose tissues and blood samples.

### Genotype data

Genotypes were available for all subjects in study. Genotyping of the larger TwinsUK dataset was performed using HumanHap300, HumanHap610Q, HumanHap1M Duo, and HumanHap1.2M Duo 1M arrays. Imputation was done in two datasets separately and subsequently merged with GTOOL. Genotype data were pre-phased using IMPUTE2 without a reference panel, then using the resulting haplotypes to perform fast imputation from 1000 Genome phase1 dataset [85, 86]. We used 1000 Genomes Phase I (interim) as reference set, based on a sequence data freeze from 23 Nov 2010; the phased haplotypes were released Jun 2011. After imputation, SNPs were filtered at a MAF > 5%. Genotypes were used for identification of meQTLs and eQTLs in the 542 adipose samples.

### Statistical analysis

#### Differential methylation and expression analyses

Principal component analysis (PCA) was used to identify potential batch effects and covariates to be included in the statistical model for both methylation and gene expression adipose data. To identify the adipose methylation differences between current smokers and non-smokers, a linear mixed effect regression model (LMER) was applied adjusting for batch effects (plate, position on the plate, bisulfite conversion levels, and bisulfite conversion efficiency), age, BMI, alcohol consumption, family and zygosity structure. In the blood, the methylation differences between current smokers and non-smokers were tested adjusting for batch effects (plate and position on the plate), age, BMI, alcohol consumption, and seven predicted cell count estimates (plasma blast, CD8pCd28nCD45Ran, CD8 naïve, CD4T, NK, monocytes, and granulocytes), family and zygosity structure. Blood cell counts were calculated using the Horvath online calculator [87]. A linear mixed effect regression model was applied as the data contained MZ and DZ twins. Family structure and zygosity were included as random effect terms, while all the other covariates were included as fixed effect terms. Similarly, in the RNA-seq data analysis, the adipose and blood expression differences between current smokers and non-smokers were examined using LMER adjusting for age, BMI, alcohol consumption (grams/day), GC mean, primer index, clinic visit date, family structure, and zygosity. Family structure, zygosity, primer index, and clinic visit date were taken as random effect, and all the other covariates were included as fixed terms. For each CpG site or gene, a full model that regressed all of the covariates was compared to a null model that excluded smoking status. The models were compared using the ANOVA F statistic. A genome-wide significance level was set at 1% false discovery rate for all analyses.

In order to account for mixtures of cell types in adipose tissue, we performed a EWAS using the reference-free approach proposed by Houseman et al. [32]. The method is similar to surrogate variable analysis (SVA) and independent surrogate variable analysis (ISVA), which is used to adjust for technical errors (e.g., batch effect) and confounders. In addition, the reference-free approach also includes a bootstrap step to account for the correlation in the structure of standard errors. Using this approach, we can estimate direct epigenetic effects that account for cell compositions and use bootstrap-based *P* values to assess their significance. Due to the limitation that the reference-free approach can currently only be applied to datasets of unrelated individuals, we used 251 unrelated individuals from the original 542 twins and compared the top results between two EWASs.

To identify tissue-shared smoking differentially methylated signals across adipose and whole blood datasets, we compared the genome-wide FDR 1% signals across adipose and whole blood DNA methylation analyses. In whole blood samples, we tested for association between smoking status and DNA methylation levels at 452,874 CpG sites in 86 current and 481 non-smokers in blood. We compared the FDR 1% adipose DMS to 2782 CpG sites that were associated with smoking in blood at FDR 1% (*P* = 1.14 × 10^−5^). To further explore tissue specificity in other tissues, we explored the 14 tissue-shared smoking-DMS identified in both adipose and blood samples. We used previously published datasets of 196 cancer-free female subjects with skin tissue biopsies [34] and a lung cancer DNA methylation dataset [76], applying a Bonferroni-adjusted *P* value of 3.6 × 10^−4^ as the significance threshold.

#### Receiver operative curve (ROC) analysis

We tested several models for predicting smoking status based on the different combinations of the adipose smoking-DMS and smoking-DES. The sensitivity and specificity of these prediction models were calculated using receiver operative curve (ROC). The ROC analysis was performed in R using the “pROC” package [88] with the “lme” function for logistic regression, where outcomes are categorized as current smokers and non-smokers. We then used the “predict” function to predict the expected probabilities under different combinations of predicting factors (methylation levels of 14 CpG sites and expression levels at five genes), and the “roc” function to predict the sensitivity and specificity and draw the area under the curve. We selected 27 current smokers and 145 non-smokers as a training set to construct a logistic model for smoking status classification, and then used the remaining set of 173 subjects (27 current smokers) as a validation set, in which we obtained the AUC values. We repeated this procedure 1000 times and report the average AUC values across 1000 validation sets.

#### Smoking cessation analyses

We quantified “reversal” time by estimating the time (in smoking-quit years) required for ex-smokers to revert to 25% of the change in methylation towards non-smokers. We first calculate the difference between methylation levels in current smokers and those in non-smokers and use 25% change of that difference as a “reversal” threshold. For example, at cg05575921 in *AHRR*, the median level of methylation residual is − 0.234 in current smokers and 0.037 in non-smokers, resulting in a 0.271 methylation change. Therefore, ex-smokers who reached methylation levels of − 0.031 were classified as subjects who “reversed”. We quantified the proportion of subjects who reversed within different quit years. For example, at cg05575921, 6 ex-smokers quit in less than 1 year, but only one had methylation reverting to 25% of the methylation change towards non-smokers; therefore, the reversal rate was 16.7%. We quantified reversal at the gene expression level using the same approach.

#### Methylation QTL (meQTL) analyses

Genome-wide meQTL analyses were performed testing for the association between common genetic variants and DNA methylation at CpG sites in the two adipose tissue samples. We only considered SNPs that were significantly associated with DNA methylation in *cis* to be meQTLs. If multiple SNPs were identified for a single CpG site, we reported only the most significant SNP per CpG site (*P* = 5 × 10^−5^, as described in Grundberg et al. [72]). In total, methylation levels of 102,461 CpG sites were associated with genetic factors in *cis*, and 25,531 sites in *trans*.

We tested for adipose tissue meQTLs first by fitting a LME model regressed all the identified covariates, then performed a linear regression of the residuals on the SNPs using the MatrixeQTL R package [89]. Results from meQTL analyses are presented at a *P* value of 10^−5^ for the smoking-DMS, the smoking-DES, and at the smoking GWAS genetic variants. For meQTL analyses replicating the results from Loukola et al. [43], we applied a different threshold. Loukola et al. [43] conducted a genome-wide association study of nicotine metabolite ratio, identifying many strongly associated SNPs in a 4.2-Mb region on chromosome 19q13. Among the 158 CpG sites within that region, 16 CpG sites showed statistically significant association with 173 SNPs. We compared our meQTL findings to those from Loukola et al. [43] at a modified Bonferroni significance threshold of 1.81 × 10^−5^ (= 0.05/(16 × 173)) and identified SNPs that influence methylation levels at 5 CpG sites (in *CYP2A7*, *ENGL2*, and *LTBP4* genes) (Additional file 2: Table S5).

### Correlations between methylation and gene expression levels

We compared the 542 subjects’ adipose methylation and gene expression levels at the five overlapping genes identified in the two genome-wide association analyses. Both the methylation and expression data were first adjusted for covariates, and Spearman’s correlation test was then performed on the residuals.

### Metabolic disease risk phenotype analyses

We studied the impacts of smoking methylation signals on obesity and metabolic phenotypes. We explored 288 adipose subjects (42 current smokers and 246 non-smokers, mean BMI = 26.70 ± 4.62) who had available DEXA profiles at or within up to 1 year of the adipose tissue biopsy. We compared the association between DNA methylation and the adiposity phenotypes, such as visceral fat mass (VFM), total fat mass (TFM), and android-to-gynoid fat ratio (AGR). Analyses were carried out at the 42 smoking-DMS using a linear regression model adjusting for BMI, age, and smoking status. A significance level was set at a Bonferroni-adjusted threshold of *P* = 5.7 × 10^−4^. We used a similar approach to test for phenotype associations with the 42 smoking-DES.

To further investigate the effect of 42 smoking-DMS and 42 smoking-DES on weight gain after smoking cessation, the adiposity phenotype differences were obtained at two time points in a reduced sample size of 248 subjects. Depending on a subjects’ smoking behavior at the two time points, we categorized subjects into five categories: current smokers at the two time points (constant smokers, S-S, *n* = 12), current smokers at time point 1 who quit smoking by time point 2 (S-E, *n* = 5), ex-smokers (who quit smoking within 1–5 years) at time point 1 who remain ex-smokers at time point 2 (E1-E5, *n* = 13), ex-smokers who quit > 5 years at time point 1 who remain ex-smokers at time point 2 (E5+, *n* = 92), and non-smokers at both time points (never smokers, N-N, *n* = 124). We then calculated the phenotype differences (phenotype at time point 2 minus phenotypes at time point 1) for each subject and correlated this differences to their adipose methylation (42 smoking-DMS) and gene expression levels (42 smoking-DES).

We used the R statistical software (https://www.r-project.org/) for all analyses and figures, and the regional plots were generated using the coMET package [90].

## Additional files

Additional file 1: **Figure S1.** Scatterplot of correlations between EWAS -log_10_P-values from the linear mixed effect model used in the current study adipose discovery sample (y-axis) and results from Reference-free EWAS approach proposed by Houseman et al. (x-axis) [32]. **Figure S2.** Smoking cessation and adipose DNA methylation profiles. DNA methylation levels at the 42 smoking-DMS and smoking status in 542 adipose samples. Subject groups include current smoker, subjects who quit smoking within one year, subjects who quit between 1 to 5 years, subjects who quit smoking more than 5 years, and subjects who never smoked. Fourteen CpG sites located in genes with both smoking-DMS and smoking-DES are denoted with asterisks. **Figure S3.** Smoking cessation and adipose gene expression profiles. Gene expression levels at the 42 smoking-DES and smoking status in 542 adipose samples. Subject groups include current smoker, subjects who quit smoking within one year, subjects who quit between 1 to 5 years, subjects who quit smoking more than 5 years, and subjects who never smoked. Five genes with both smoking-DMS and smoking-DES are denoted with asterisks. **Figure S4.** Association between DNA methylation levels at the 42 smoking-DMS and future change in visceral fat mass (VFM) in 18 (red solid dots) and 228 subjects (gray hollow dots). **Figure S5.** Association between gene expression levels at the 42 smoking-DES and future change in visceral fat mass (VFM) in 18 (blue solid dots) and 228 subjects (gray hollow dots). (PDF 1411 kb)

Additional file 2: **Table S1.** Four smoking-induced differentially methylated and expressed genes in blood samples. **Table S2.** Validation of 14 tissue-shared smoking-DMS across four sample types. **Table S3.** Replication of the 42 smoking-DMS in the LEAP cohort [39] with 104 current smokers and non-smokers. **Table S4.** Previously-identified smoking genetic variants and their impacts on DNA methylation and gene expression in adipose tissue. **Table S5.** DNA methylation QTL (meQTLs) analyses at the chromosome 19 region from Loukola et al. [43], showing replication in TwinsUK adipose tissue samples. **Table S6.** Characteristics of TwinsUK (adipose tissue, blood samples, and skin tissue [34]), Finnish cohort [74, 75], LEAP cohort [39], and lung cancer [76] samples. (XLSX 43 kb)

## Abbreviations

AGR: Android-to-gynoid fat ratio
AhR: Aryl hydrocarbon receptor
AUC: Area under curve
BMI: Body mass index
BWA: Burrows-Wheeler aligner
DES: Differentially expressed signals
DEXA: Dual energy X-ray absorptiometry
DMS: Differentially methylated signals
EWAS: Epigenome-wide association study
FDR: False discovery rate
LME model: Linear mixed effect model
MAF: Minor allele frequency
meQTL: Methylation quantitative trait locus
NSCLC: Non-small cell lung carcinoma
PCA: Principal component analysis
RNA-seq: RNA-sequencing
ROC: Receiver operative curve
SE: Standard error
SNP: Single nucleotide polymorphism
SVA: Surrogate variable analysis
TFM: Total fat mass
TSS: Transcription start site
TWAS: Transcriptome-wide association study
VFM: Visceral fat mass
