# Supplementary material for: Smoking induces coordinated DNA methylation and gene expression changes in adipose tissue with consequences for metabolic health
Source: Clin Epigenetics. 2018 Oct 20;10:126. doi: 10.1186/s13148-018-0558-0 (PMC6196025; doi:10.1186/s13148-018-0558-0)
Supplement: Supplementary file 1 — Figure S1. Scatterplot of correlations between EWAS -log10P-values from the linear mixed effect model used in the current study adipose discovery sample (y-axis) and results from Reference-free EWAS approach proposed by Houseman et al. (x-axis) [32]. Figure S2. Smoking cessation and adipose DNA methylation profiles. DNA methylation levels at the 42 smoking-DMS and smoking status in 542 adipose samples. Subject groups include current smoker, subjects who quit smoking within one year, subjects who quit between 1 to 5 years, subjects who quit smoking more than 5 years, and subjects who never smoked. Fourteen CpG sites located in genes with both smoking-DMS and smoking-DES are denoted with asterisks. Figure S3. Smoking cessation and adipose gene expression profiles. Gene expression levels at the 42 smoking-DES and smoking status in 542 adipose samples. Subject groups include current smoker, subjects who quit smoking within one year, subjects who quit between 1 to 5 years, subjects who quit smoking more than 5 years, and subjects who never smoked. Five genes with both smoking-DMS and smoking-DES are denoted with asterisks. Figure S4. Association between DNA methylation levels at the 42 smoking-DMS and future change in visceral fat mass (VFM) in 18 (red solid dots) and 228 subjects (gray hollow dots). Figure S5. Association between gene expression levels at the 42 smoking-DES and future change in visceral fat mass (VFM) in 18 (blue solid dots) and 228 subjects (gray hollow dots). (PDF 1411 kb) [file 13148_2018_558_MOESM1_ESM.pdf]

Figure S1

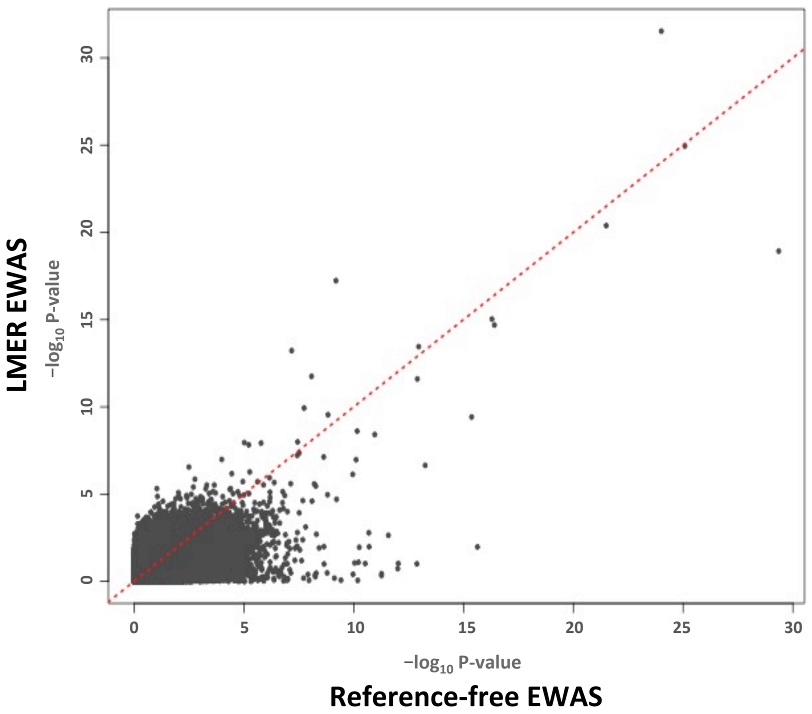

LMER EWAS
−log10 P-value
0
5
10
15
20
25
30
−log10 P-value
Reference-free EWAS

Figure S2

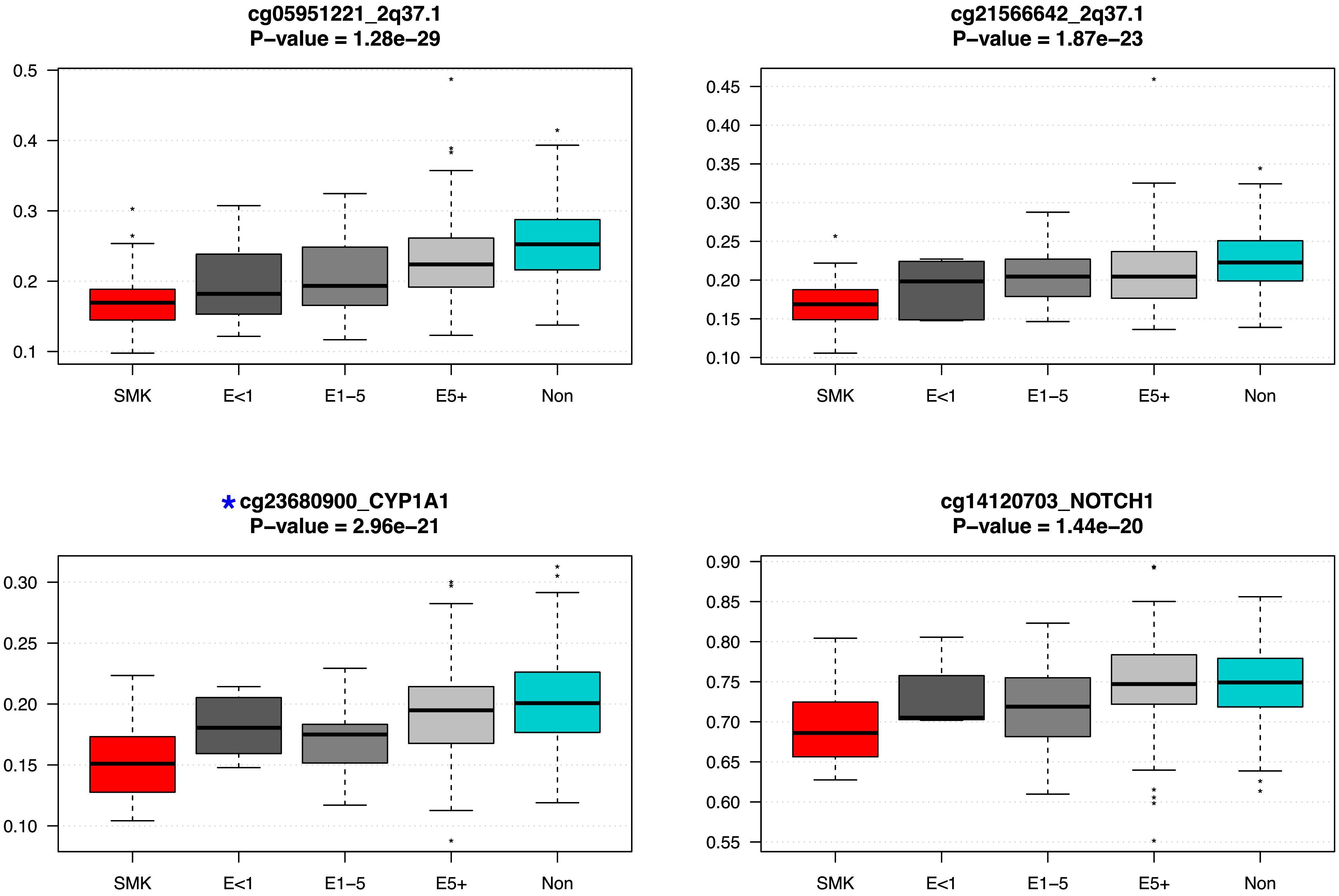
cg05951221_2q37.1
P–value = 1.28e–29
0.1
0.2
0.3
0.4
0.5
SMK
E<1
E1–5
E5+
Non
cg21566642_2q37.1
P–value = 1.87e–23
0.10
0.15
0.20
0.25
0.30
0.35
0.40
0.45
SMK
E<1
E1–5
E5+
Non
* cg23680900_CYP1A1
P–value = 2.96e–21
0.10
0.15
0.20
0.25
0.30
SMK
E<1
E1–5
E5+
Non
cg14120703_NOTCH1
P–value = 1.44e–20
0.55
0.60
0.65
0.70
0.75
0.80
0.85
0.90
SMK
E<1
E1–5
E5+
Non

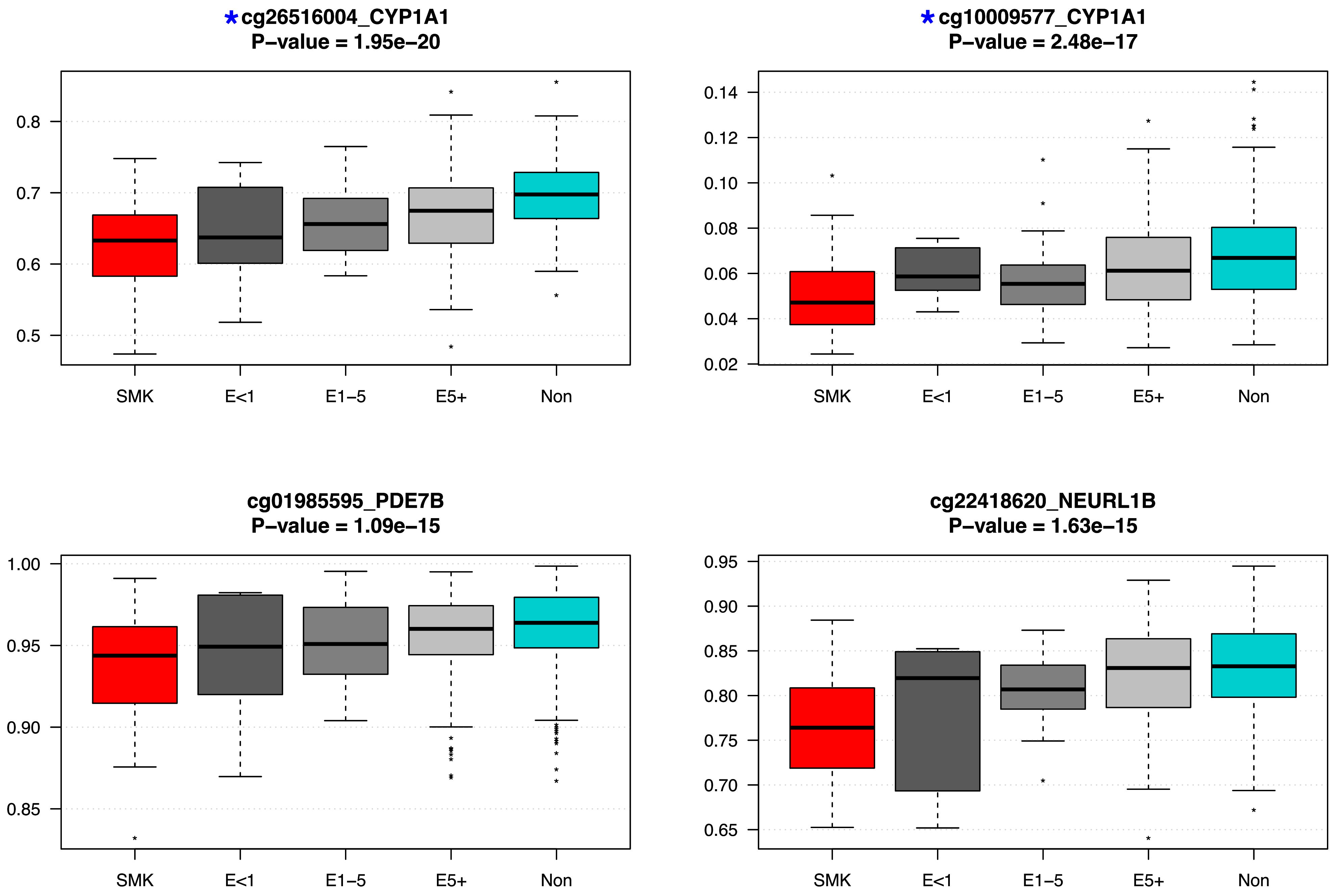
*cg26516004_CYP1A1
P–value = 1.95e–20
0.8
0.7
0.6
0.5
SMK
E<1
E1–5
E5+
Non
*cg10009577_CYP1A1
P–value = 2.48e–17
0.14
0.12
0.10
0.08
0.06
0.04
0.02
SMK
E<1
E1–5
E5+
Non
cg01985595_PDE7B
P–value = 1.09e–15
1.00
0.95
0.90
0.85
SMK
E<1
E1–5
E5+
Non
cg22418620_NEURL1B
P–value = 1.63e–15
0.95
0.90
0.85
0.80
0.75
0.70
0.65
SMK
E<1
E1–5
E5+
Non

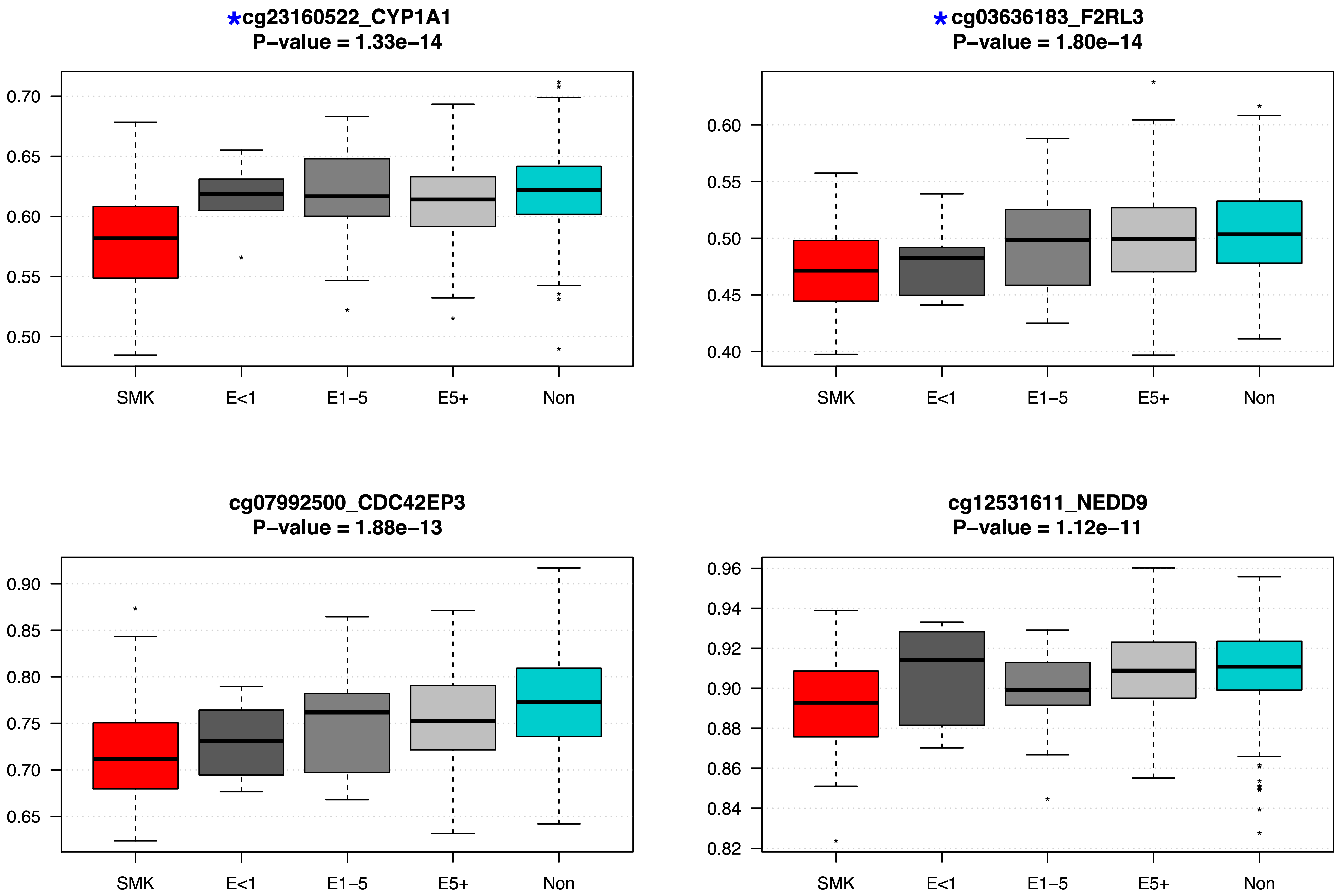
*cg23160522_CYP1A1
P–value = 1.33e–14
0.70
0.65
0.60
0.55
0.50
SMK
E<1
E1–5
E5+
Non
*cg03636183_F2RL3
P–value = 1.80e–14
0.60
0.55
0.50
0.45
0.40
SMK
E<1
E1–5
E5+
Non
cg07992500_CDC42EP3
P–value = 1.88e–13
0.90
0.85
0.80
0.75
0.70
0.65
SMK
E<1
E1–5
E5+
Non
cg12531611_NEDD9
P–value = 1.12e–11
0.96
0.94
0.92
0.90
0.88
0.86
0.84
0.82
SMK
E<1
E1–5
E5+
Non

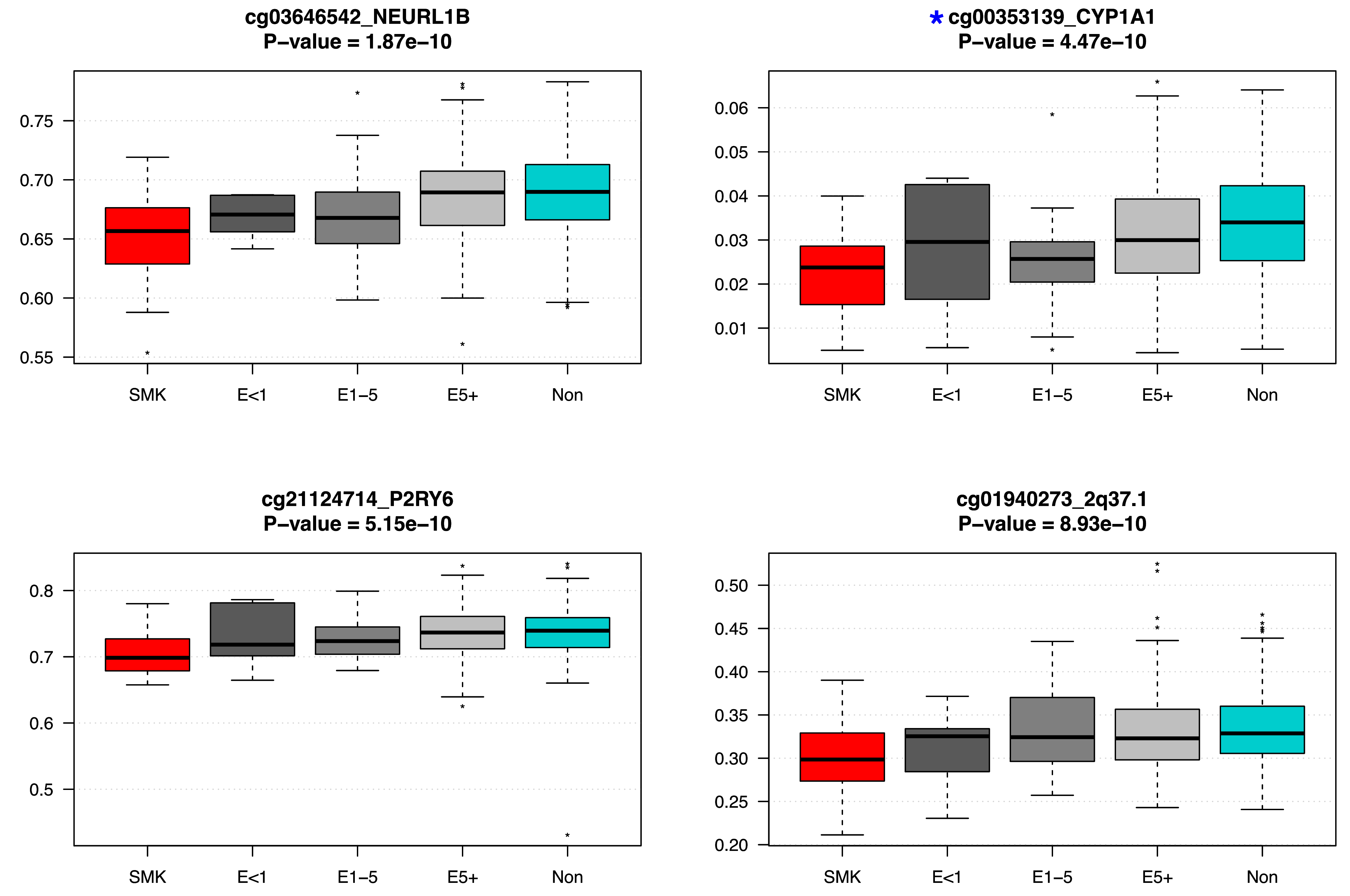
cg03646542_NEURL1B
P–value = 1.87e–10
0.55
0.60
0.65
0.70
0.75
SMK
E<1
E1–5
E5+
Non
* cg00353139_CYP1A1
P–value = 4.47e–10
0.01
0.02
0.03
0.04
0.05
0.06
SMK
E<1
E1–5
E5+
Non
cg21124714_P2RY6
P–value = 5.15e–10
0.5
0.6
0.7
0.8
SMK
E<1
E1–5
E5+
Non
cg01940273_2q37.1
P–value = 8.93e–10
0.20
0.25
0.30
0.35
0.40
0.45
0.50
SMK
E<1
E1–5
E5+
Non

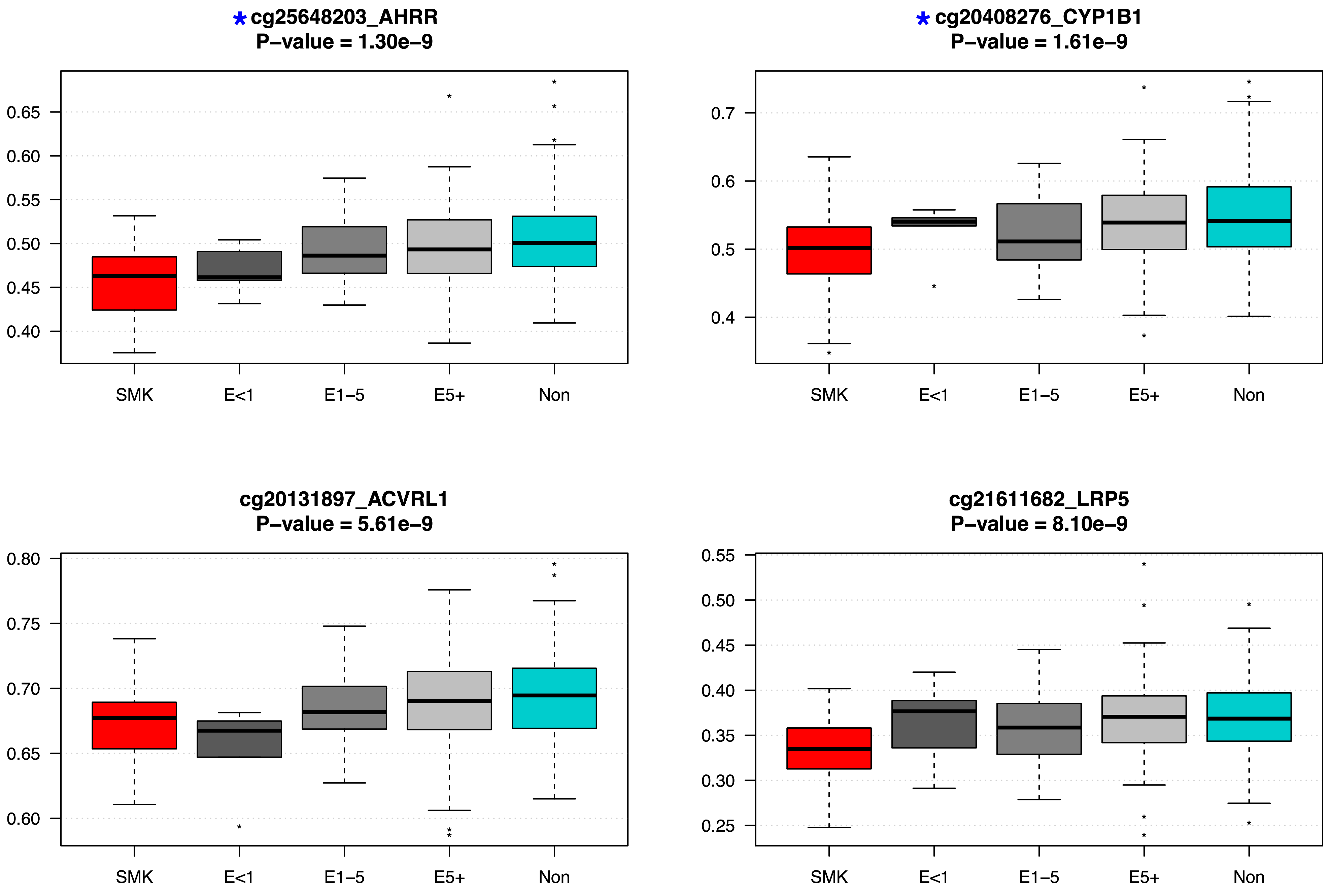

*cg25648203_AHRR
P−value = 1.30e−9
0.65
0.60
0.55
0.50
0.45
0.40
SMK
E<1
E1−5
E5+
Non
*cg20408276_CYP1B1
P−value = 1.61e−9
0.7
0.6
0.5
0.4
SMK
E<1
E1−5
E5+
Non
cg20131897_ACVRL1
P−value = 5.61e−9
0.80
0.75
0.70
0.65
0.60
SMK
E<1
E1−5
E5+
Non
cg21611682_LRP5
P−value = 8.10e−9
0.55
0.50
0.45
0.40
0.35
0.30
0.25
SMK
E<1
E1−5
E5+
Non

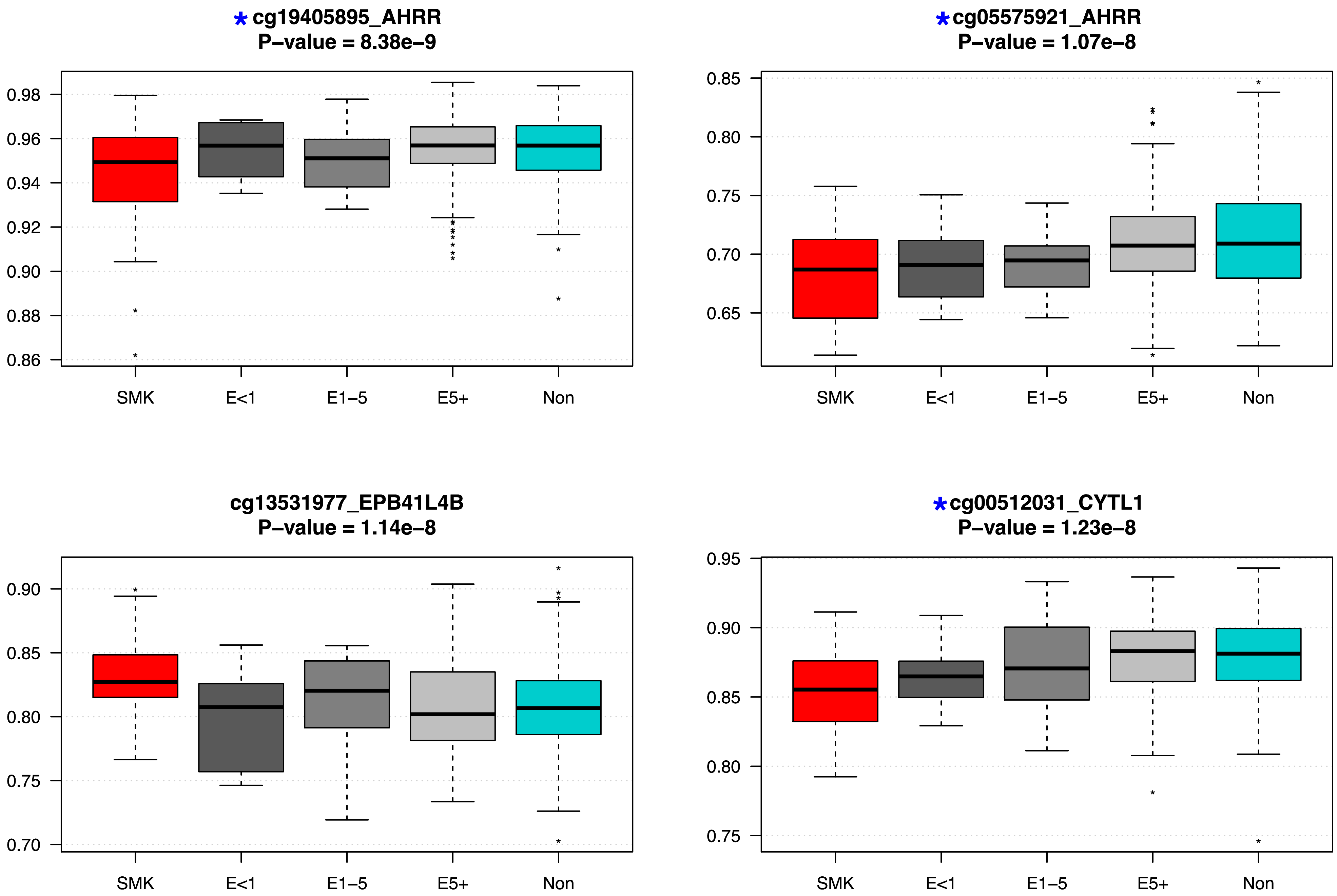
*cg19405895_AHRR
P−value = 8.38e−9
0.98
0.96
0.94
0.92
0.90
0.88
0.86
SMK
E<1
E1−5
E5+
Non
*cg05575921_AHRR
P−value = 1.07e−8
0.85
0.80
0.75
0.70
0.65
SMK
E<1
E1−5
E5+
Non
cg13531977_EPB41L4B
P−value = 1.14e−8
0.90
0.85
0.80
0.75
0.70
SMK
E<1
E1−5
E5+
Non
*cg00512031_CYTL1
P−value = 1.23e−8
0.95
0.90
0.85
0.80
0.75
SMK
E<1
E1−5
E5+
Non

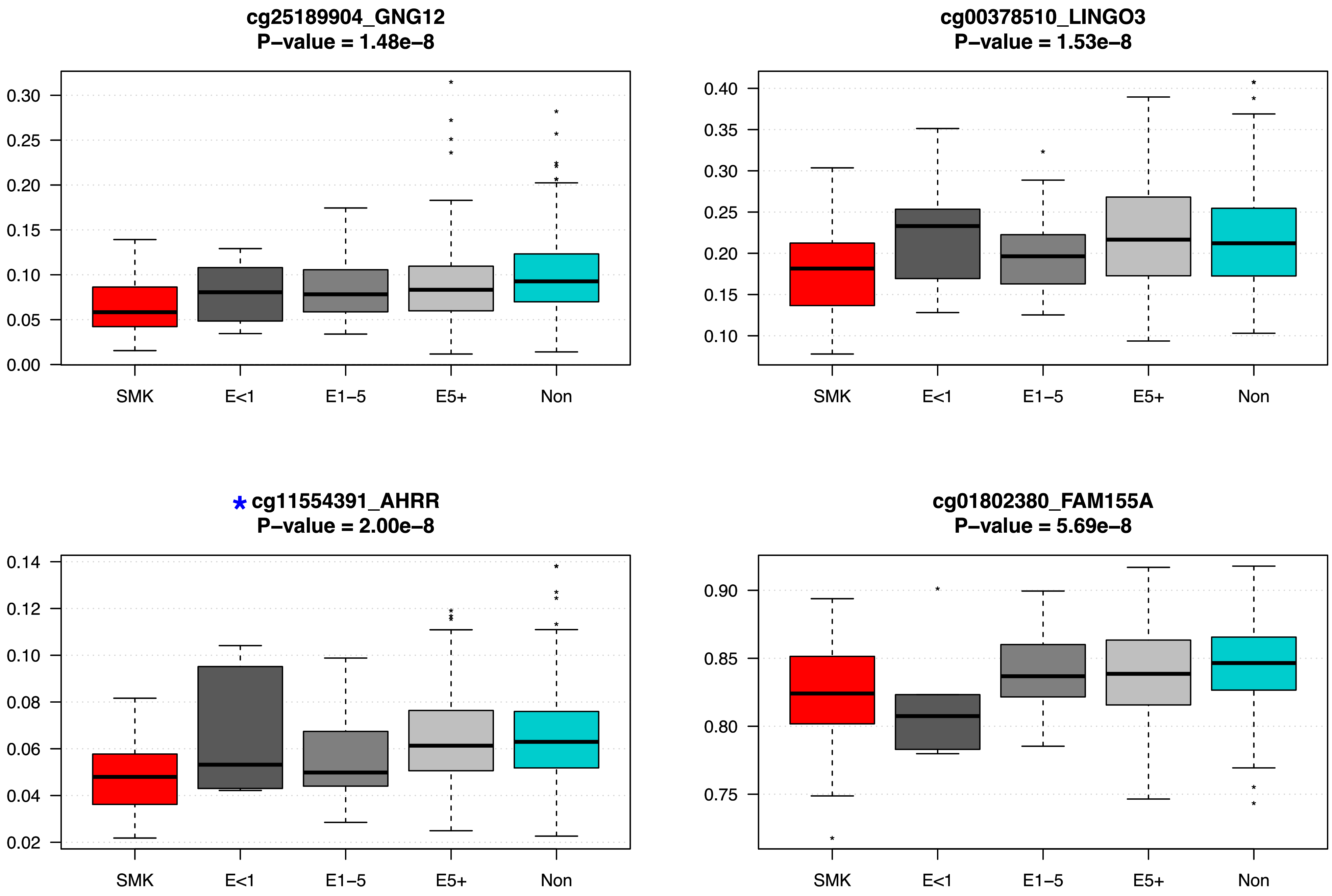

cg25189904_GNG12
P−value = 1.48e−8
0.30
0.25
0.20
0.15
0.10
0.05
0.00
SMK
E<1
E1−5
E5+
Non
cg00378510_LINGO3
P−value = 1.53e−8
0.40
0.35
0.30
0.25
0.20
0.15
0.10
SMK
E<1
E1−5
E5+
Non
* cg11554391_AHRR
P−value = 2.00e−8
0.14
0.12
0.10
0.08
0.06
0.04
0.02
SMK
E<1
E1−5
E5+
Non
cg01802380_FAM155A
P−value = 5.69e−8
0.90
0.85
0.80
0.75
SMK
E<1
E1−5
E5+
Non

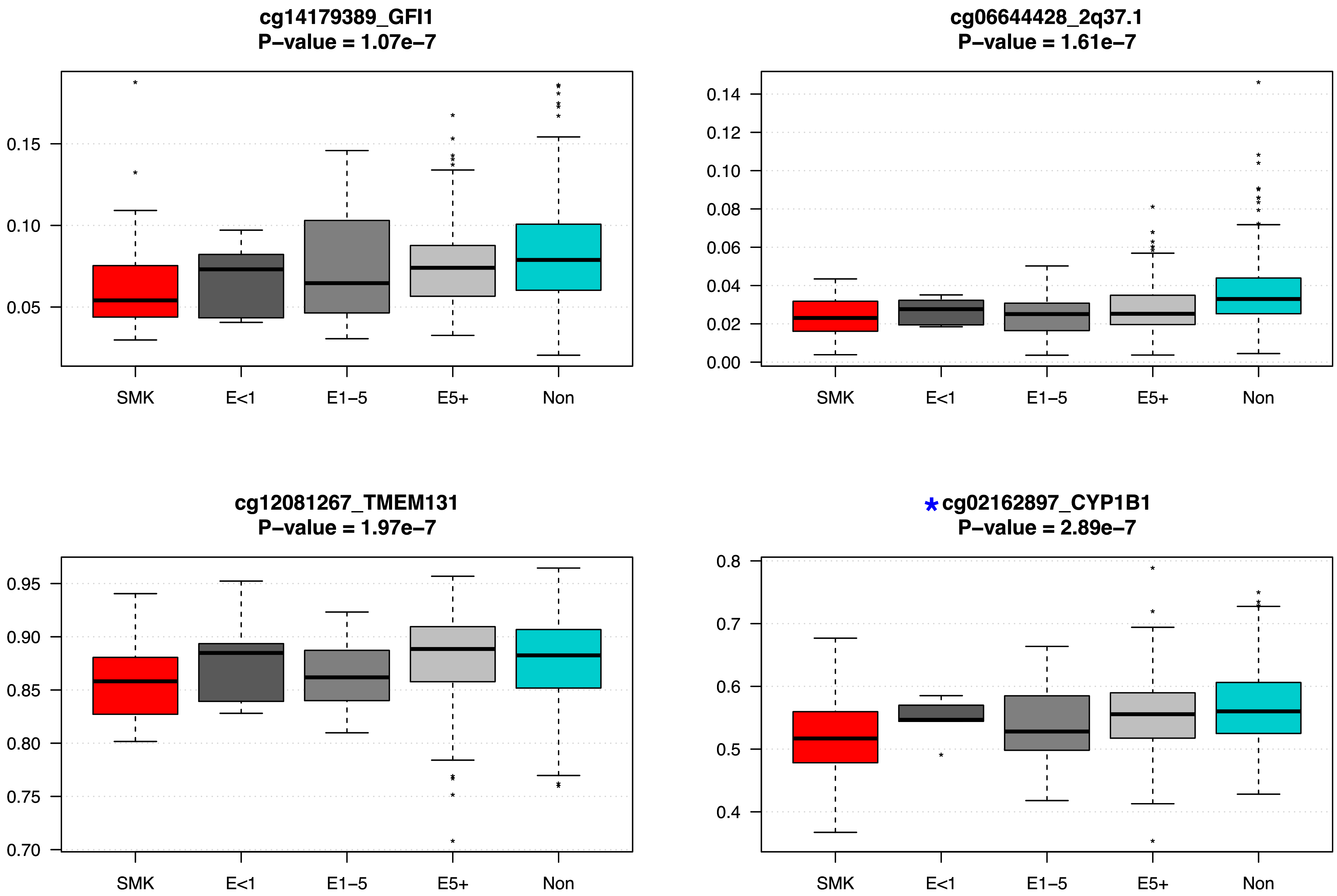
cg14179389_GFI1
P−value = 1.07e−7
0.15
0.10
0.05
SMK
E<1
E1−5
E5+
Non
cg06644428_2q37.1
P−value = 1.61e−7
0.14
0.12
0.10
0.08
0.06
0.04
0.02
0.00
SMK
E<1
E1−5
E5+
Non
cg12081267_TMEM131
P−value = 1.97e−7
0.95
0.90
0.85
0.80
0.75
0.70
SMK
E<1
E1−5
E5+
Non
*cg02162897_CYP1B1
P−value = 2.89e−7
0.8
0.7
0.6
0.5
0.4
SMK
E<1
E1−5
E5+
Non

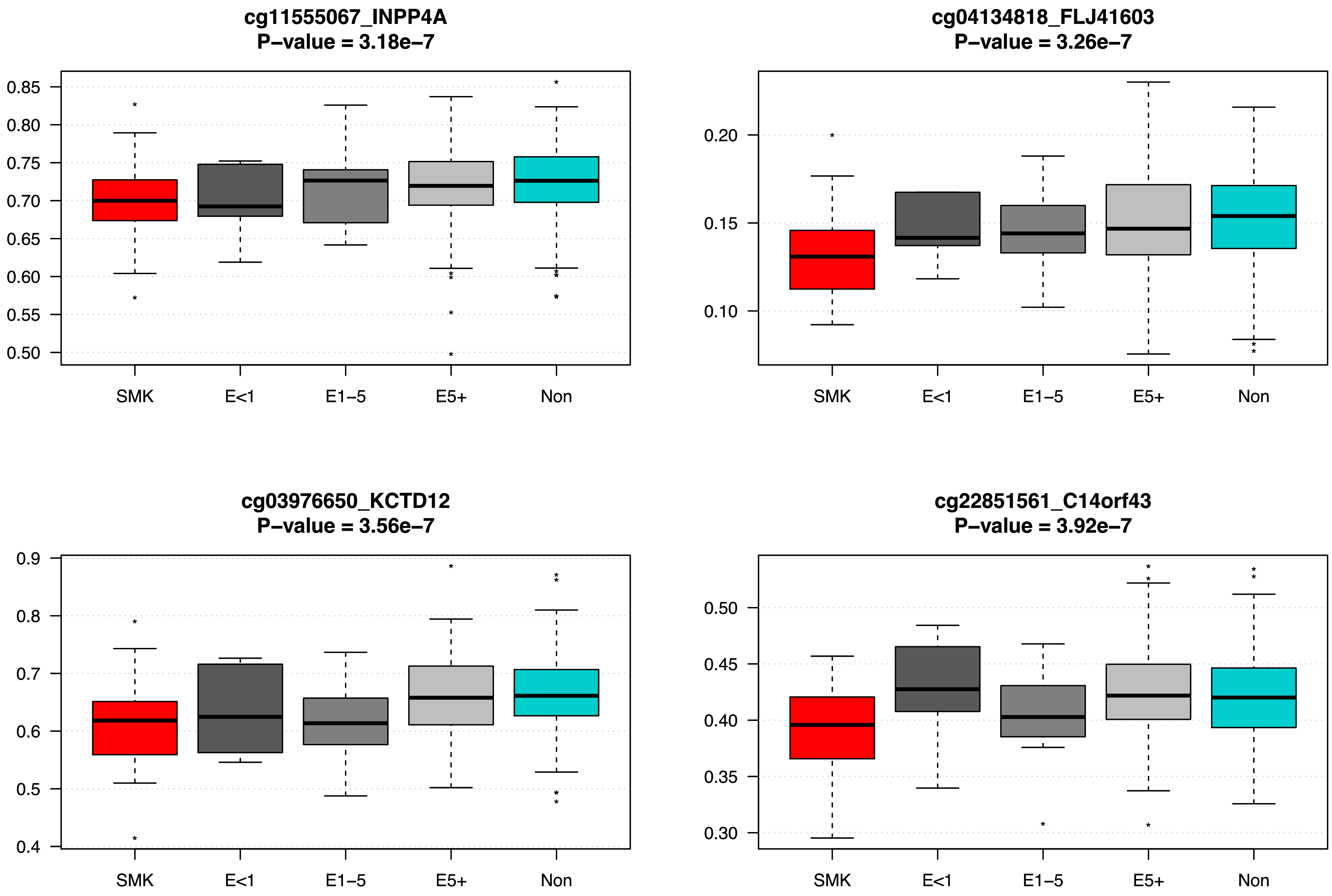
cg11555067_INPP4A
P−value = 3.18e−7
0.85
0.80
0.75
0.70
0.65
0.60
0.55
0.50
SMK
E<1
E1−5
E5+
Non
cg04134818_FLJ41603
P−value = 3.26e−7
0.20
0.15
0.10
SMK
E<1
E1−5
E5+
Non
cg03976650_KCTD12
P−value = 3.56e−7
0.9
0.8
0.7
0.6
0.5
0.4
SMK
E<1
E1−5
E5+
Non
cg22851561_C14orf43
P−value = 3.92e−7
0.50
0.45
0.40
0.35
0.30
SMK
E<1
E1−5
E5+
Non

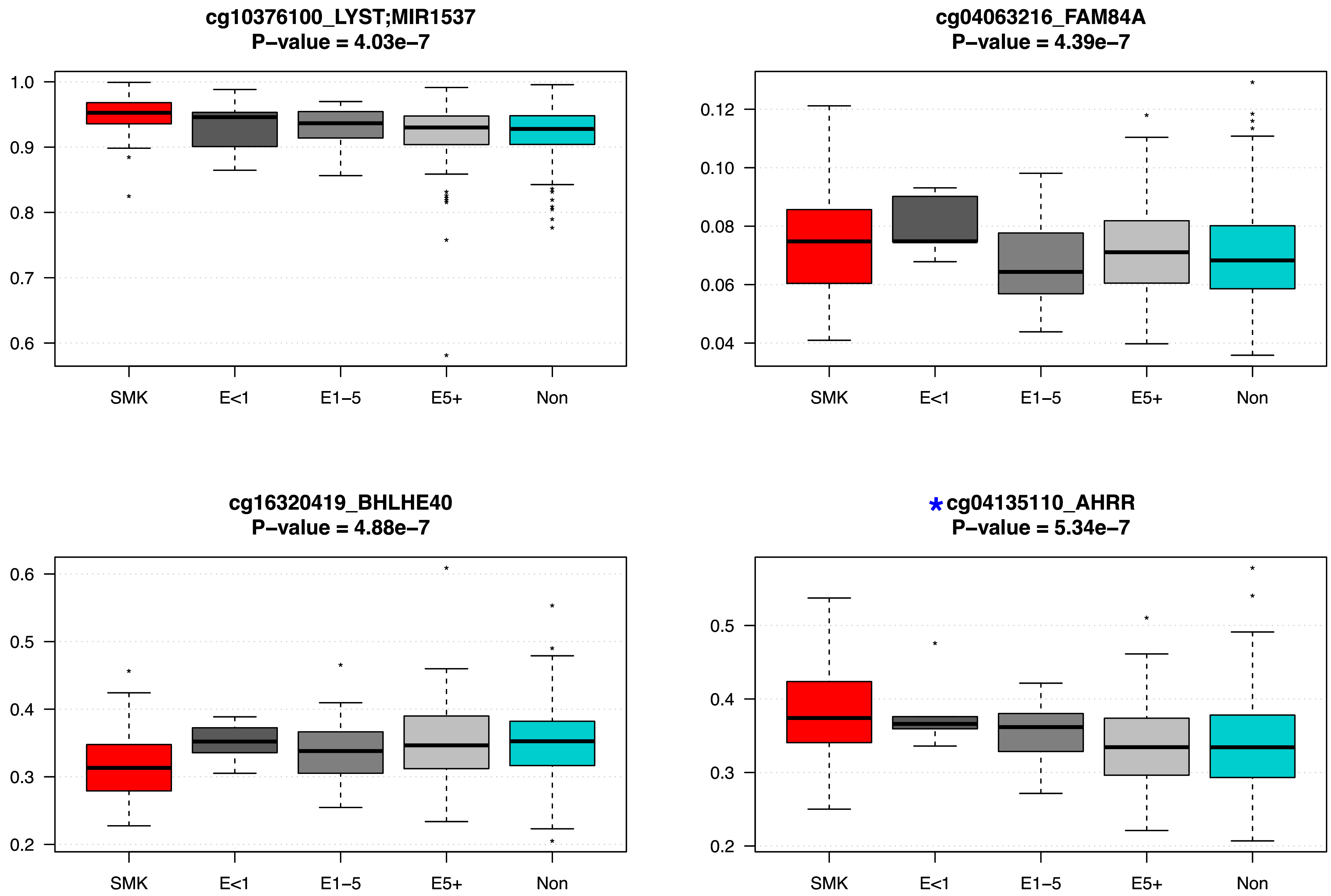
cg10376100_LYST;MIR1537
P−value = 4.03e−7
1.0
0.9
0.8
0.7
0.6
SMK
E<1
E1−5
E5+
Non
cg04063216_FAM84A
P−value = 4.39e−7
0.12
0.10
0.08
0.06
0.04
SMK
E<1
E1−5
E5+
Non
cg16320419_BHLHE40
P−value = 4.88e−7
0.6
0.5
0.4
0.3
0.2
SMK
E<1
E1−5
E5+
Non
*cg04135110_AHRR
P−value = 5.34e−7
0.5
0.4
0.3
0.2
SMK
E<1
E1−5
E5+
Non

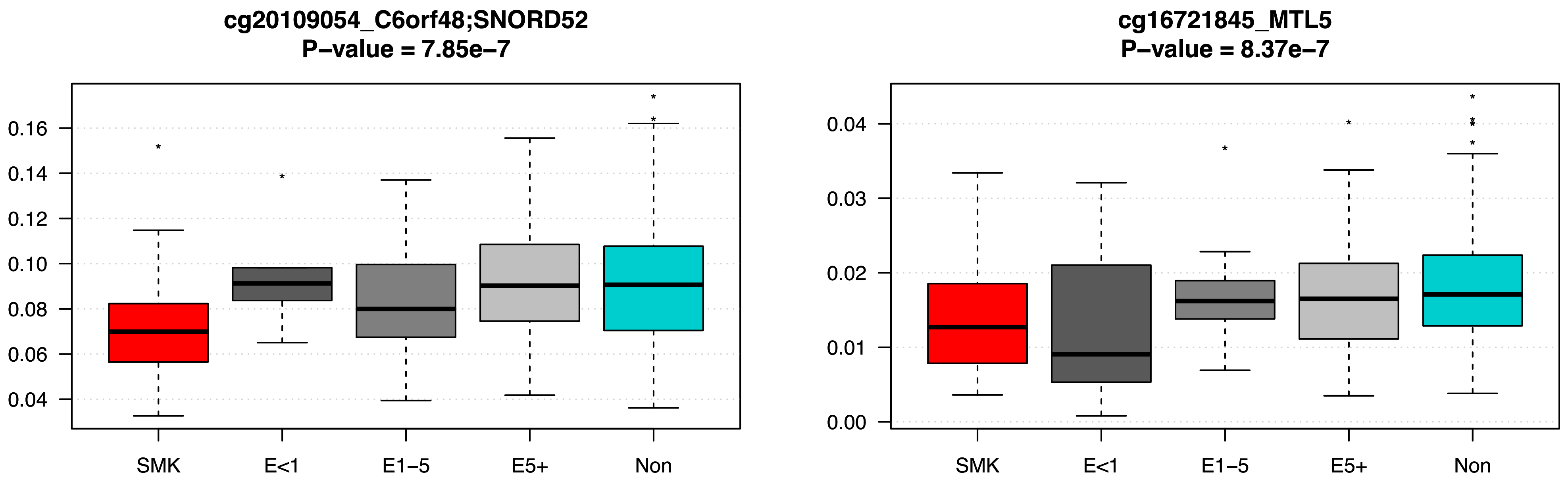
cg20109054_C6orf48;SNORD52
P−value = 7.85e−7
0.16
0.14
0.12
0.10
0.08
0.06
0.04
SMK
E<1
E1−5
E5+
Non
cg16721845_MTL5
P−value = 8.37e−7
0.04
0.03
0.02
0.01
0.00
SMK
E<1
E1−5
E5+
Non

Figure S3

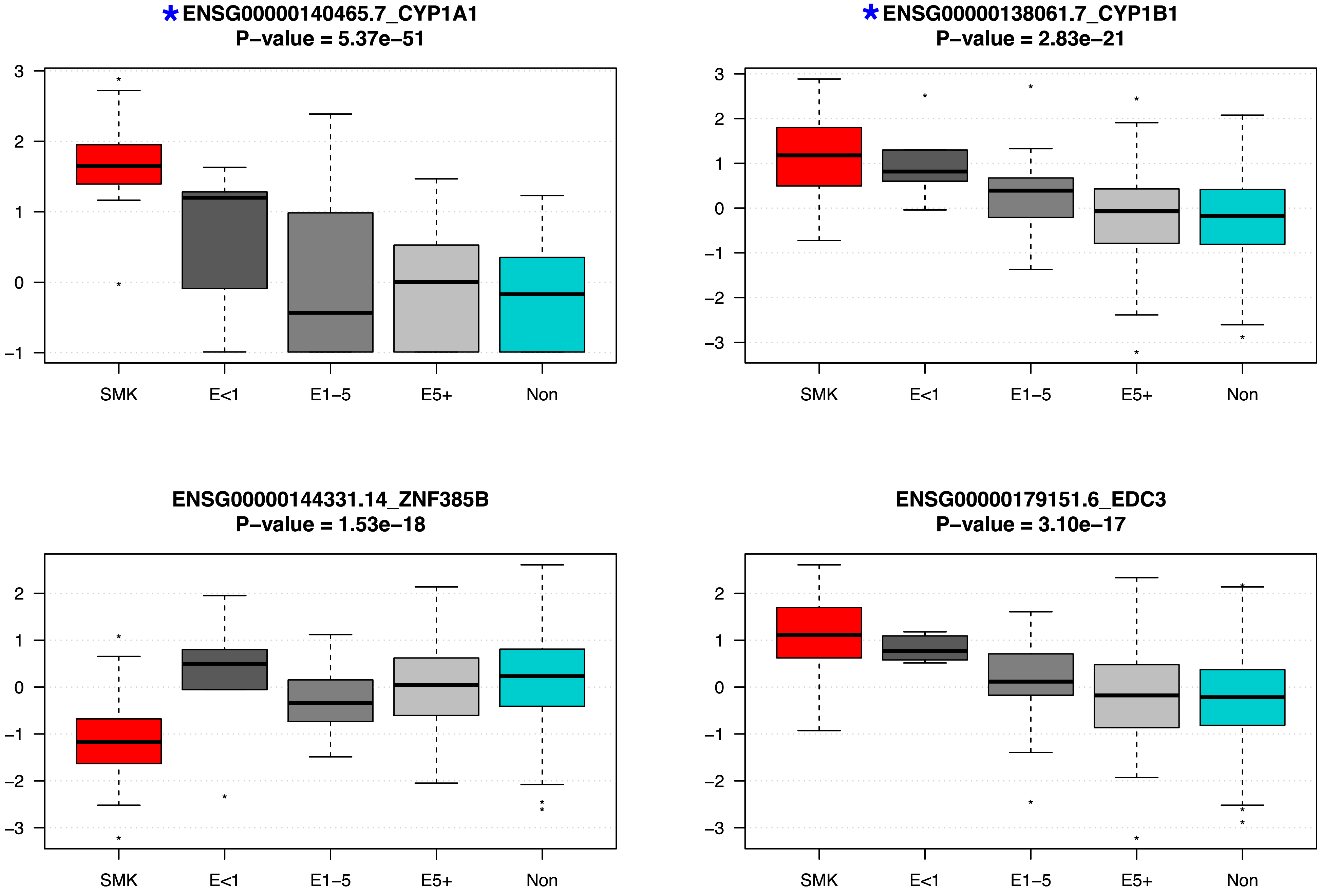

*ENSG00000140465.7_CYP1A1
P−value = 5.37e−51
3
2
1
0
−1
SMK
E<1
E1−5
E5+
Non
*ENSG00000138061.7_CYP1B1
P−value = 2.83e−21
3
2
1
0
−1
−2
−3
SMK
E<1
E1−5
E5+
Non
ENSG00000144331.14_ZNF385B
P−value = 1.53e−18
2
1
0
−1
−2
−3
SMK
E<1
E1−5
E5+
Non
ENSG00000179151.6_EDC3
P−value = 3.10e−17
2
1
0
−1
−2
−3
SMK
E<1
E1−5
E5+
Non

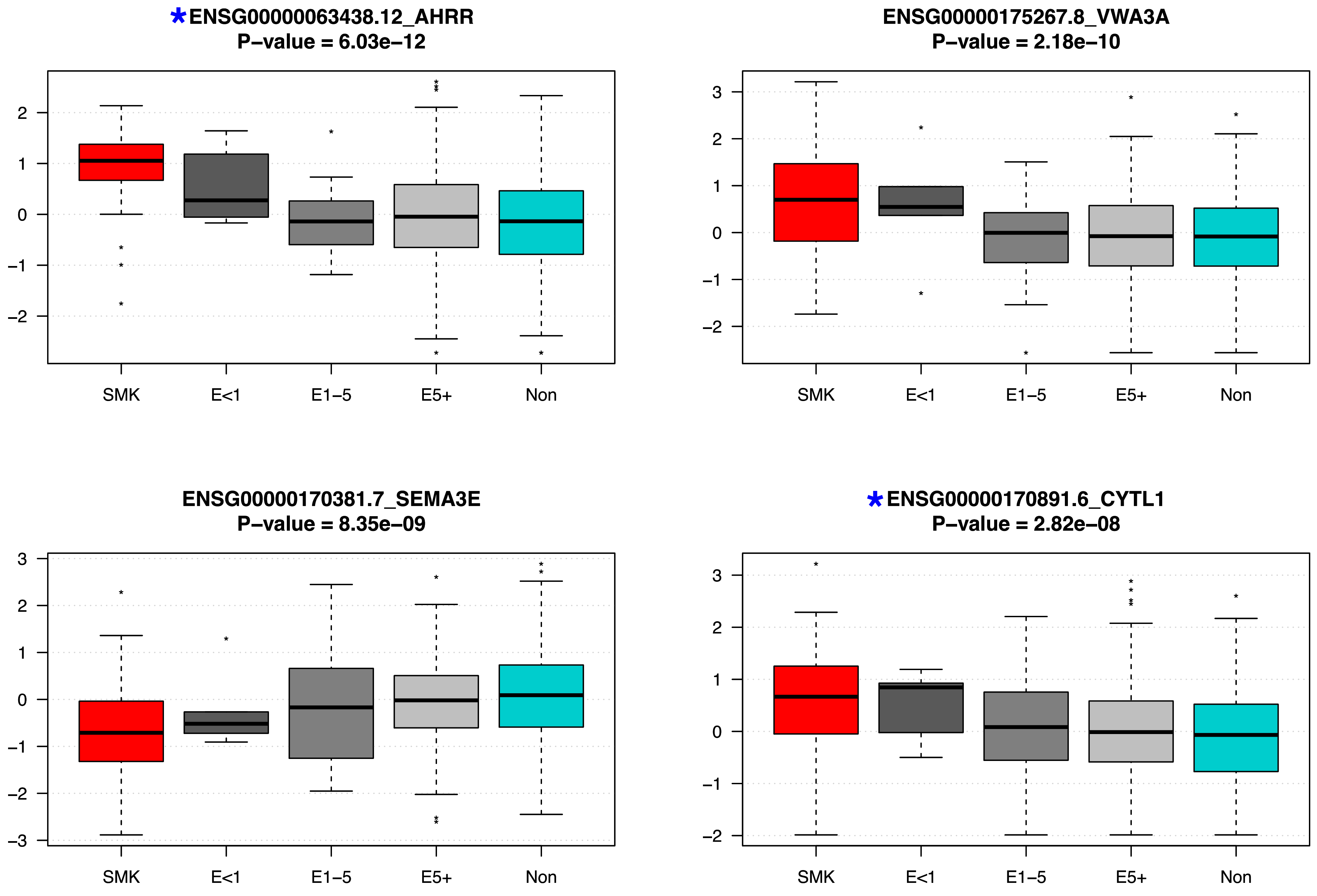
*ENSG00000063438.12_AHRR
P–value = 6.03e–12
ENSG00000175267.8_VWA3A
P–value = 2.18e–10
ENSG00000170381.7_SEMA3E
P–value = 8.35e–09
*ENSG00000170891.6_CYTL1
P–value = 2.82e–08
SMK
E<1
E1–5
E5+
Non

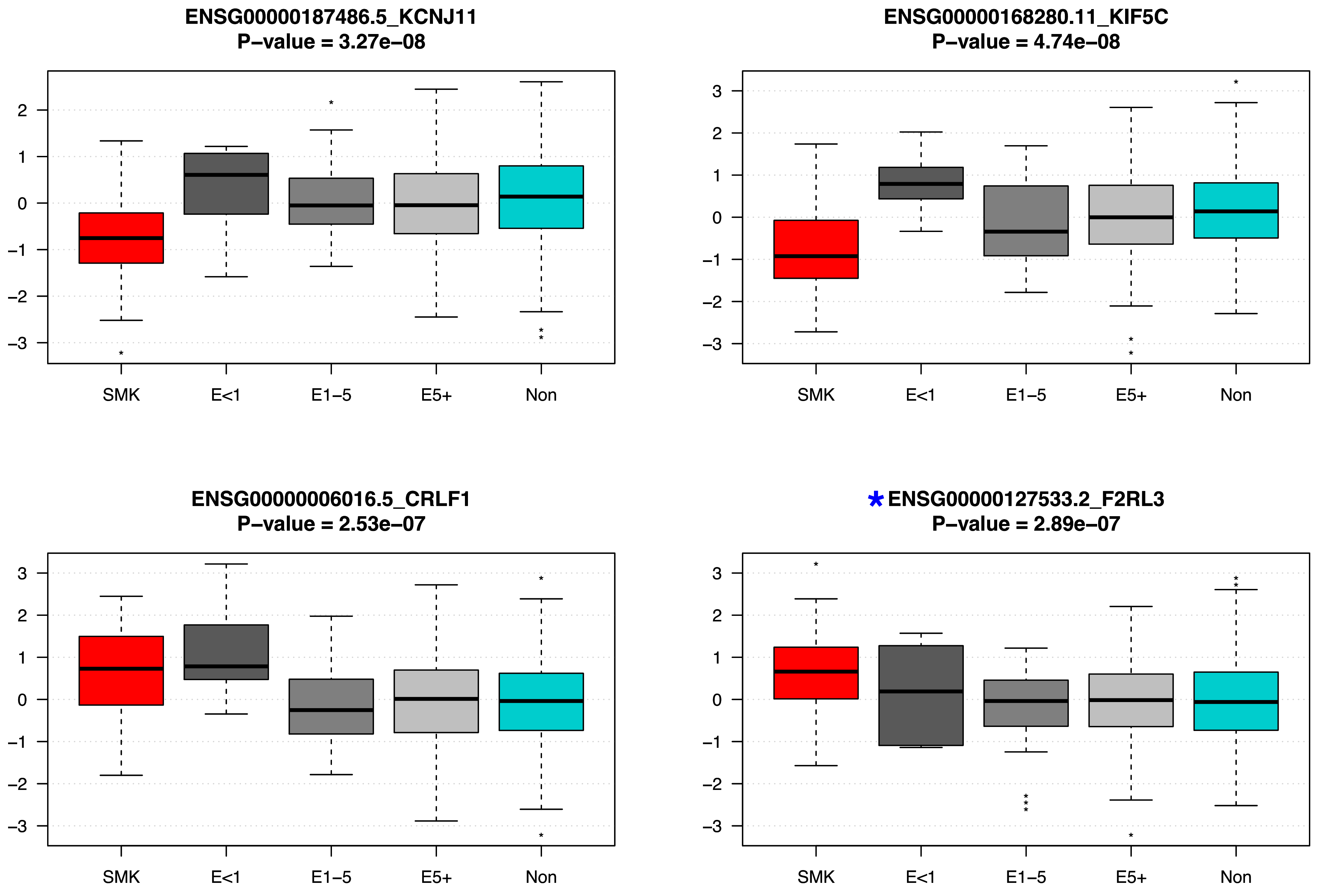

ENSG00000187486.5_KCNJ11
P−value = 3.27e−08
ENSG00000168280.11_KIF5C
P−value = 4.74e−08
ENSG00000006016.5_CRLF1
P−value = 2.53e−07
*ENSG00000127533.2_F2RL3
P−value = 2.89e−07
SMK
E<1
E1−5
E5+
Non

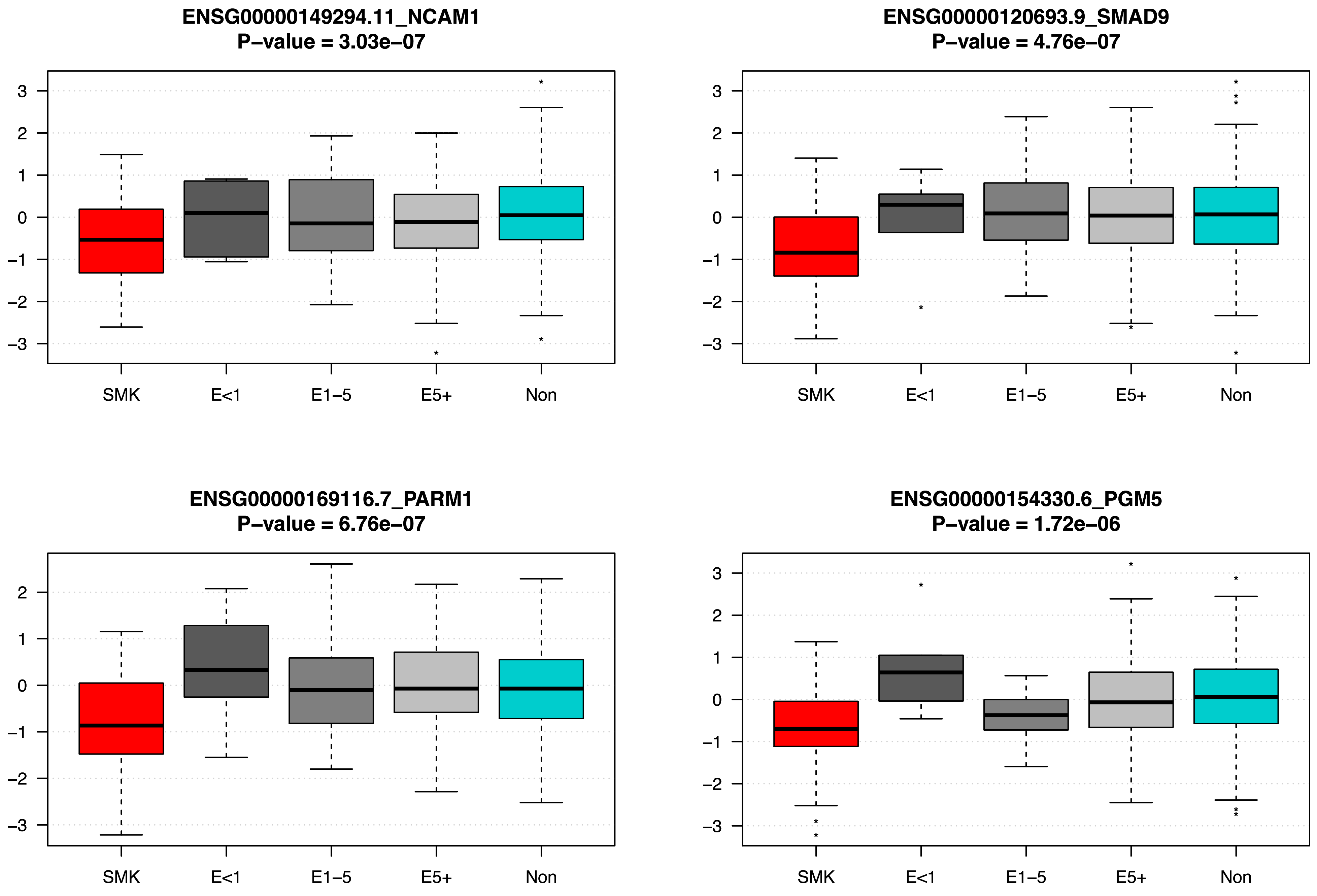
ENSG00000149294.11_NCAM1
P−value = 3.03e−07
ENSG00000120693.9_SMAD9
P−value = 4.76e−07
ENSG00000169116.7_PARM1
P−value = 6.76e−07
ENSG00000154330.6_PGM5
P−value = 1.72e−06
SMK
E<1
E1−5
E5+
Non

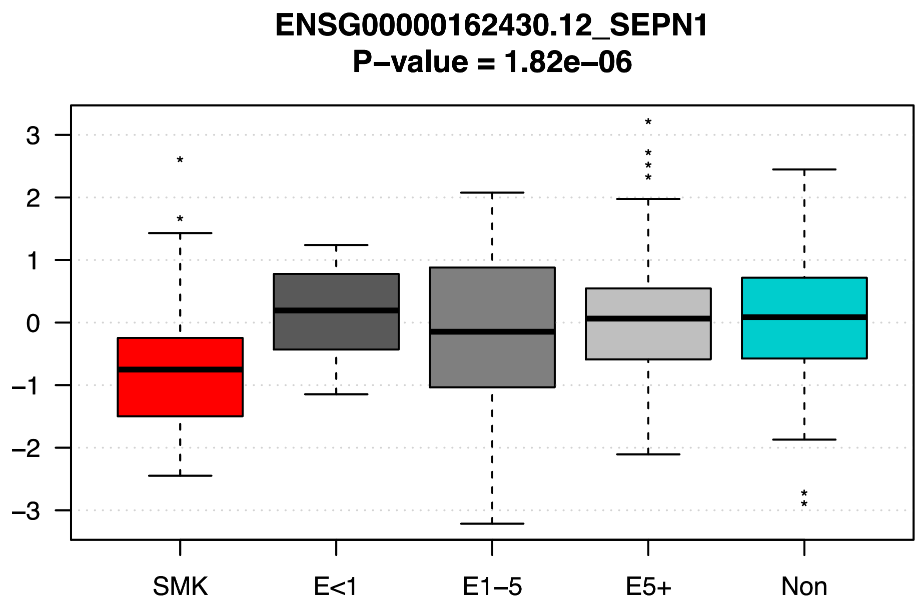
ENSG00000162430.12_SEPN1
P−value = 1.82e−06
3
2
1
0
−1
−2
−3
SMK
E<1
E1−5
E5+
Non

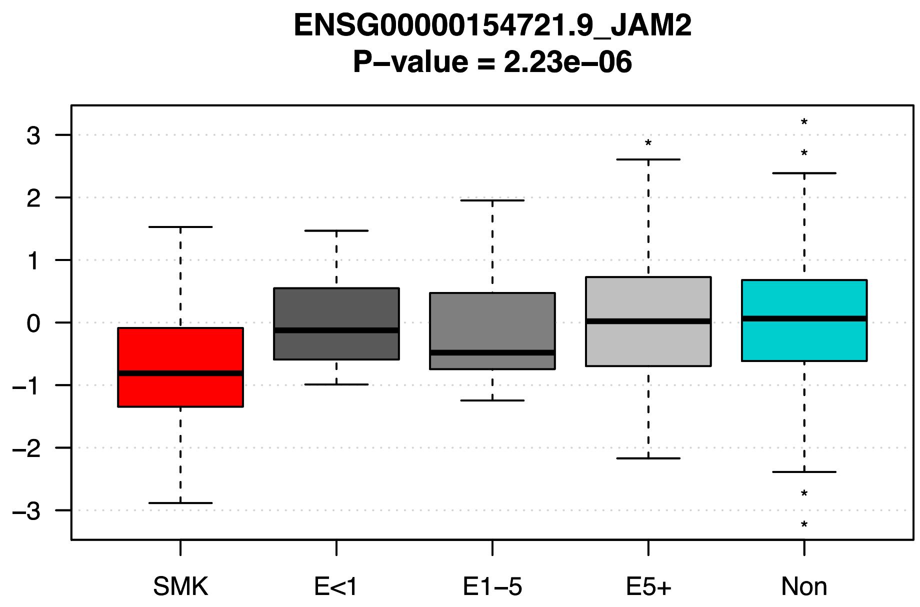
ENSG00000154721.9_JAM2
P−value = 2.23e−06
3
2
1
0
−1
−2
−3
SMK
E<1
E1−5
E5+
Non

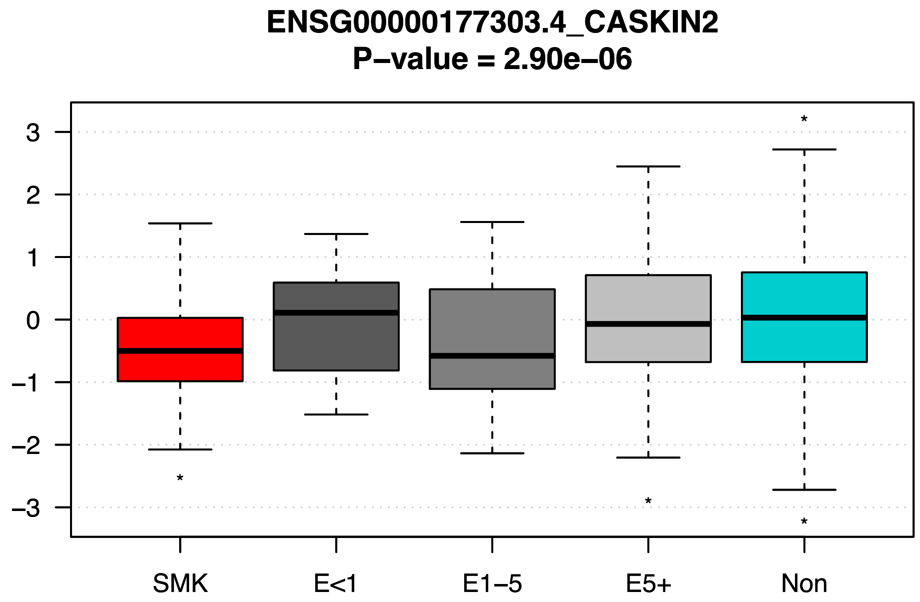
ENSG00000177303.4_CASKIN2
P−value = 2.90e−06
3
2
1
0
−1
−2
−3
SMK
E<1
E1−5
E5+
Non

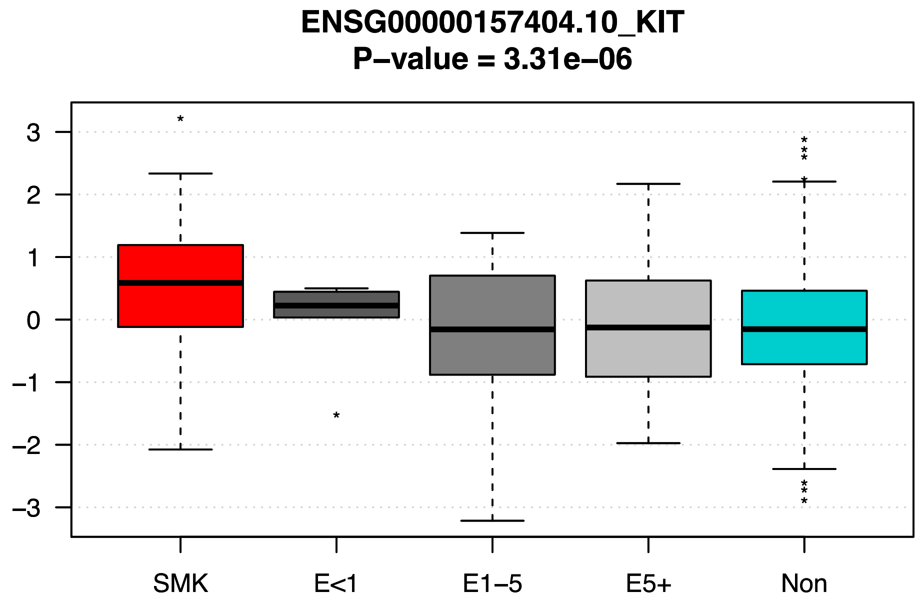
ENSG00000157404.10_KIT
P−value = 3.31e−06
3
2
1
0
−1
−2
−3
SMK
E<1
E1−5
E5+
Non

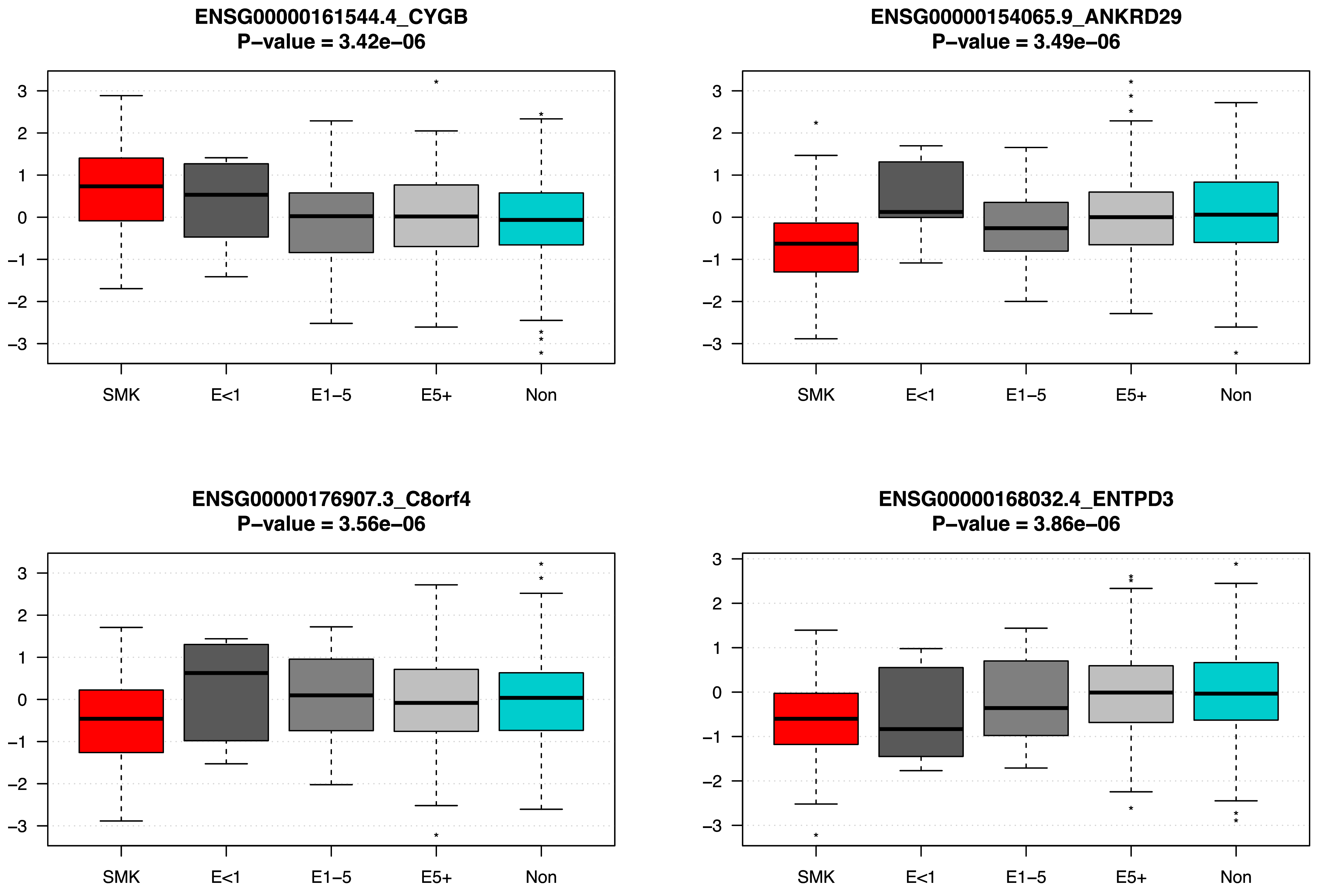
ENSG00000161544.4_CYGB
P−value = 3.42e−06
ENSG00000154065.9_ANKRD29
P−value = 3.49e−06
ENSG00000176907.3_C8orf4
P−value = 3.56e−06
ENSG00000168032.4_ENTPD3
P−value = 3.86e−06
3
2
1
0
−1
−2
−3
SMK
E<1
E1−5
E5+
Non

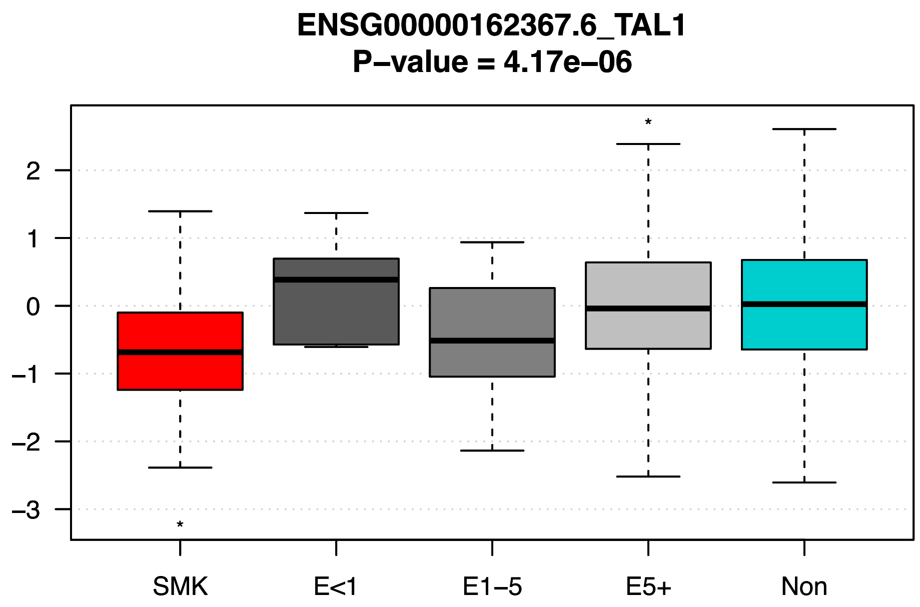
ENSG00000162367.6_TAL1
P−value = 4.17e−06
SMK
E<1
E1−5
E5+
Non

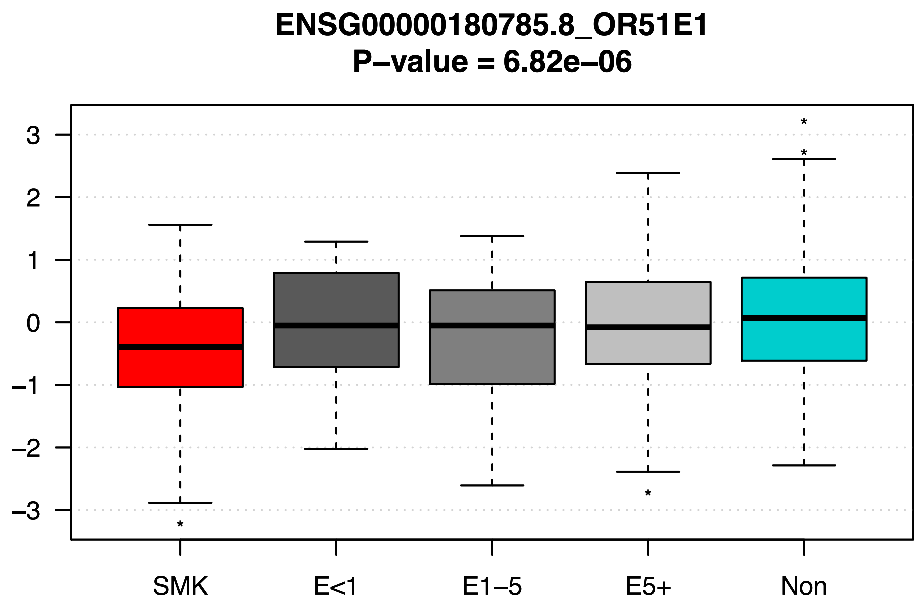
ENSG00000180785.8_OR51E1
P−value = 6.82e−06
SMK
E<1
E1−5
E5+
Non

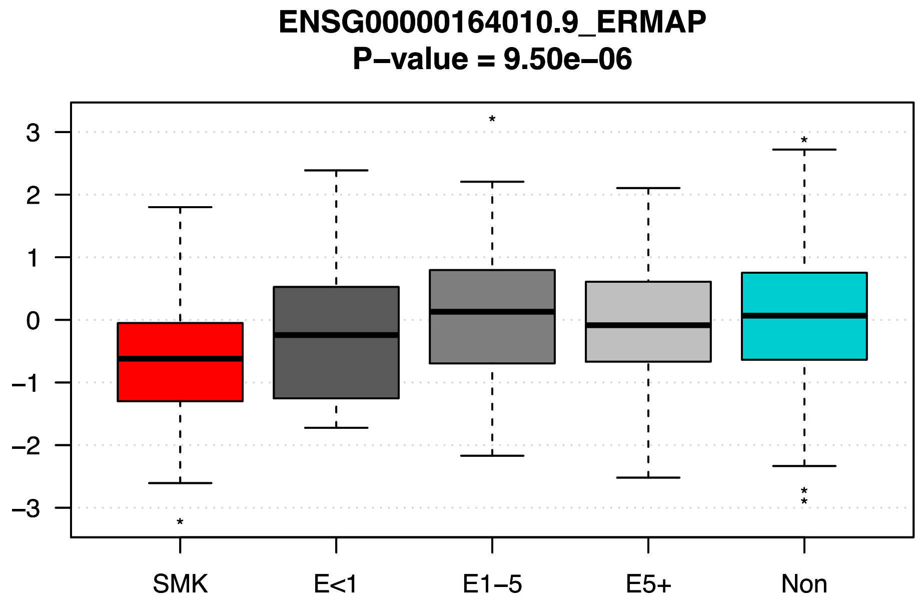
ENSG00000164010.9_ERMAP
P−value = 9.50e−06
SMK
E<1
E1−5
E5+
Non

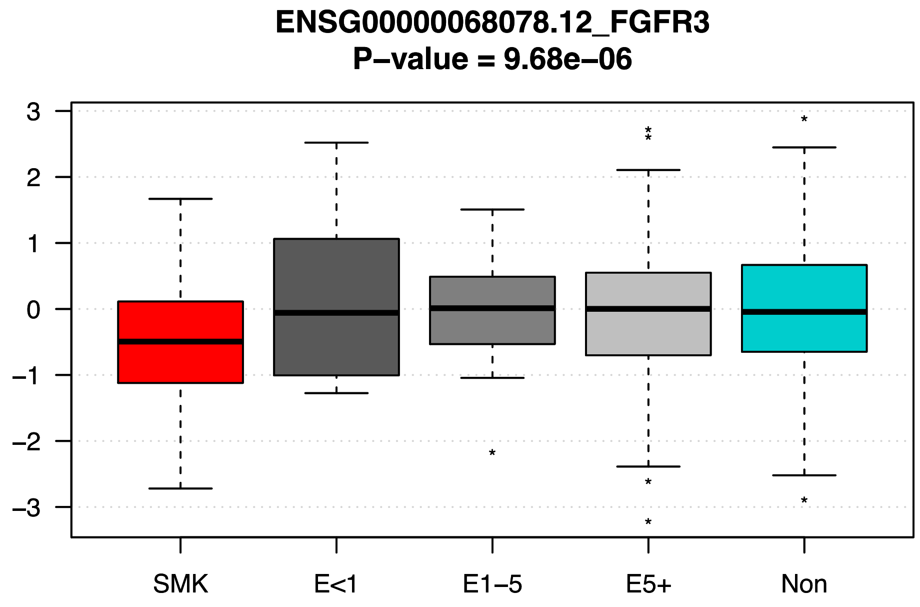
ENSG00000068078.12_FGFR3
P−value = 9.68e−06
SMK
E<1
E1−5
E5+
Non

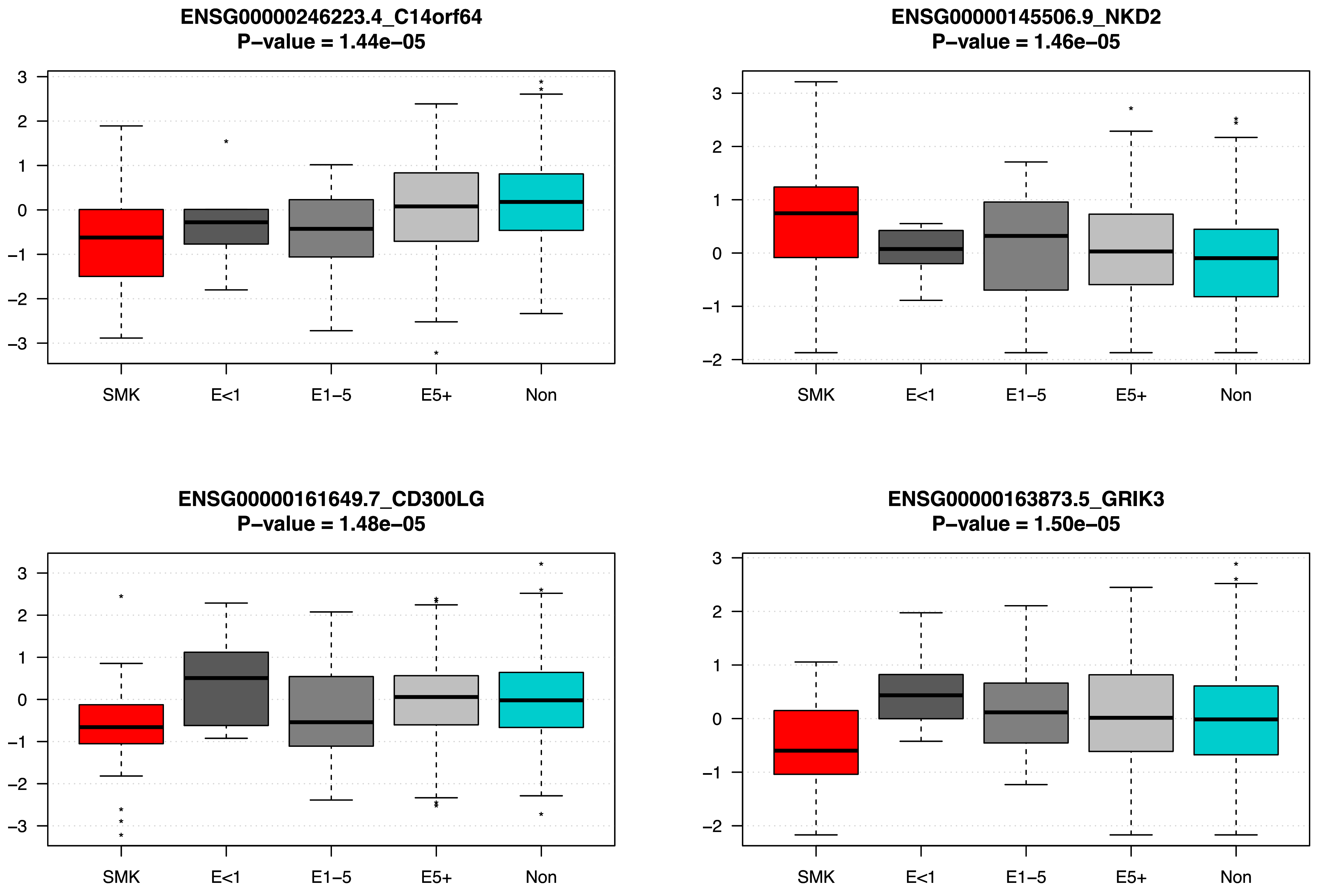
ENSG00000246223.4_C14orf64
P−value = 1.44e−05
ENSG00000145506.9_NKD2
P−value = 1.46e−05
ENSG00000161649.7_CD300LG
P−value = 1.48e−05
ENSG00000163873.5_GRIK3
P−value = 1.50e−05
SMK
E<1
E1−5
E5+
Non
3
2
1
0
−1
−2
−3

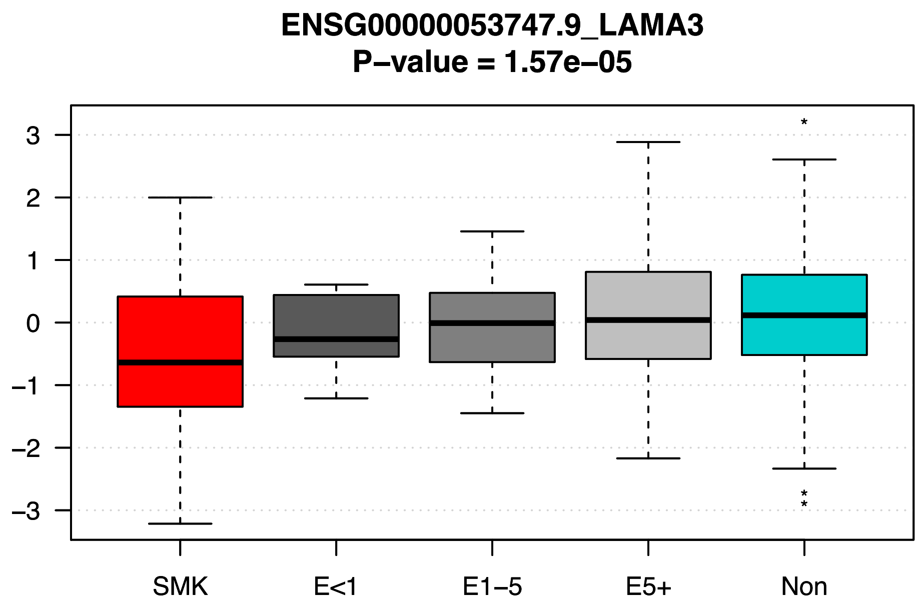
ENSG00000053747.9_LAMA3
P–value = 1.57e–05
3
2
1
0
−1
−2
−3
SMK
E<1
E1–5
E5+
Non

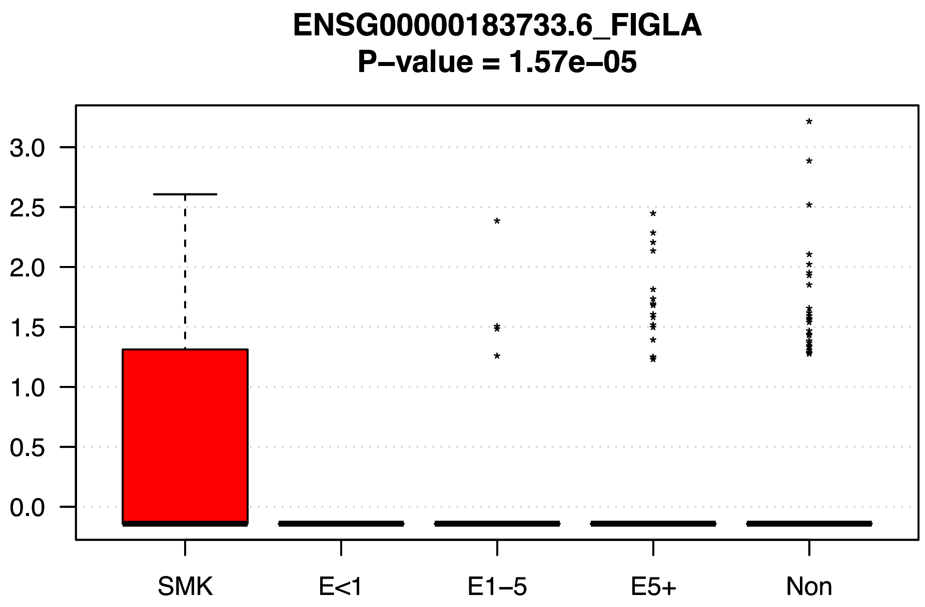
ENSG00000183733.6_FIGLA
P–value = 1.57e–05
3.0
2.5
2.0
1.5
1.0
0.5
0.0
SMK
E<1
E1–5
E5+
Non

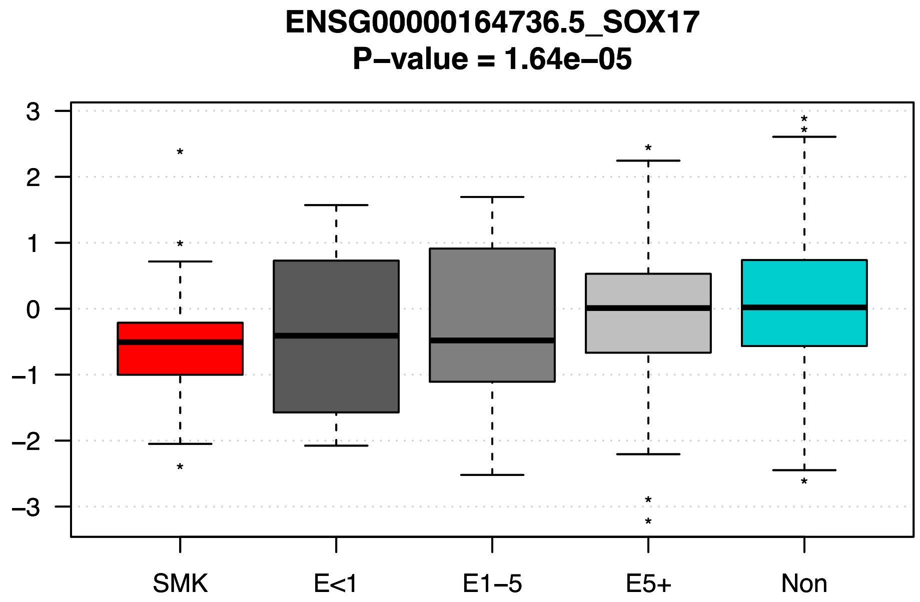
ENSG00000164736.5_SOX17
P–value = 1.64e–05
3
2
1
0
−1
−2
−3
SMK
E<1
E1–5
E5+
Non

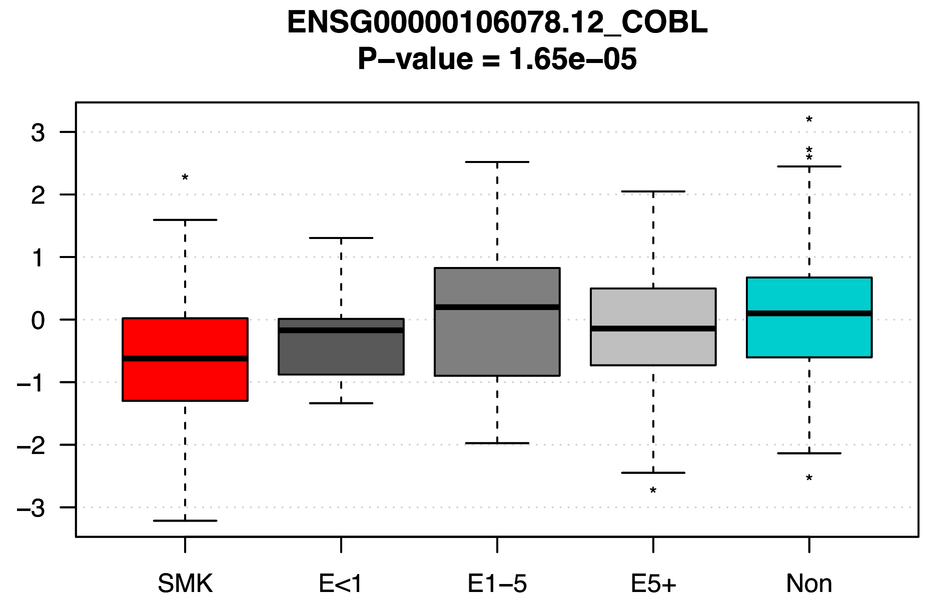
ENSG00000106078.12_COBL
P–value = 1.65e–05
3
2
1
0
−1
−2
−3
SMK
E<1
E1–5
E5+
Non

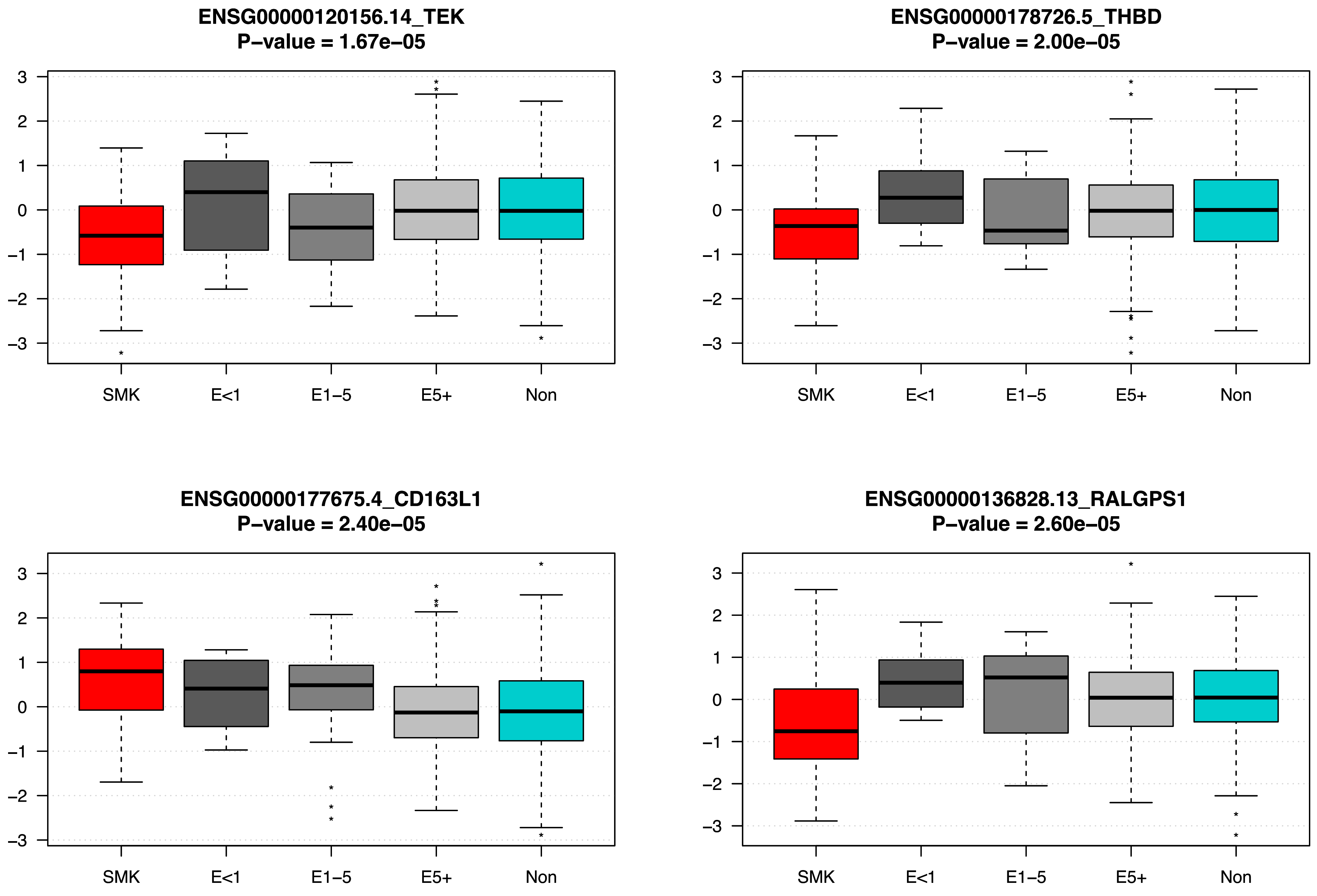
ENSG00000120156.14_TEK
P–value = 1.67e–05
ENSG00000178726.5_THBD
P–value = 2.00e–05
ENSG00000177675.4_CD163L1
P–value = 2.40e–05
ENSG00000136828.13_RALGPS1
P–value = 2.60e–05
3
2
1
0
−1
−2
−3
SMK
E<1
E1–5
E5+
Non

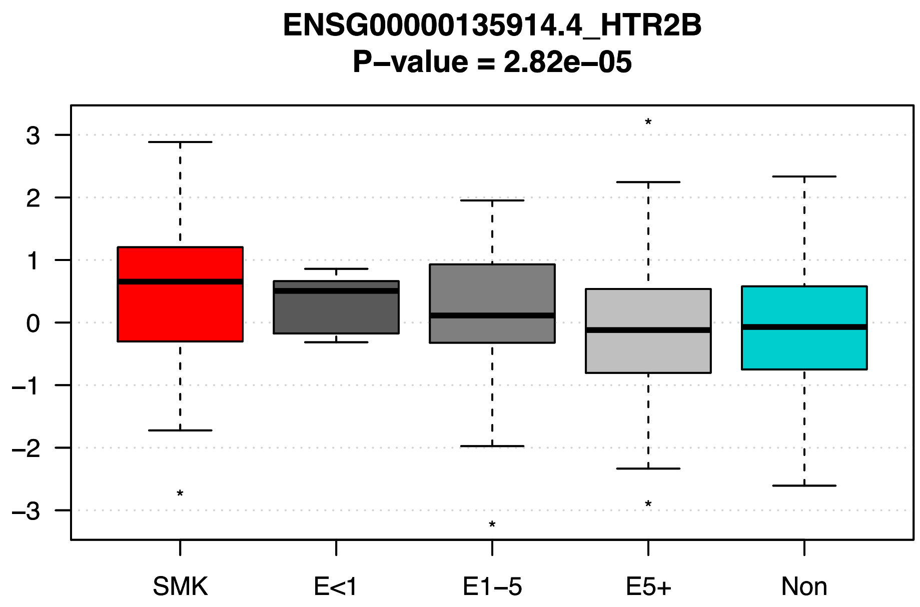
ENSG00000135914.4_HTR2B
P–value = 2.82e–05
3
2
1
0
–1
–2
–3
SMK
E<1
E1–5
E5+
Non

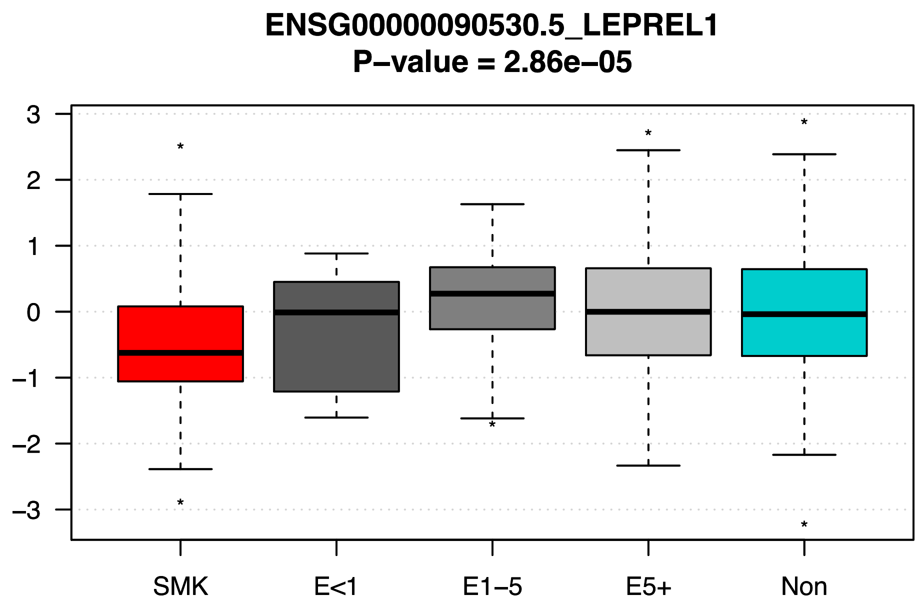
ENSG00000090530.5_LEPREL1
P–value = 2.86e–05
3
2
1
0
–1
–2
–3
SMK
E<1
E1–5
E5+
Non

Figure S4

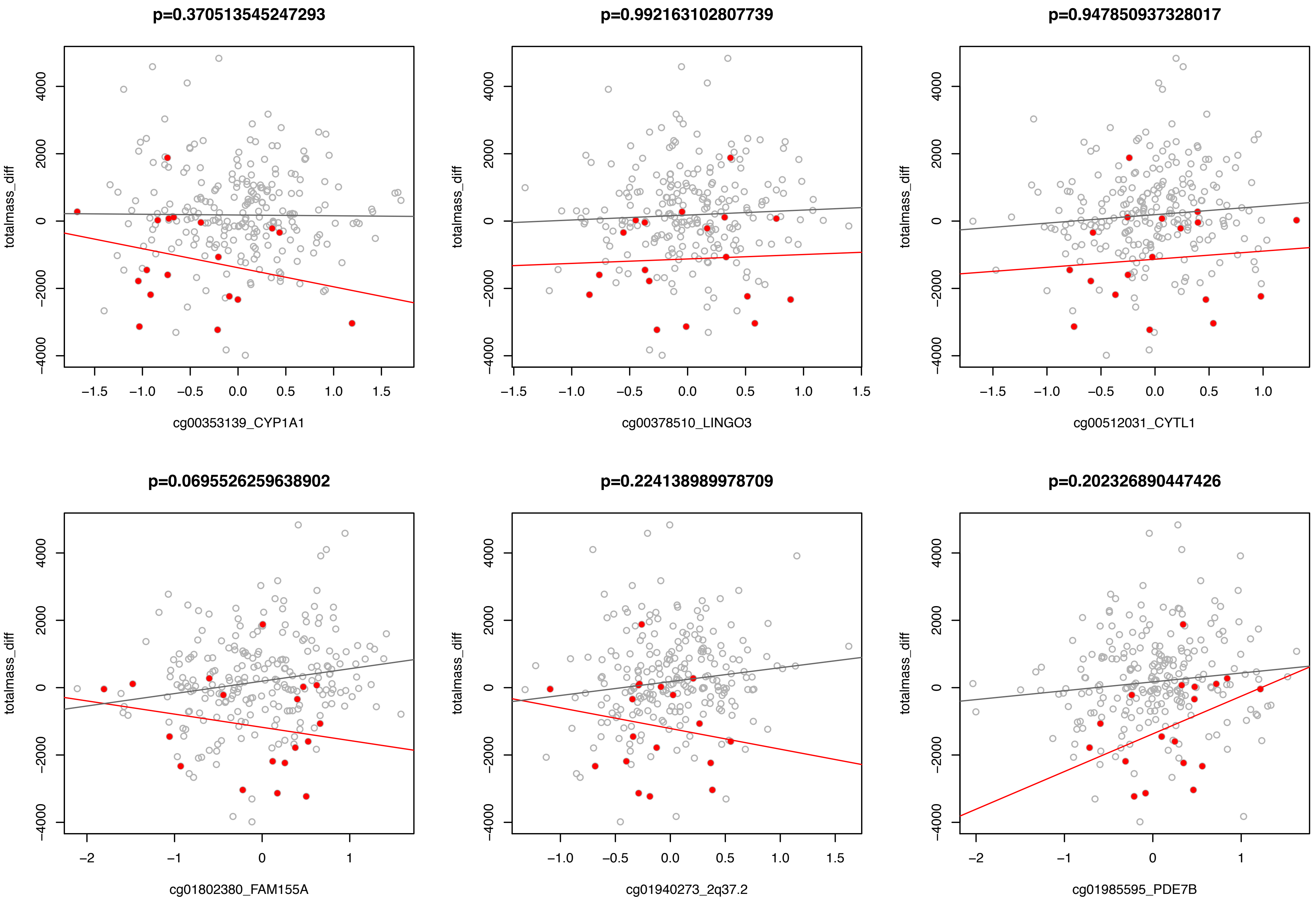

p=0.370513545247293
p=0.992163102807739
p=0.947850937328017
p=0.0695526259638902
p=0.224138989978709
p=0.202326890447426
totalmass_diff
4000
2000
0
−2000
−4000
−1.5
−1.0
−0.5
0.0
0.5
1.0
1.5
−2
−1
0
1
cg00353139_CYP1A1
cg00378510_LINGO3
cg00512031_CYTL1
cg01802380_FAM155A
cg01940273_2q37.2
cg01985595_PDE7B

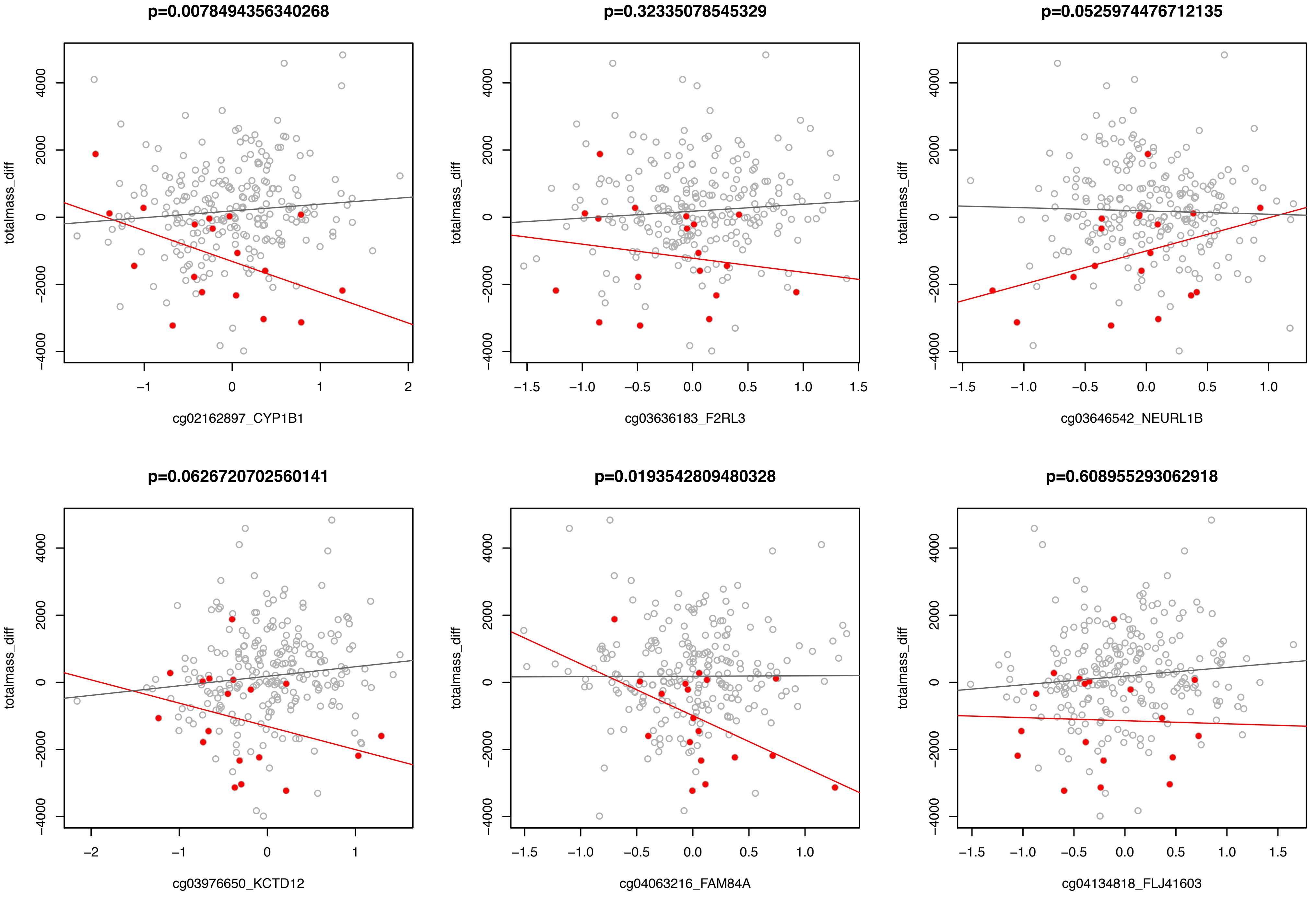
p=0.0078494356340268
p=0.32335078545329
p=0.0525974476712135
p=0.0626720702560141
p=0.0193542809480328
p=0.608955293062918
totalmass_diff
cg02162897_CYP1B1
cg03636183_F2RL3
cg03646542_NEURL1B
cg03976650_KCTD12
cg04063216_FAM84A
cg04134818_FLJ41603

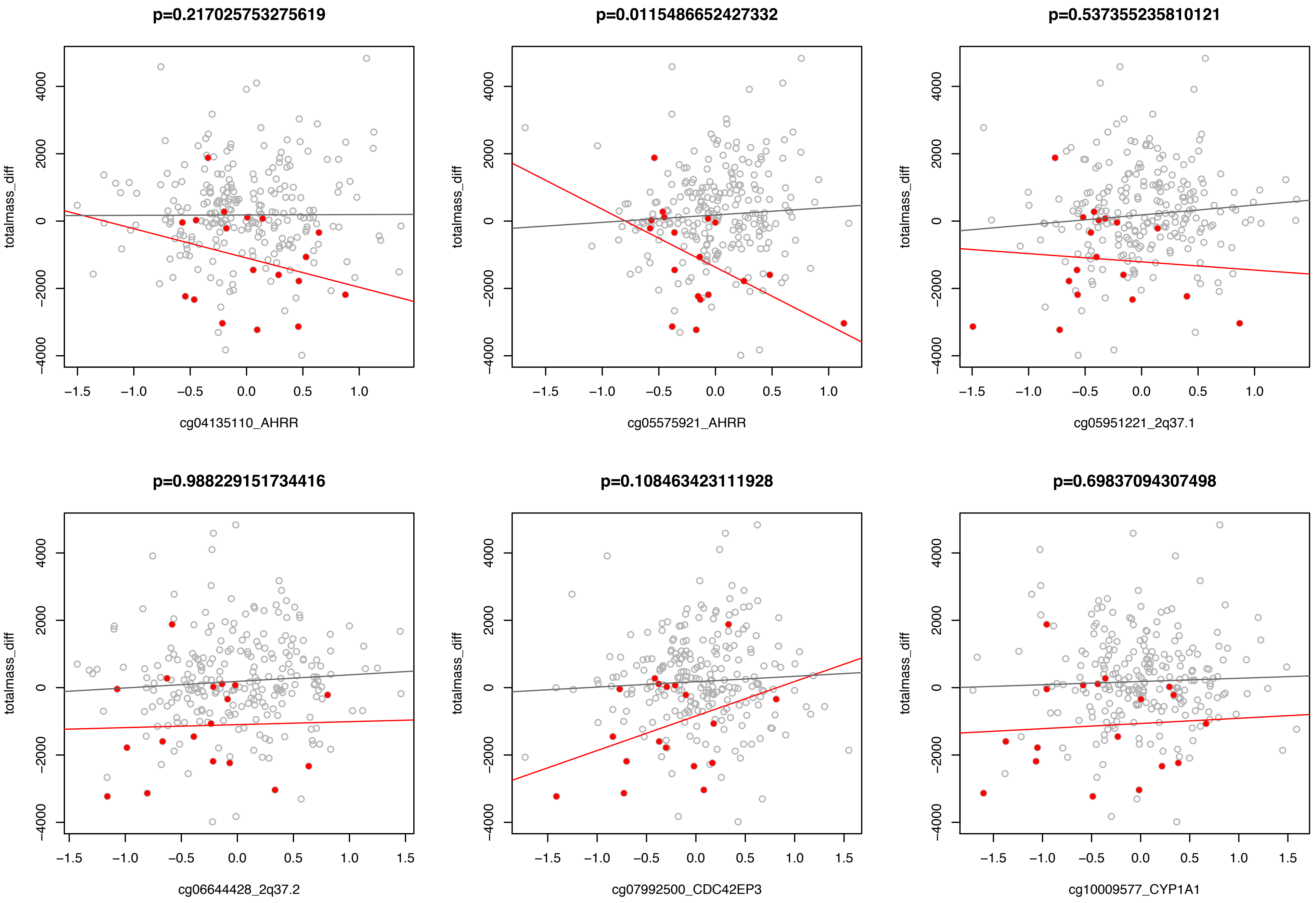
p=0.217025753275619
cg04135110_AHRR
p=0.0115486652427332
cg05575921_AHRR
p=0.537355235810121
cg05951221_2q37.1
p=0.988229151734416
cg06644428_2q37.2
p=0.108463423111928
cg07992500_CDC42EP3
p=0.69837094307498
cg10009577_CYP1A1
totalmass_diff

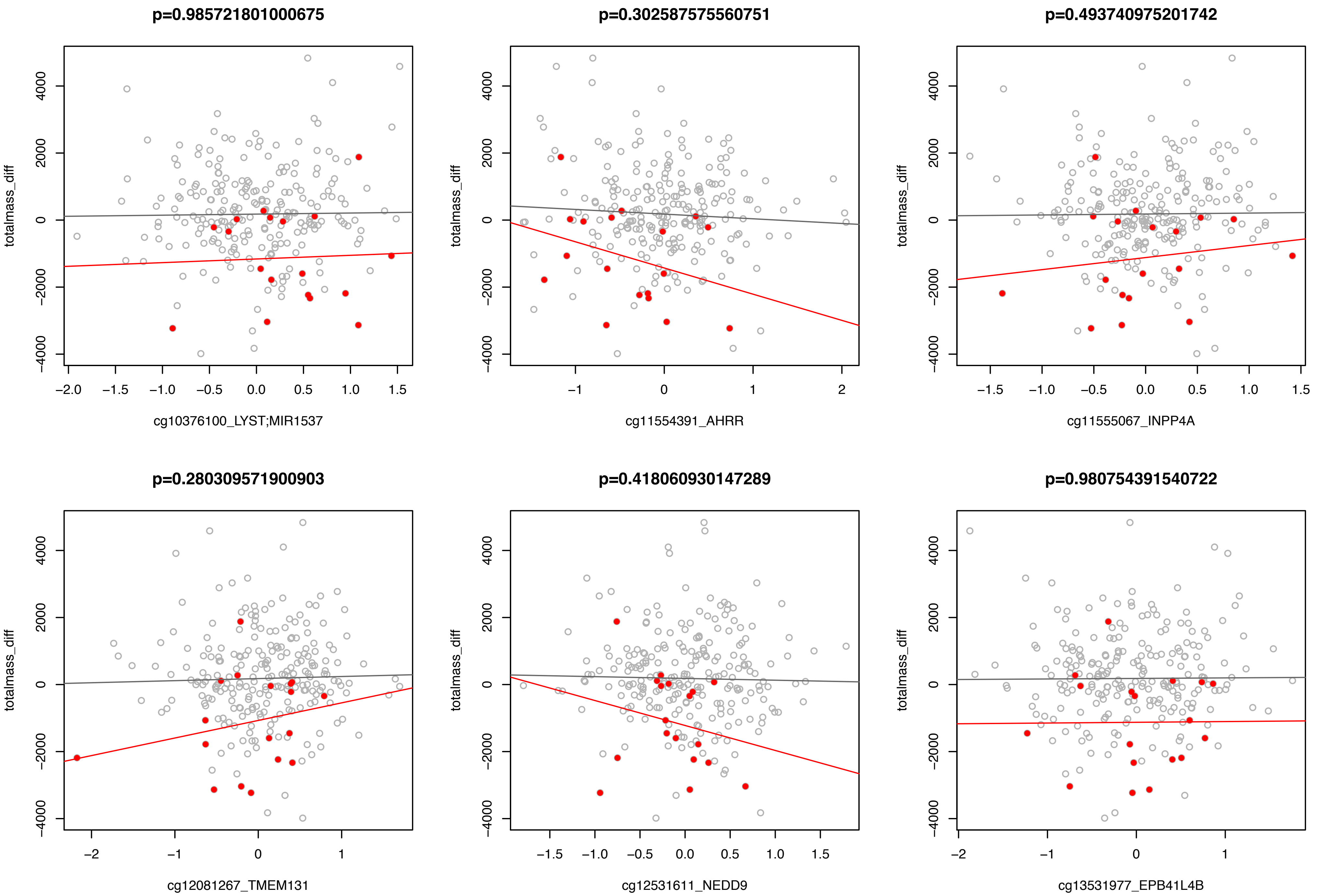
p=0.985721801000675
totalmass_diff
cg10376100_LYST;MIR1537
p=0.302587575560751
totalmass_diff
cg11554391_AHRR
p=0.493740975201742
totalmass_diff
cg11555067_INPP4A
p=0.280309571900903
totalmass_diff
cg12081267_TMEM131
p=0.418060930147289
totalmass_diff
cg12531611_NEDD9
p=0.980754391540722
totalmass_diff
cg13531977_EPB41L4B

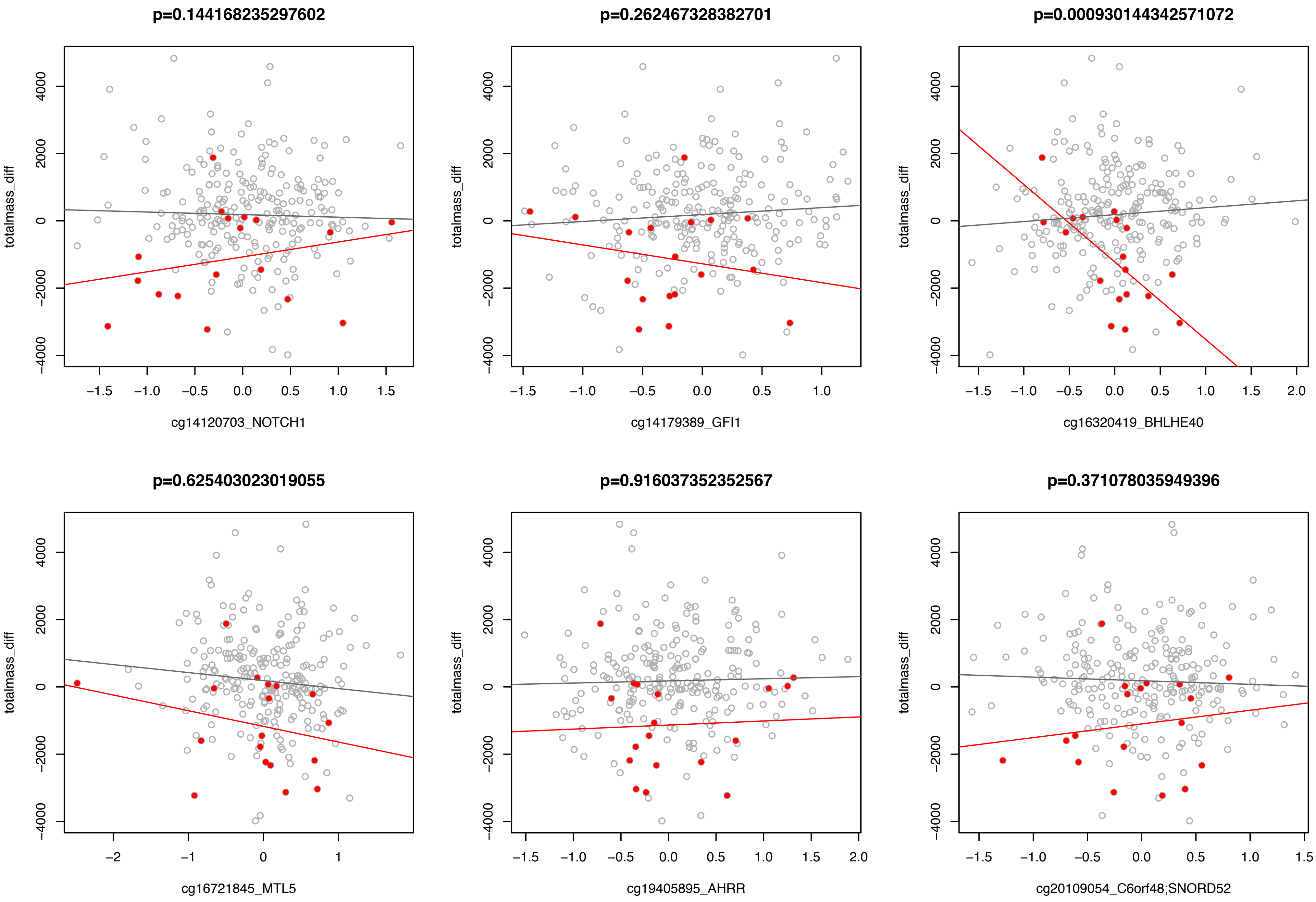
p=0.144168235297602
p=0.262467328382701
p=0.000930144342571072
p=0.625403023019055
p=0.916037352352567
p=0.371078035949396
totalmass_diff
cg14120703_NOTCH1
cg14179389_GFI1
cg16320419_BHLHE40
cg16721845_MTL5
cg19405895_AHRR
cg20109054_C6orf48;SNORD52

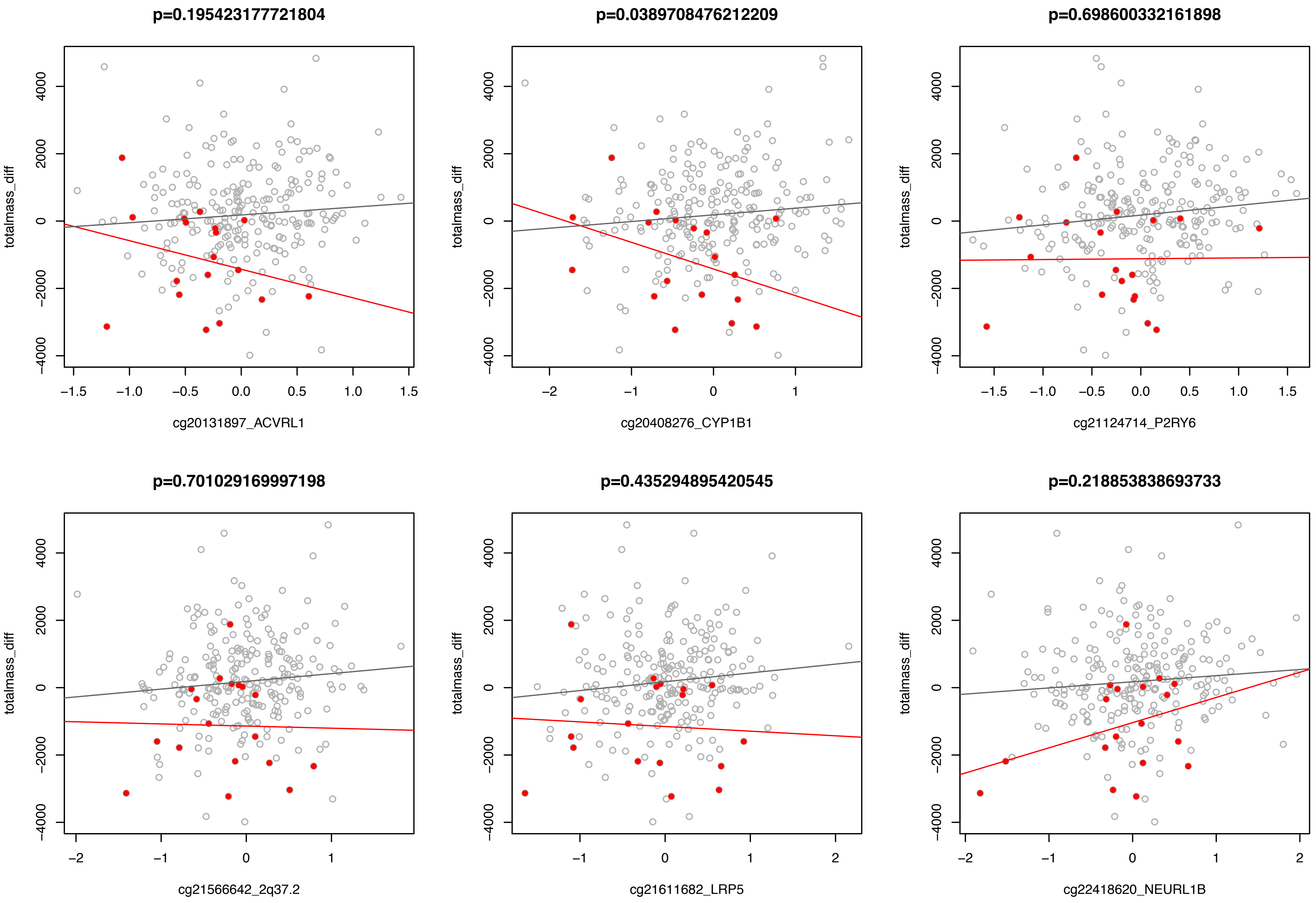
p=0.195423177721804
totalmass_diff
cg20131897_ACVRL1
p=0.0389708476212209
totalmass_diff
cg20408276_CYP1B1
p=0.698600332161898
totalmass_diff
cg21124714_P2RY6
p=0.701029169997198
totalmass_diff
cg21566642_2q37.2
p=0.435294895420545
totalmass_diff
cg21611682_LRP5
p=0.218853838693733
totalmass_diff
cg22418620_NEURL1B

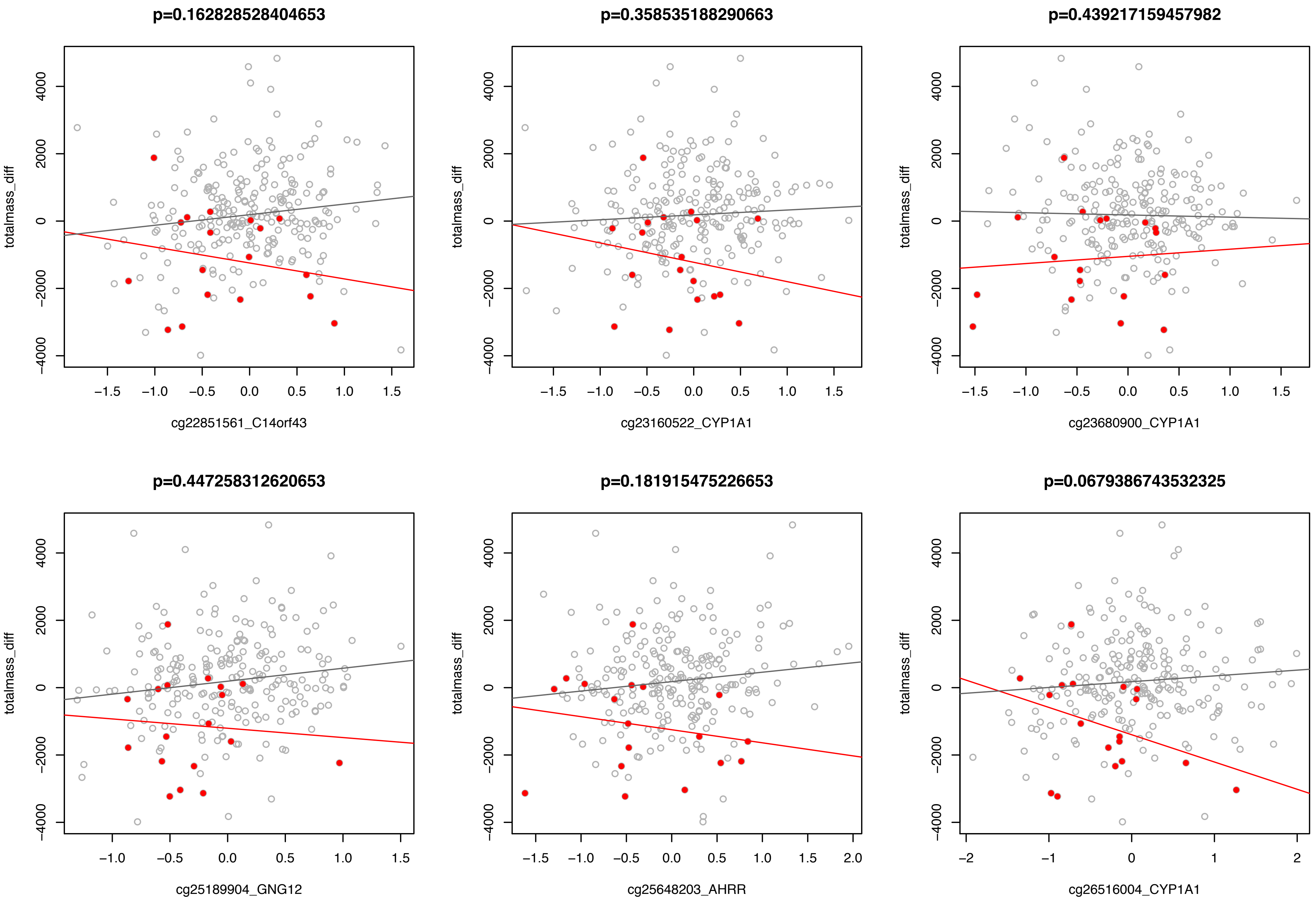
p=0.162828528404653
p=0.358535188290663
p=0.439217159457982
p=0.447258312620653
p=0.181915475226653
p=0.0679386743532325
totalmass_diff
cg22851561_C14orf43
cg23160522_CYP1A1
cg23680900_CYP1A1
cg25189904_GNG12
cg25648203_AHRR
cg26516004_CYP1A1

Figure S5

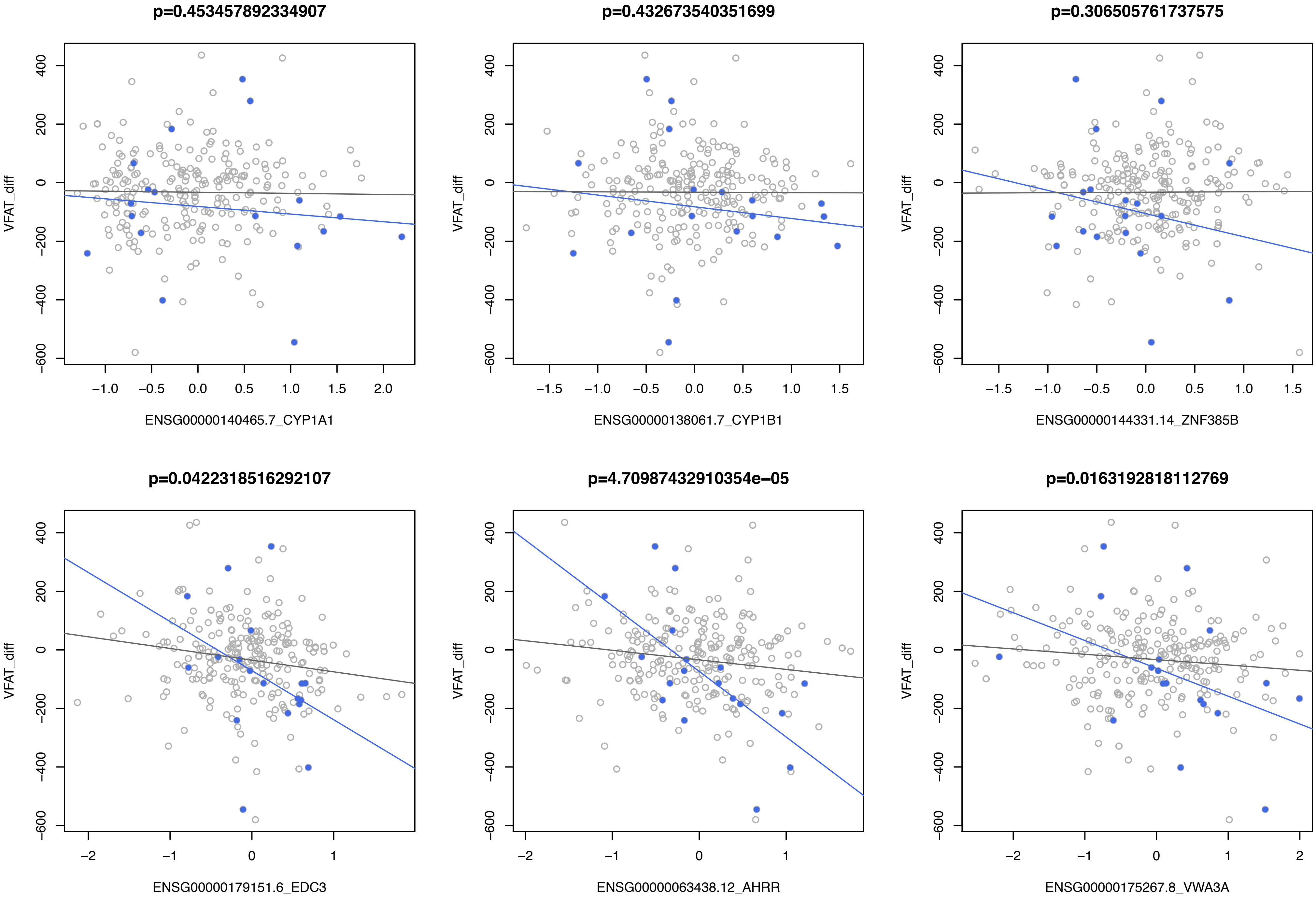
p=0.453457892334907
VFAT_diff
ENSG00000140465.7_CYP1A1
p=0.432673540351699
VFAT_diff
ENSG00000138061.7_CYP1B1
p=0.306505761737575
VFAT_diff
ENSG00000144331.14_ZNF385B
p=0.0422318516292107
VFAT_diff
ENSG00000179151.6_EDC3
p=4.70987432910354e−05
VFAT_diff
ENSG00000063438.12_AHRR
p=0.0163192818112769
VFAT_diff
ENSG00000175267.8_VWA3A

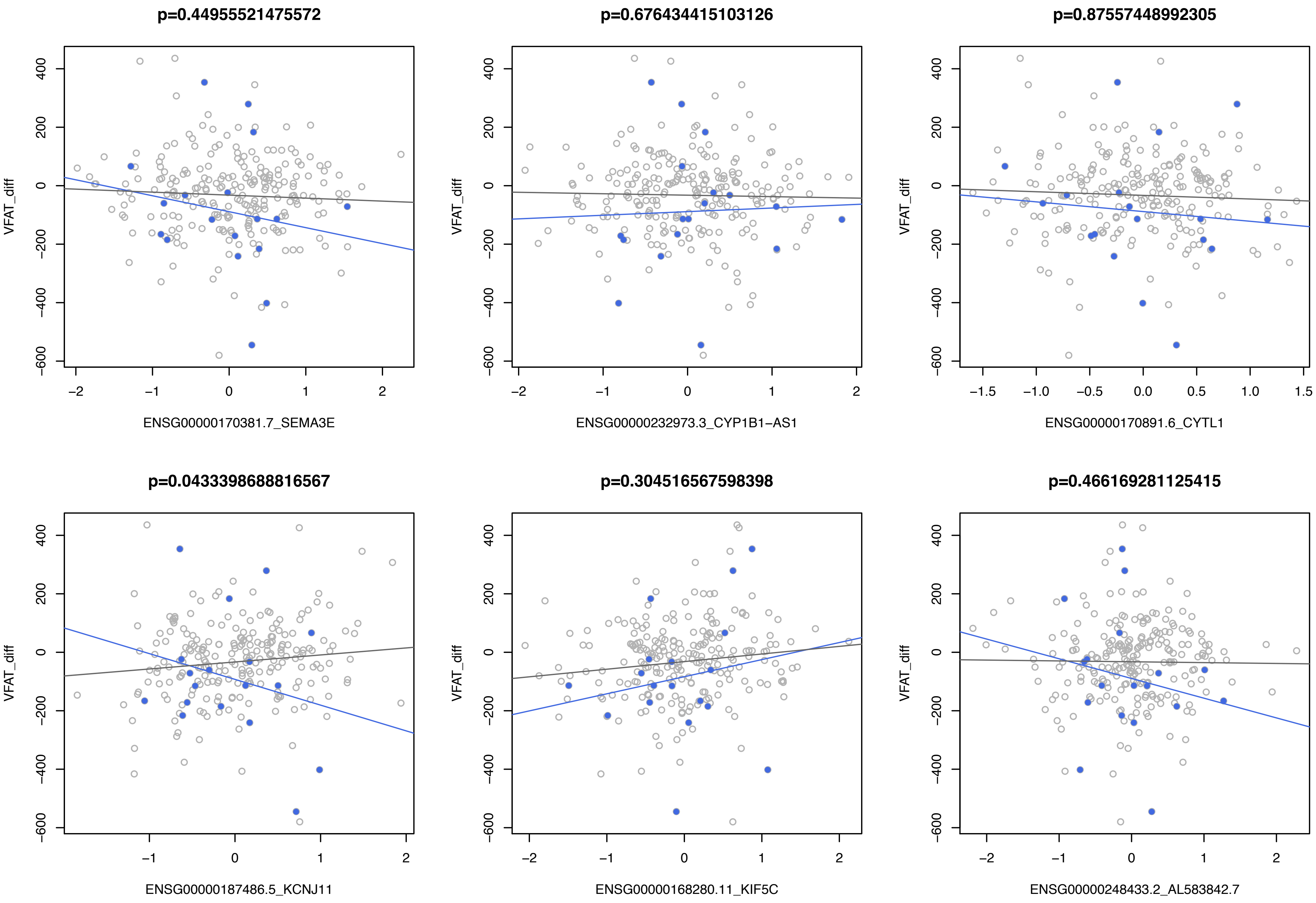
p=0.449555521475572
VFAT_diff
ENSG00000170381.7_SEMA3E
p=0.676434415103126
VFAT_diff
ENSG00000232973.3_CYP1B1−AS1
p=0.87557448992305
VFAT_diff
ENSG00000170891.6_CYTL1
p=0.0433398688816567
VFAT_diff
ENSG00000187486.5_KCNJ11
p=0.304516567598398
VFAT_diff
ENSG00000168280.11_KIF5C
p=0.466169281125415
VFAT_diff
ENSG00000248433.2_AL583842.7

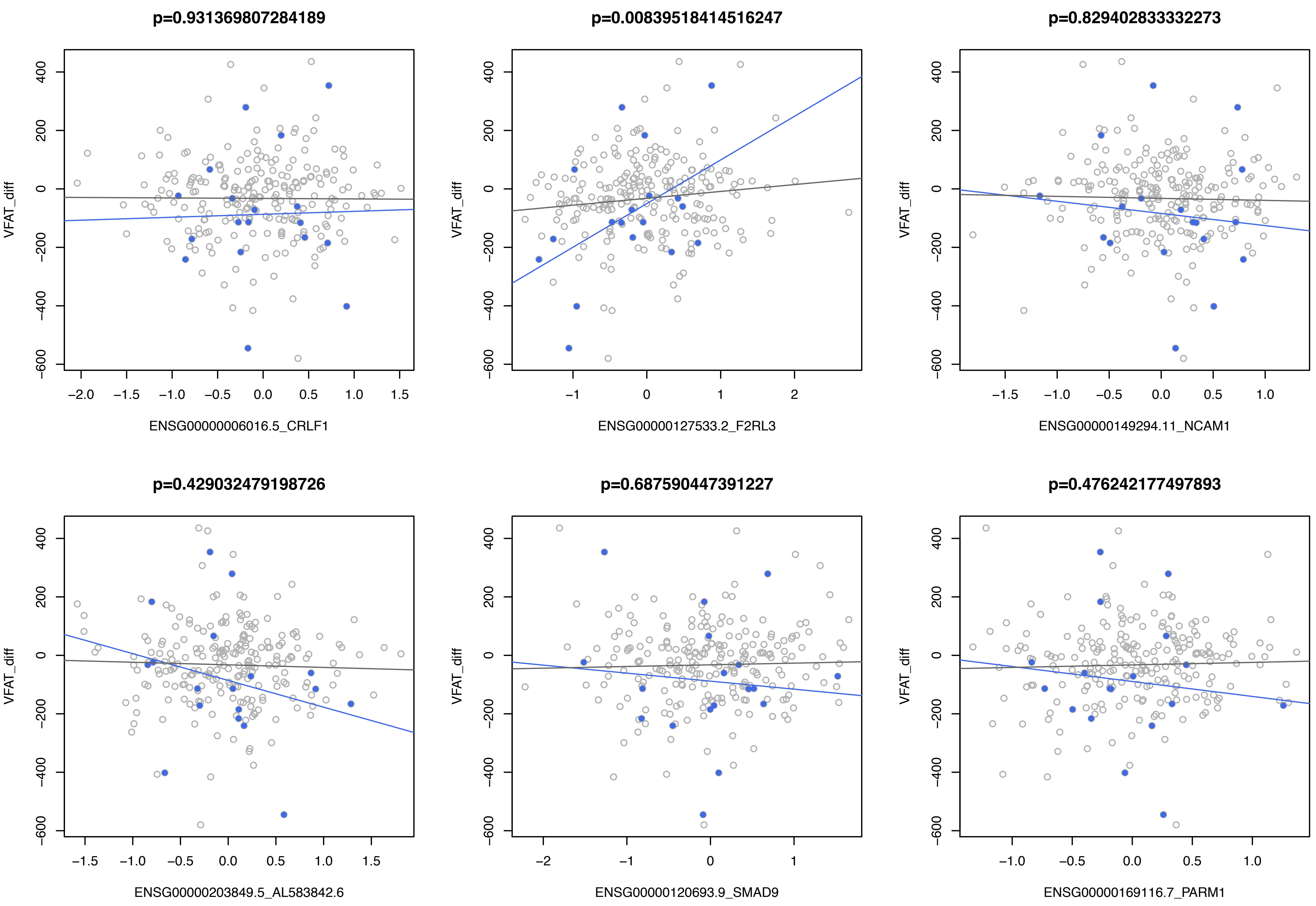
p=0.931369807284189
VFAT_diff
ENSG00000006016.5_CRLF1
p=0.00839518414516247
VFAT_diff
ENSG00000127533.2_F2RL3
p=0.829402833332273
VFAT_diff
ENSG00000149294.11_NCAM1
p=0.429032479198726
VFAT_diff
ENSG00000203849.5_AL583842.6
p=0.687590447391227
VFAT_diff
ENSG00000120693.9_SMAD9
p=0.476242177497893
VFAT_diff
ENSG00000169116.7_PARM1

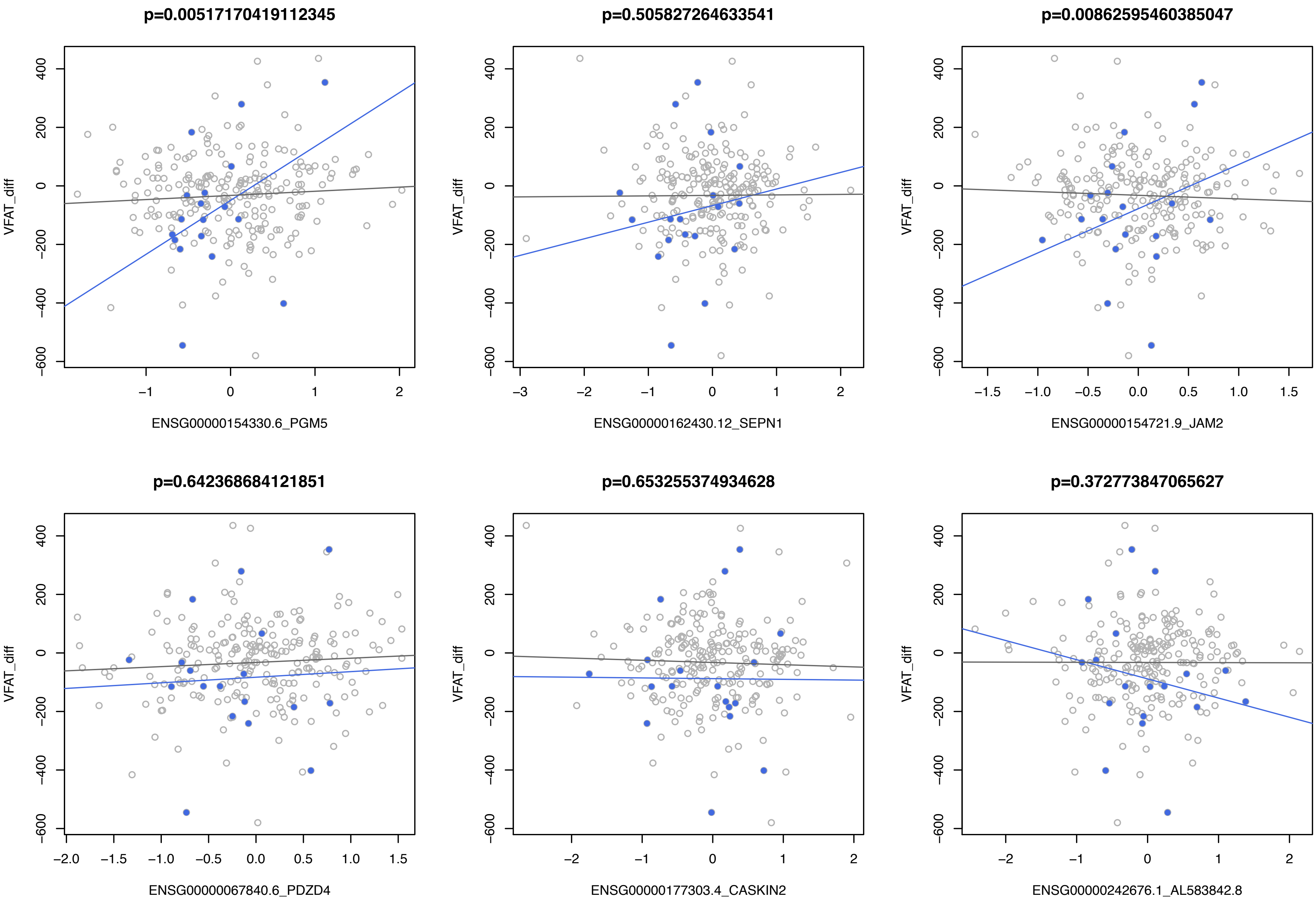
p=0.00517170419112345
VFAT_diff
400
200
0
−200
−400
−600
−1
0
1
2
ENSG00000154330.6_PGM5
p=0.505827264633541
VFAT_diff
−3
−2
−1
0
1
2
ENSG00000162430.12_SEPN1
p=0.00862595460385047
VFAT_diff
−1.5
−1.0
−0.5
0.0
0.5
1.0
1.5
ENSG00000154721.9_JAM2
p=0.642368684121851
VFAT_diff
−2.0
−1.5
−1.0
−0.5
0.0
0.5
1.0
1.5
ENSG00000067840.6_PDZD4
p=0.653255374934628
VFAT_diff
−2
−1
0
1
2
ENSG00000177303.4_CASKIN2
p=0.372773847065627
VFAT_diff
−2
−1
0
1
2
ENSG00000242676.1_AL583842.8

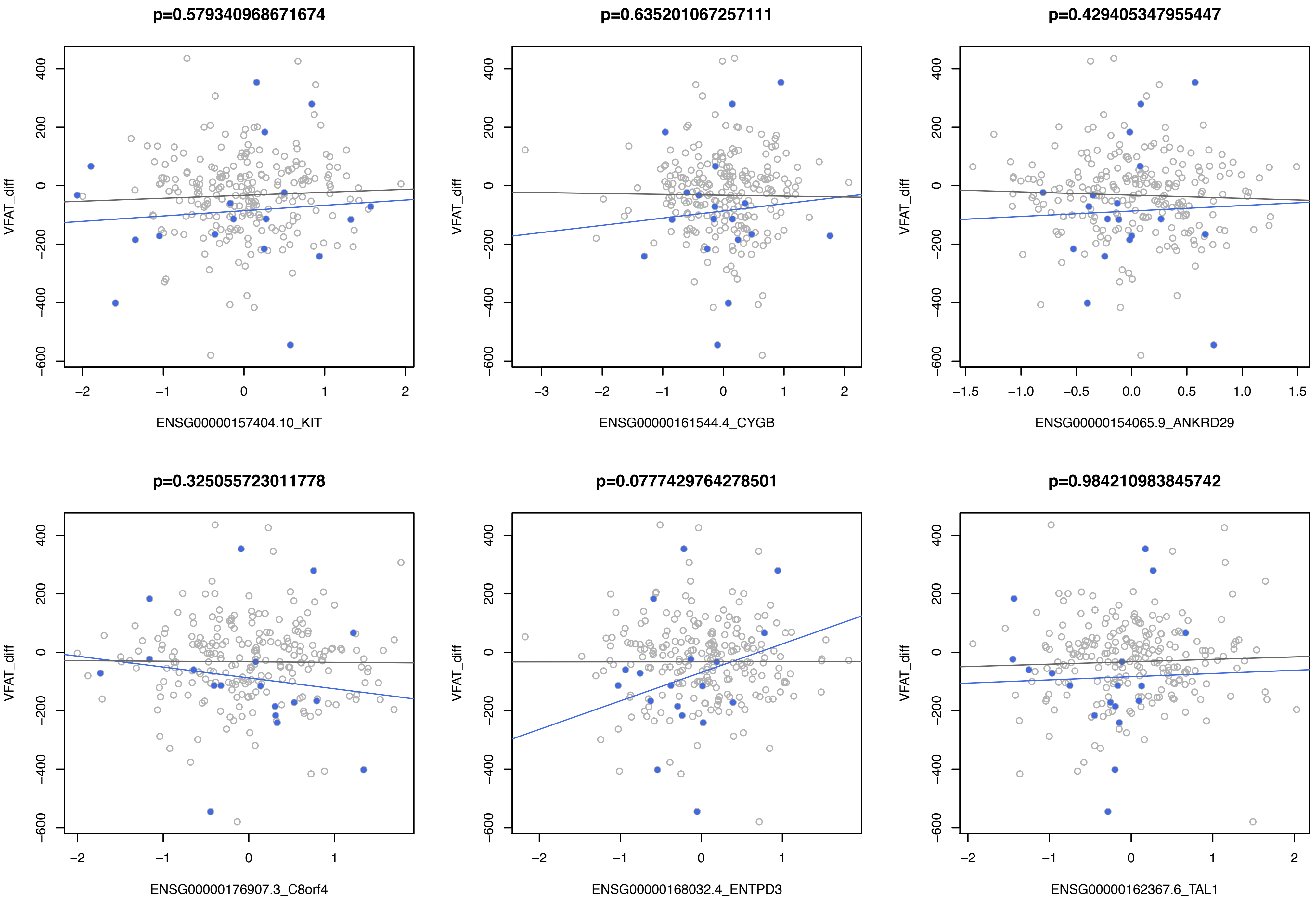
p=0.579340968671674
VFAT_diff
ENSG00000157404.10_KIT
p=0.635201067257111
VFAT_diff
ENSG00000161544.4_CYGB
p=0.429405347955447
VFAT_diff
ENSG00000154065.9_ANKRD29
p=0.325055723011778
VFAT_diff
ENSG00000176907.3_C8orf4
p=0.0777429764278501
VFAT_diff
ENSG00000168032.4_ENTPD3
p=0.984210983845742
VFAT_diff
ENSG00000162367.6_TAL1

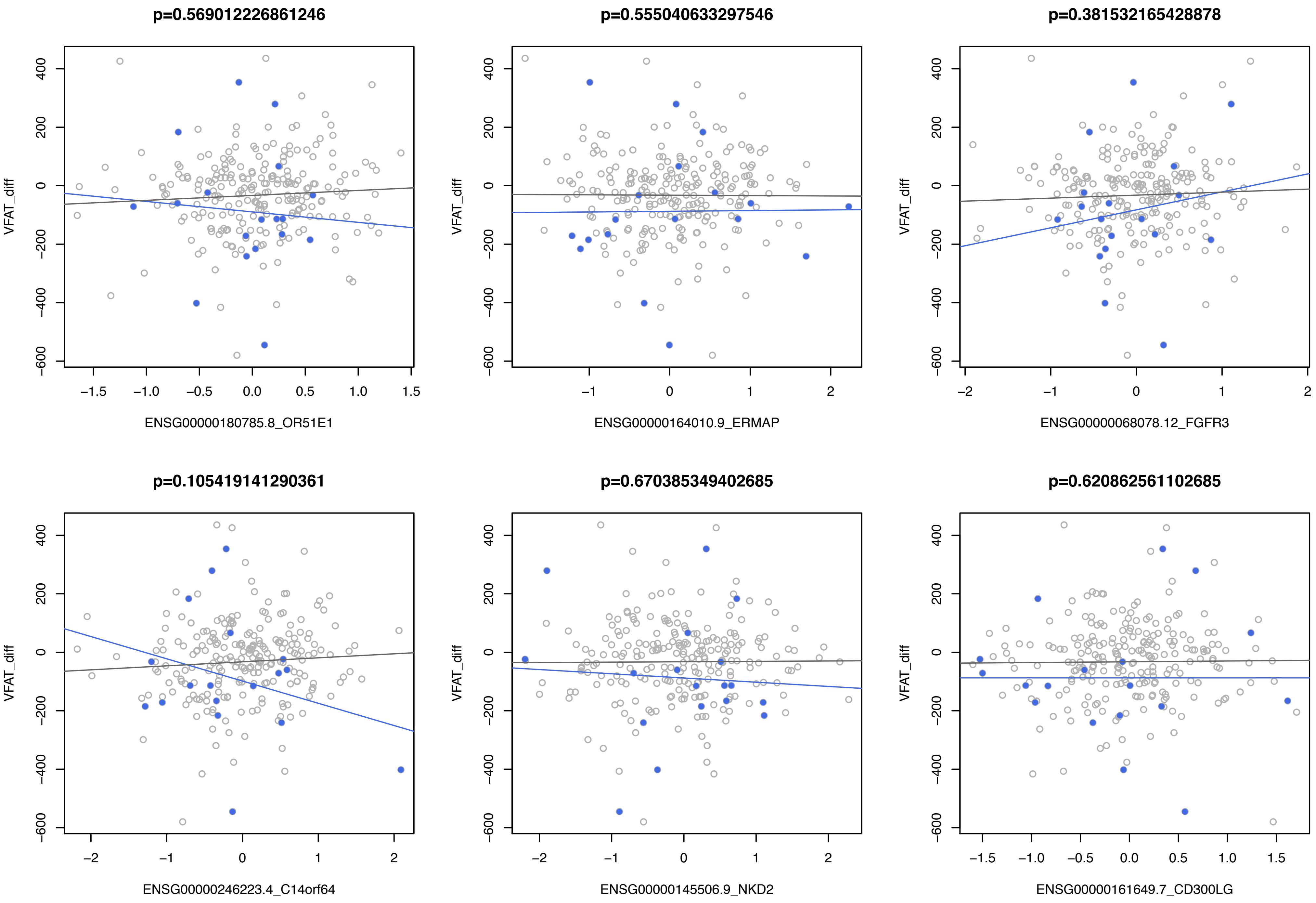

p=0.569012226861246
VFAT_diff
400
200
0
−200
−400
−600
−1.5
−1.0
−0.5
0.0
0.5
1.0
1.5
ENSG00000180785.8_OR51E1
p=0.555040633297546
VFAT_diff
−1
0
1
2
ENSG00000164010.9_ERMAP
p=0.381532165428878
VFAT_diff
−2
−1
0
1
2
ENSG00000068078.12_FGFR3
p=0.105419141290361
VFAT_diff
−2
−1
0
1
2
ENSG00000246223.4_C14orf64
p=0.670385349402685
VFAT_diff
−2
−1
0
1
2
ENSG00000145506.9_NKD2
p=0.620862561102685
VFAT_diff
−1.5
−1.0
−0.5
0.0
0.5
1.0
1.5
ENSG00000161649.7_CD300LG

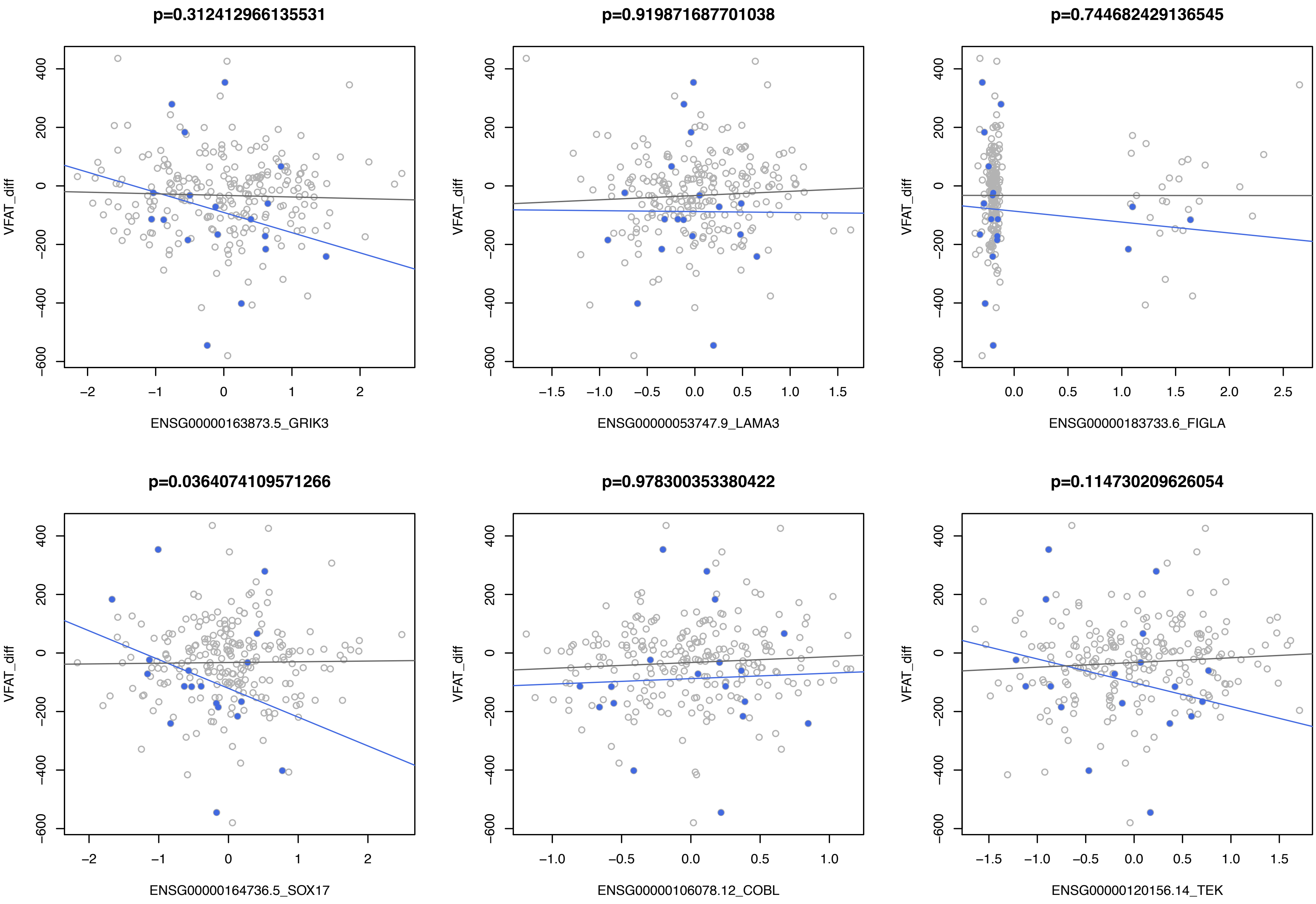
p=0.312412966135531
VFAT_diff
ENSG00000163873.5_GRIK3
p=0.919871687701038
VFAT_diff
ENSG00000053747.9_LAMA3
p=0.744682429136545
VFAT_diff
ENSG00000183733.6_FIGLA
p=0.0364074109571266
VFAT_diff
ENSG00000164736.5_SOX17
p=0.978300353380422
VFAT_diff
ENSG00000106078.12_COBL
p=0.114730209626054
VFAT_diff
ENSG00000120156.14_TEK

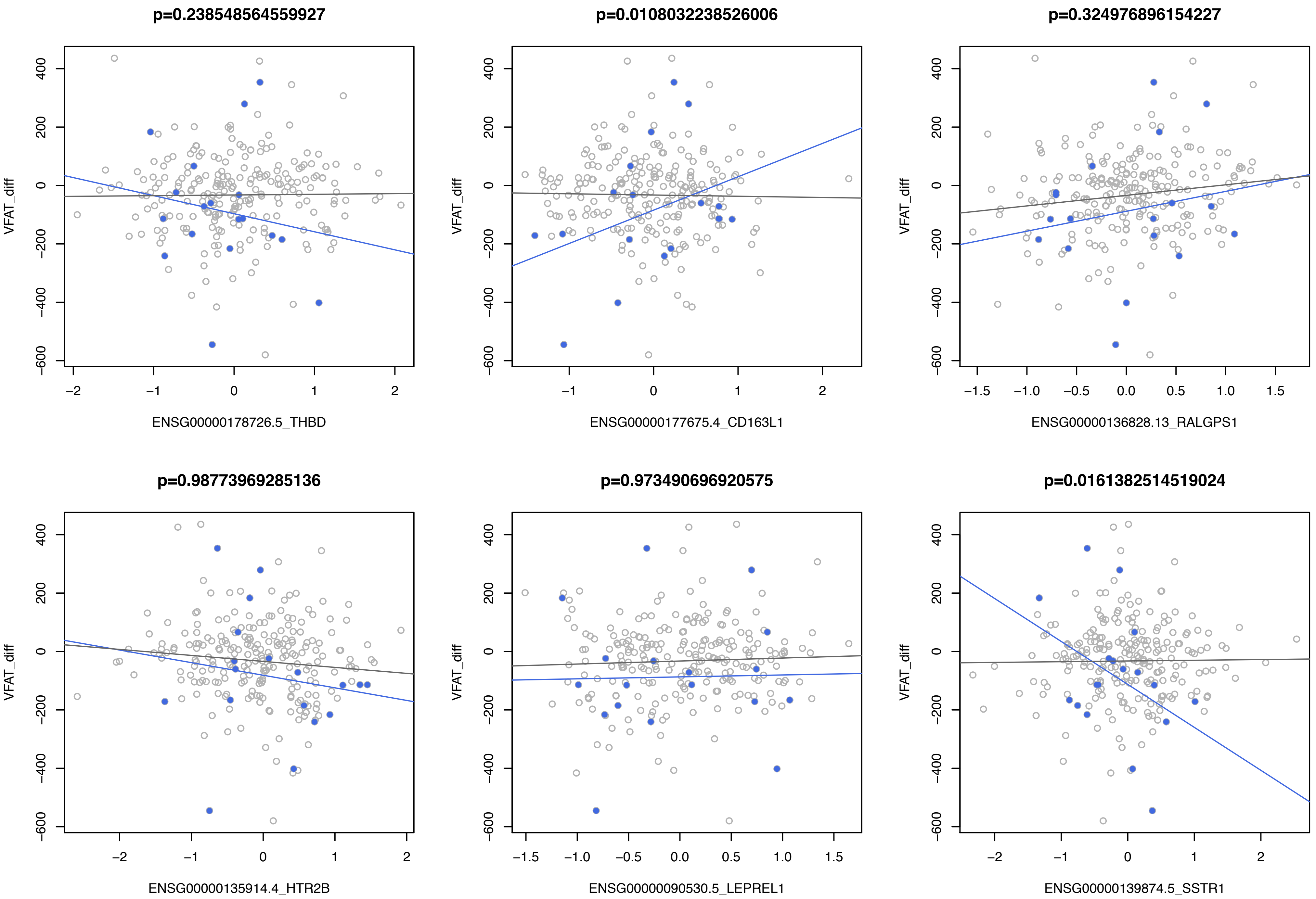

p=0.238548564559927
VFAT_diff
ENSG00000178726.5_THBD
p=0.0108032238526006
VFAT_diff
ENSG00000177675.4_CD163L1
p=0.324976896154227
VFAT_diff
ENSG00000136828.13_RALGPS1
p=0.98773969285136
VFAT_diff
ENSG00000135914.4_HTR2B
p=0.973490696920575
VFAT_diff
ENSG00000090530.5_LEPREL1
p=0.0161382514519024
VFAT_diff
ENSG00000139874.5_SSTR1

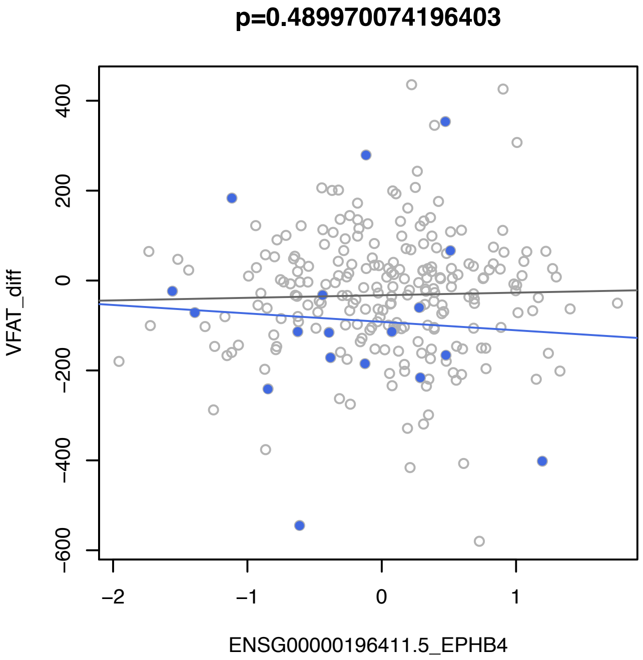
p=0.489970074196403
VFAT_diff
ENSG00000196411.5_EPHB4

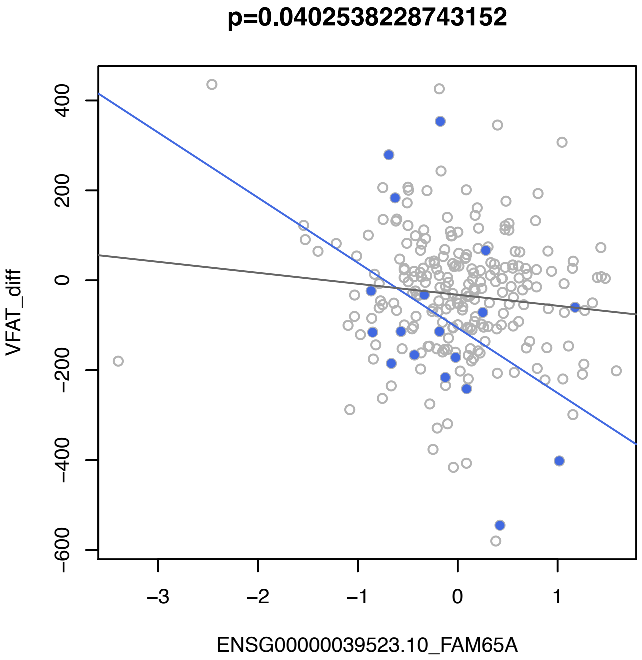
p=0.0402538228743152
VFAT_diff
ENSG00000039523.10_FAM65A

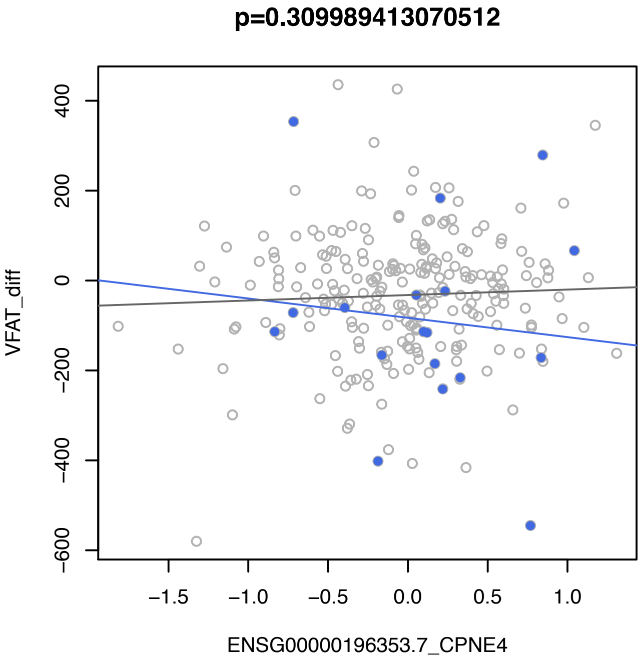
p=0.309989413070512
VFAT_diff
ENSG00000196353.7_CPNE4

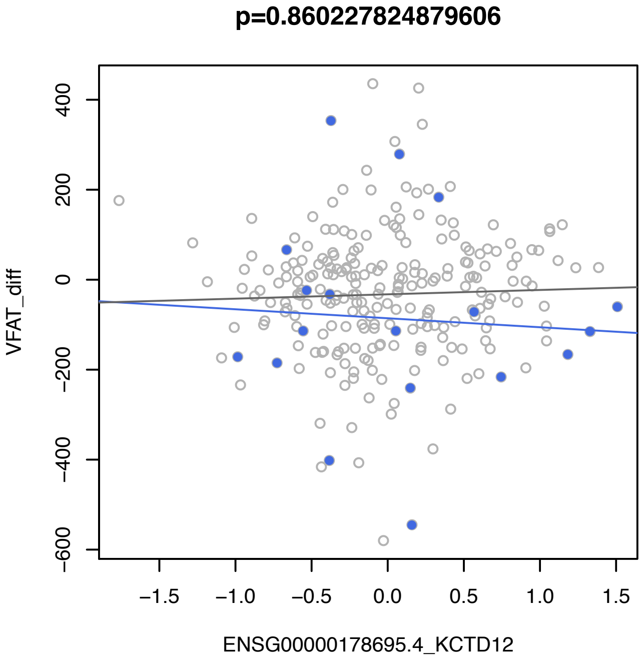
p=0.860227824879606
VFAT_diff
ENSG00000178695.4_KCTD12
